# Small-Molecule Inhibition of Androgen Receptor Dimerization as a Strategy against Prostate Cancer

**DOI:** 10.1021/acscentsci.2c01548

**Published:** 2023-03-08

**Authors:** Weitao Fu, Hao Yang, Chenxian Hu, Jianing Liao, Zhou Gong, Minkui Zhang, Shuai Yang, Shangxiang Ye, Yixuan Lei, Rong Sheng, Zhiguo Zhang, Xiaojun Yao, Chun Tang, Dan Li, Tingjun Hou

**Affiliations:** + College of Pharmaceutical Sciences, Zhejiang University, Hangzhou 310058, Zhejiang, China; ‡ Department of Computer-Aided Drug Design, Jiangsu Vcare PharmaTech Co. Ltd., Nanjing 211800, China; § Institute of Zhejiang University - Quzhou, Zhejiang University, Quzhou 324000, Zhejiang, China; ∥ Polytechnic Institute, Zhejiang University, Hangzhou 310015, Zhejiang, China; ⊥ Innovation Academy for Precision Measurement Science and Technology, Chinese Academy of Sciences, Wuhan 430071, Hubei, China; # University of Chinese Academy of Sciences, Beijing 100049, China; 7 Wuhan National Laboratory for Optoelectronics, Huazhong University of Science and Technology, Wuhan 430074, Hubei, China; 8 Jinhua Institute of Zhejiang University, Jinhua 321000, Zhejiang, China; 9 Key Laboratory of Biomass Chemical Engineering of Ministry of Education, College of Chemical and Biological Engineering, Zhejiang University, Hangzhou 310027, Zhejiang, China; 10 Dr. Neher’s Biophysics Laboratory for Innovative Drug Discovery, Macau Institute for Applied Research in Medicine and Health, State Key Laboratory of Quality Research in Chinese Medicine, Macau University of Science and Technology, Macau 999078, China; 11 Beijing National Laboratory for Molecular Sciences, College of Chemistry and Molecular Engineering, and Center for Quantitate Biology, PKU-Tsinghua Center for Life Science, Academy for Advanced Interdisciplinary Studies, Peking University, Beijing 100871, China

## Abstract

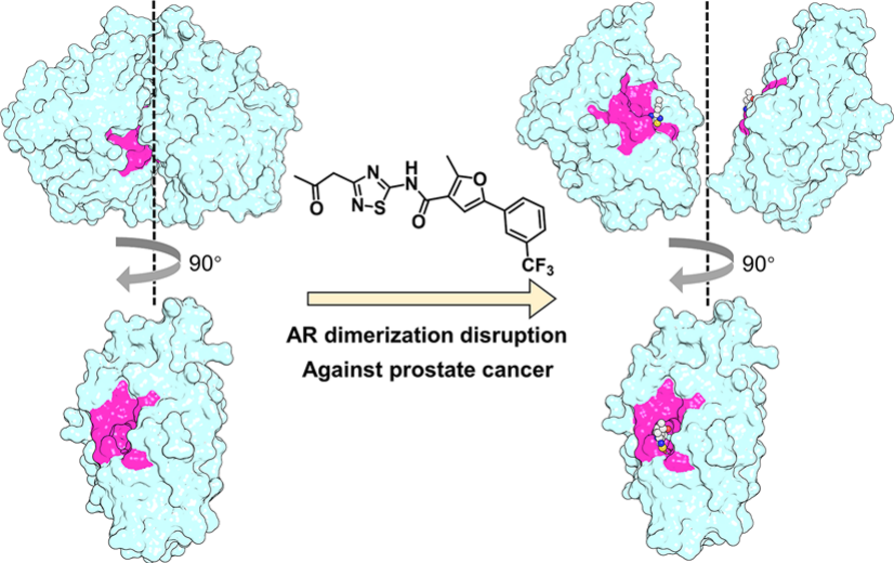

The clinically used androgen receptor (AR) antagonists for the treatment of prostate cancer (PCa) are all targeting the AR ligand binding pocket (LBP), resulting in various drug-resistant problems. Therefore, a new strategy to combat PCa is urgently needed. Enlightened by the gain-of-function mutations of androgen insensitivity syndrome, we discovered for the first time small-molecule antagonists toward a prospective pocket on the AR dimer interface named the dimer interface pocket (DIP) via molecular dynamics (MD) simulation, structure-based virtual screening, structure–activity relationship exploration, and bioassays. The first-in-class antagonist M17-B15 targeting the DIP is capable of effectively disrupting AR self-association, thereby suppressing AR signaling. Furthermore, M17-B15 exhibits extraordinary anti-PCa efficacy in vitro and also in mouse xenograft tumor models, demonstrating that AR dimerization disruption by small molecules targeting the DIP is a novel and valid strategy against PCa.

## Abstract 2

Small molecules targeting the dimer interface pocket located at the ligand-binding domain of androgen receptor (AR) are capable of suppressing AR signaling and combating prostate cancer.

## Introduction

Androgens are responsible for normal physiology, development, and metabolism of reproductive and nonreproductive systems.^1,2^ Testosterone and its metabolite 5β-dihydrotestosterone (DHT) are the two most active androgens, which can form direct interactions with androgen receptor (AR). AR is a member of the nuclear receptor (NR) family of ligand-dependent transcription factors, and it contains four functional modules, including a large N-terminal domain (NTD), a DNA-binding domain (DBD), a short hinge region, and a C-terminal ligand-binding domain (LBD).^3^ The binding of testosterone or DHT to the ligand binding pocket (LBP) of the AR LBD triggers a conformational change of AR that in turn promotes the dissociation of AR from heat shock proteins, and AR dimerization, and transport of AR from the cytosol into the cell nucleus. Subsequently, the AR dimer binds to a specific region of DNA known as the androgen response element (ARE) to activate or repress specific gene transcription.^3−5^ Abnormal AR activity is associated with various diseases, such as prostate cancer (PCa), androgen insensitivity syndrome (AIS) and spinal bulbar muscular atrophy (SBMA).^1,6−8^

AIS is the most common cause of the 46,XY disorder in sex development.^9−11^ Point mutations within the AR LBD are frequently described in this disease.^12^ The AR LBD possesses a three-layer sandwich fold architecture (Figure S1A). The two AR LBD monomers in the homodimer are arranged “head-to-head” and form a large dimer interface by centering the helix 5 (H5). Nearly 40 different AIS-related mutations have been reported on the AR dimer interface.^12,13^ However, so far, how the mutations on the AR dimer interface affect the biological functions of AR remains poorly understood.

In this study, we report the discovery process of a series of novel thiadiazole-amide-based small molecules that block the AR LBD dimerization as our granted patent “CN113444081B”.^14^ At the beginning, we explored the structural ensembles of the wild type AR LBD and two AIS-associated mutants with the mutations (W751R and F754V) located at the dimer interface by molecular dynamics (MD) simulations and small-angle X-ray scattering (SAXS). The simulation and experimental results illustrate that the AR LBD homodimer can be easily disrupted by either W751R or F754V. Therefore, we speculated that blocking the AR dimerization by small molecule antagonists targeting the interface is probably favorable to combat PCa. Interestingly, an AR LBD dimer interface pocket (DIP) was discovered by our theoretical predictions. To prove the druggability of the DIP, an integrative structure-based virtual screening was performed and a novel hit compound M17 was identified. Then through a four-step structure optimization, M17-B15 with >31-fold improved inhibitory activity on AR transcriptional function was discovered. M17-B15 exhibited potent anti-PCa efficacy without detectable toxicity, and it can specifically target to AR rather than its phylogenetically related nuclear receptors. Collectively, our findings demonstrate that AR dimerization disruption by small molecules targeting the DIP is a novel and valid strategy to develop new classes of AR antagonists against PCa.

## Results and Discussion

### AIS Mutations Disrupt the AR LBD Dimerization

**Figure 1:**
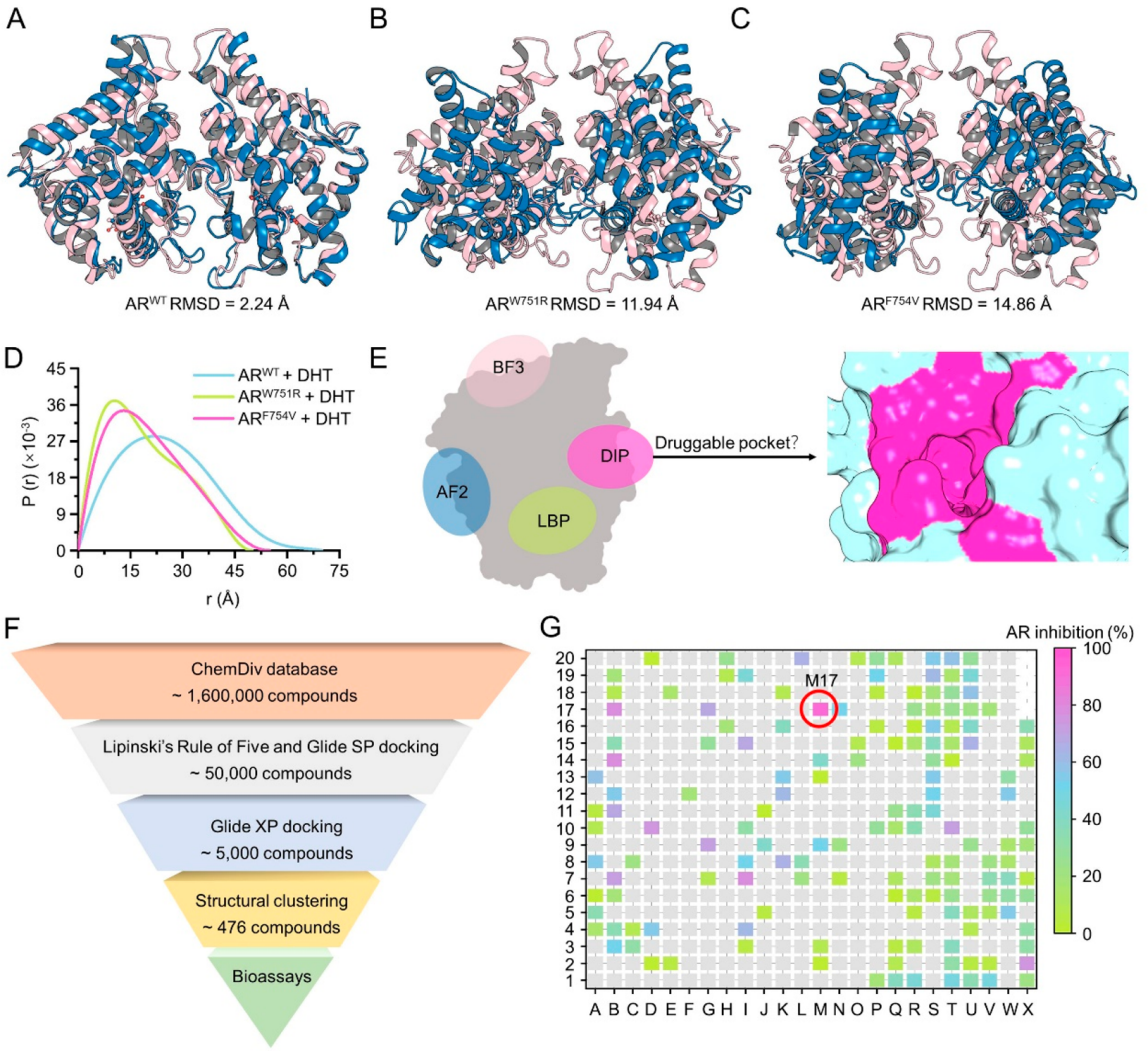
Novel dimer interface pocket (DIP) at the dimer interface of the AR LBD and the discovery of M17. The alignments of the initial (magenta) and last snapshot (blue) from the MD trajectories of AR^WT^ (A), AR^W751R^ (B), and AR^F754V^ (C). (D) Pair-distance distribution function versus interatomic vector length of AR^WT^, AR^W751R^, and AR^F754V^ analyzed by SAXS. (E) Newly discovered DIP at the dimer interface of the AR LBD. (F) Workflow of the structure-based virtual screening (SBVS) protocol to screen novel hits toward the AR DIP. (G) Assessment of the AR antagonistic activities of the 476 compounds from SBVS.

We explored the structural ensembles of the wild type AR LBD (AR^WT^) and two AIS-associated mutants with mutations located in H5 (Figure S1B), i.e., W751R (AR^W751R^ with changed hydrophobicity) and F754V (AR^F754V^ with reduced hydrophobicity and bulkiness), by molecular dynamics (MD) simulations.^15,16^ Analysis of the time evolution of the root-mean square deviations (RMSDs) of the protein backbone atoms of the AR^WT^ dimer confirmed that the simulated system is equilibrated after ∼260 ns (Figure S2A) and the two monomers (mono A and mono B) are stable (Figure S2B–C). However, the RMSDs for the AR^W751R^ and AR^F754V^ dimers are both dramatically fluctuant, though those for each monomer are stable (Figure S2D–I). The alignment of the initial structure (magenta) and the last snapshot (blue) extracted from the MD trajectory for the AR^WT^ dimer shows a high structural similarity with a RMSD of 2.24 Å (Figure 1A and Movie S1), while those for the AR^W751R^ and AR^F754V^ dimers exhibit significant structural differences with RMSDs of 11.94 and 14.86 Å, respectively (Figure 1B–C and Movies S2–3). Based on the theoretical predictions, the mutations disrupt the AR LBD dimerization and lead to self-dissociation.

The predictions were then confirmed by the small-angle X-ray scattering (SAXS) technique, which gives the overall dimension and shape silhouette of the biomacromolecule. As shown in Figure 1D, the maximum end-to-end distance (*D*_max_) of the AR^W751R^ or AR^F754V^ mutant bound with DHT is much smaller than that of AR^WT^, suggesting a compaction of the protein system. As each AR monomer is rigid, the compaction can only be explained by the emergence of the AR LBD monomer. In addition, *D*_max_ of AR^W751R^ is almost identical to that of AR^F754V^, suggesting that neither AR^W751R^ nor AR^F754V^ stably exists in the form of a homodimer. Considering that the AR dimerization is essential for AR transcriptional activity, blocking the AR dimerization by small-molecule antagonists targeting the dimer interface can be a powerful way to combat PCa.

### Identification of the DIP and a Novel Hit M17

We then analyzed the dimer interface and successfully discovered a potential small-molecule binding pocket located at each LBD monomer as illustrated in Figure 1E. The position of the dimer interface pocket (DIP) differs from the other three known binding pockets in the AR LBD, including the LBP, activation function-2 (AF2) site, and binding function-3 (BF3) site. To the best of our knowledge, the site is uncovered for the first time and has never been reported before.^17^ Novel AR antagonists toward the DIP are expected to offer a new approach to treat PCa.

Next, the structure-based virtual screening (SBVS) procedure was employed toward the DIP (Figure 1F). Through compound library filtering using Lipinski’s rule-of-five, cascade molecular docking, and structural clustering, a total of 476 structurally diverse compounds were screened out from the ChemDiv database (∼1.6 million compounds) and submitted for AR transcriptional activity assay. As shown in Figure 1G, the optimal compound M17 inhibits 92.71% AR activity at 10 μM. M17 further shows a dose-dependent manner in the inhibition on AR activity (Figure S3A) and androgen-dependent cell line LNCaP viability (Figure S3B). The serum level of prostate-specific antigen (PSA) is usually used for detecting and monitoring the PCa progression and treatment response.^18^ Herein, the secreted PSA from LNCaP cells was evaluated, and the result shows that M17 dose-dependently inhibits the secretion of PSA (Figure S3C). Furthermore, the binding of M17 to the conventional LBP was excluded by a competitive ligand-binding assay (Figure S3D). These results prompted us to investigate the cell viabilities of androgen-independent cell lines (i.e., 22RV1, PC-3, Du145 and C4-2) upon the treatment of M17. As shown in Figures S3E–H, M17 exhibits comparable effects with the marketed second-generation AR antagonist enzalutamide (Enz) on the proliferation of the four cell lines. In addition, the cytotoxicity of M17 to a normal cell line murine embryonic fibroblast (NIH-3T3) was determined and both M17 and Enz show negligible cytotoxicity against NIH-3T3 (Figure S3I). These data support that M17 targets AR but not the LBP with high safety profile, and it is worth chemical optimization.

### Structural Optimization of M17 Generated M17-B15

**Figure 2:**
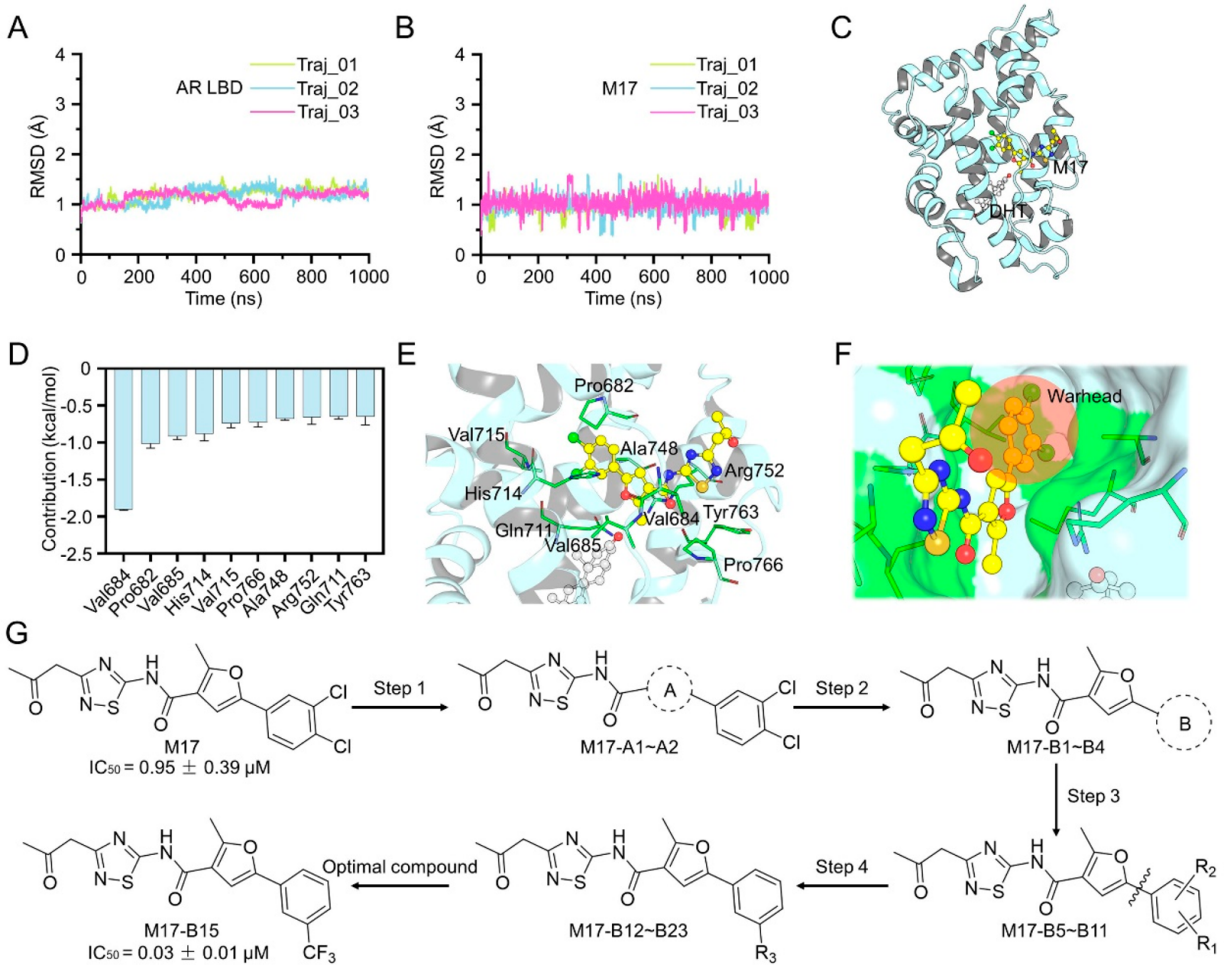
MD simulations of M17 bound to the AR LBD monomer and the structural optimization of M17. (A) RMSDs of the backbone C_α_ atoms of the AR LBD monomer. (B) RMSDs of the heavy atoms of M17. (C) Overview the predicted binding mode of M17 bound to the DIP of the AR LBD. (D) Predicted key residues in the DIP to the binding of M17. (E) Detailed view of the key residues to the binding of M17. (F) M17 in the DIP is represented as the surface. (G) Four-step structure–activity relationship (SAR) explorations of M17 lead to the discovery of >31-fold more potent AR antagonist M17-B15.

To guide structural optimization of M17, the binding mode of M17 in the DIP was further assessed by MD simulations. The RMSD evolutions of the protein backbone atoms of the AR LBD and the heavy atoms of M17 illustrate relatively small fluctuations during the MD simulations (Figures 2A–B). The key residues for the binding of M17 to the AR LBD were highlighted by the MM/GBSA free energy decomposition, and the top-ranked 10 residues are Val684, Pro682, Val685, His714, Val715, Pro766, Ala748, Arg752, Gln711, and Tyr763 (Figures 2C–D). It is suggested that the 5-(3,4-dichlorophenyl)-2-methylfuran moiety of M17 locates in the deep DIP and forms favorable hydrophobic interactions with most of the key residues. The 3-(2-oxopropyl)-1,2,4-thiadiazol moiety is exposed to the solvent and connected to the 5-(3,4-dichlorophenyl)-2-methylfuran moiety by an acylamide linker (Figures 2E–F). Considering that 5-(3,4-dichlorophenyl)-2-methylfuran is close to most of the key residues, a four-step structure–activity relationship (SAR) exploration on this moiety was conducted as illustrated in Figure 2G, Table 1, and Table S1. The AR antagonistic activities were decreased or totally lost when methylfuran (A ring) was replaced by different groups (M17-A2 and M17-A2). We also examined the role of the benzene ring (B ring) in 3,4-dichlorophenyl. The compound with phenyl (M17-B1) exhibits optimal AR antagonistic activity compared with the rest of the compounds (M17-B2 ∼ M17-B4). Subsequently, we designed compounds with various substituents at different positions of phenyl (M17-B5 ∼ M17-B11) and found that the meta-substituted compound M17-B10 is more potent. We thus designed compounds with different substituents at the meta-position of the phenyl (M17-B12 ∼ M17-B23), which led to the discovery of the potent compound M17-B15 (IC_50_ = 0.03 μM). This compound achieved >31-fold improvement in AR transcriptional inhibition compared with the original hit M17 (Figure 2G, Table 1).

**Table 1:**
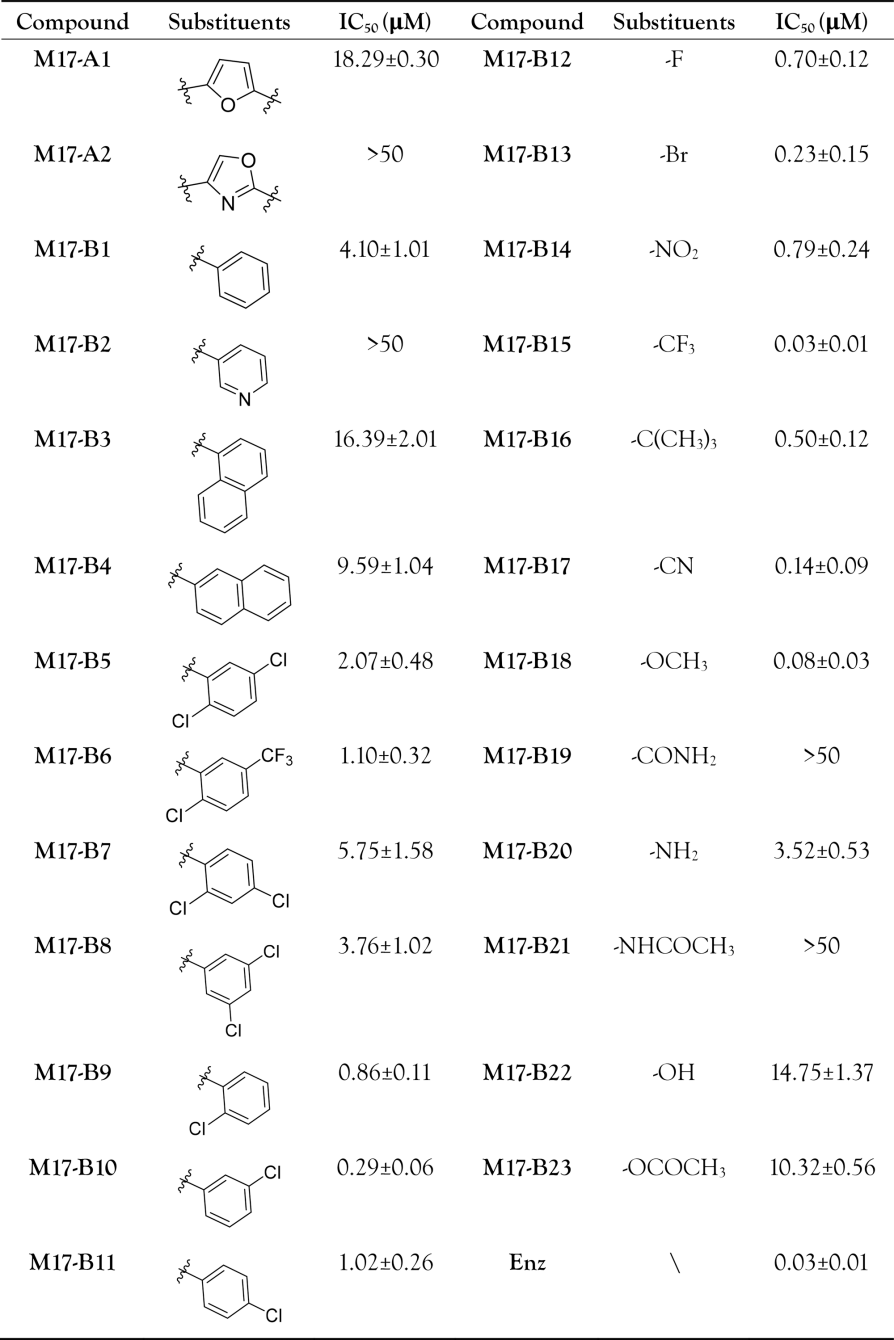
AR Transcriptional Activities of 25 Analogues of M17

### M17-B15 Inhibits the AR Dimerization

**Figure 3:**
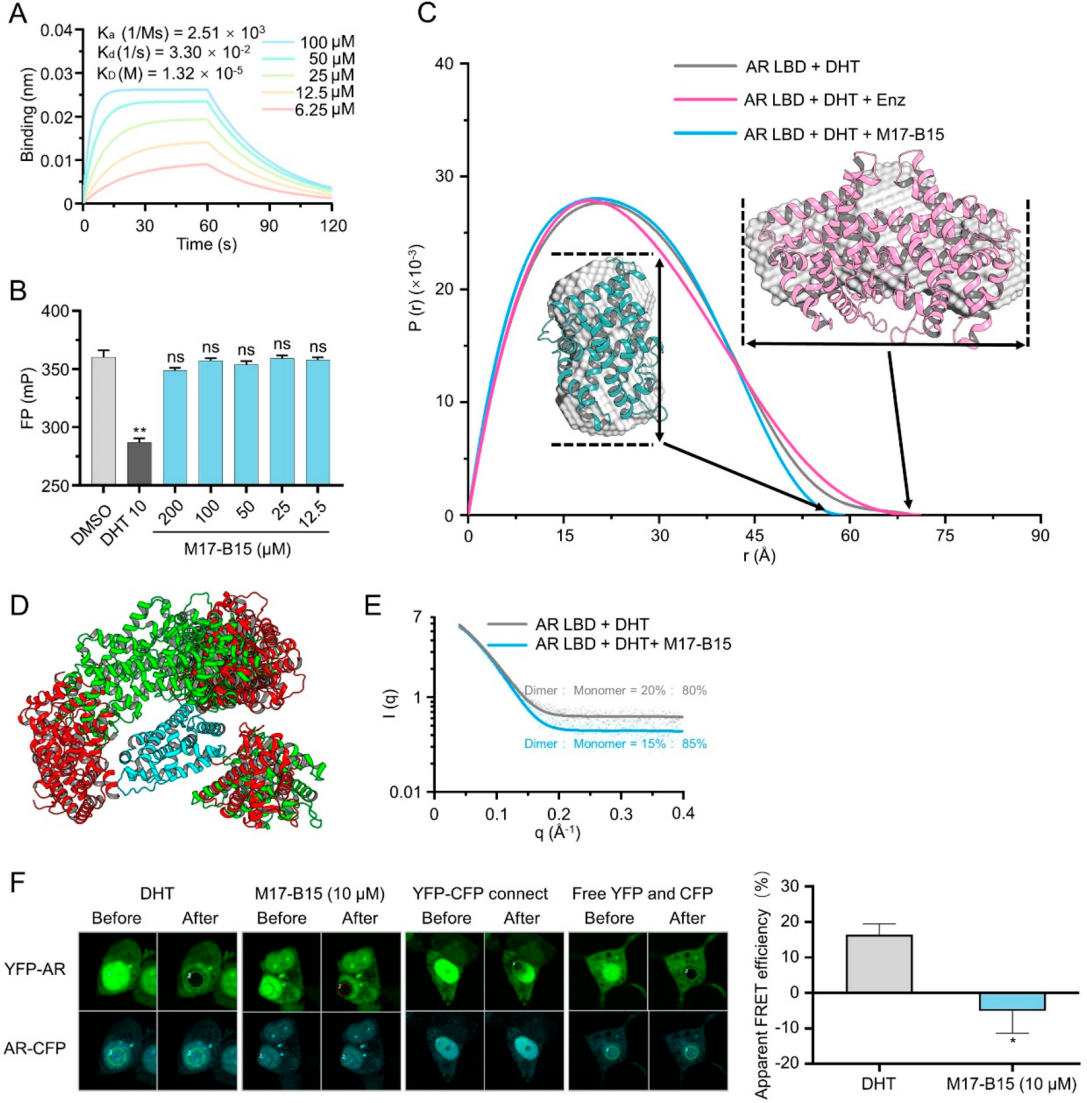
M17-B15 inhibits AR dimerization. (A) Relative binding affinities of M17-B15 to the AR LBP analyzed by the PolarScreen AR competitor assay. (B) Direct interactions between M17-B15 and the purified AR LBD protein assessed by biolayer interferometry (BLI). (C) Pair distance distribution function versus particle radius analyzed by SAXS. (D) Ensemble of the AR LBD dimer conformation with (red) or without (green) M17-B15. Both conformational ensembles contain 7 structures. (E) Ratio (dimer:monomer) of the AR LBD protein with/without M17-B15 calculated from SAXS. (F) Characterization of the AR LBD dimer interactions by FRET. Representative confocal images of HEK293T cells transiently expressing indicate protein with or without M17-B15 in the presence of 10 nM DHT (left). The apparent FRET efficiencies of DHT and M17-B15 were calculated after background subtraction (right panel).

Biolayer interferometry (BLI) experiments were performed to determine the binding affinity of M17-B15 to the purified AR LBD. As shown in Figure 3A, M17-B15 forms direct interactions with the AR LBD in a dose-dependent manner with an equilibrium dissociation constant (*K*_D_) of 1.32 × 10^–5^ M. Similar to M17, M17-B15 does not target the LBP as indicated by the competitive ligand-binding assay (Figure 3B). We thus determined whether M17-B15 inhibits AR dimerization. The SAXS results demonstrate that M17-B15 inhibits the purified AR LBD dimerization with the *D*_max_ value of the AR LBD, corresponding to the overall size of the protein, markedly reduced upon the addition of M17-B15 (Figure 3C). Furthermore, we used chemical cross-linking coupled with mass spectrometry (CXMS) to assess the distance relationships within and between AR LBDs and to characterize the structural dynamics (Tables S2–S3).^19^ The ensemble refinement against the CXMS restraints indicates that 7 conformers in the ensemble can satisfy all the cross-linking pairs (Figure 3D). By traversing the ratio of these dimer to monomer dynamic conformers, we found that the proportion of the dimer is reduced from 20% to 15% upon the addition of M17-B15, which is also consistent with the SAXS data (Figure 3E). Furthermore, the dimeric structure in the AR LBD selected from the CXMS conformers is more heterogeneous in the presence of M17-B15. Together, the data indicate that the addition of M17-B15 reduces the AR LBD dimerization while making the protein more dynamic.

To explore whether M17-B15 can inhibit the AR dimerization at the cellular level, the N-terminal yellow fluorescent protein (YFP)-labeled and C-terminal cyan fluorescent protein (CFP)-labeled AR-LBD plasmids were constructed for the acceptor-bleaching fluorescence resonance energy transfer microscopy (FRET) assay. After treating with 10 nM DHT, the AR LBD dimerization occurred, which induced the YFP- and CFP-labeled AR LBDs to move closer to each other, thereby exhibiting high FRET efficiency. However, lower FRET efficiency was observed in the presence of 10 μM M17-B15 (Figure 3F), indicating that M17-B15 also inhibits the AR-LBD dimerization at the cellular level.

### Potent Anti-PCa Efficacy of M17-B15

Like Enz, M17-B15 shows negligible cytotoxicity against NIH-3T3, human liver cells (Chang), and human gastric epithelial cell (GES-1, Figures S4A–C), and M17-B15 does not show obvious inhibition on the cell viability of 22RV1, PC3, and Du145 (Figures S4D–F). The dose–response curves for M17-B15 and Enz on LNCaP cells are quite similar, which is consistent with their anti-AR transcriptional activities (Figure S4G and Table 1). To estimate the long-term growth inhibition effects of M17-B15, the colony forming assays were performed on LNCaP and 22RV1 cells. Both Enz and M17-B15 show significant inhibitory activities toward the colony formation of LNCaP cells but not 22RV1 (Figure S4H), consistent with the data from the cell viability assays.

**Figure 4:**
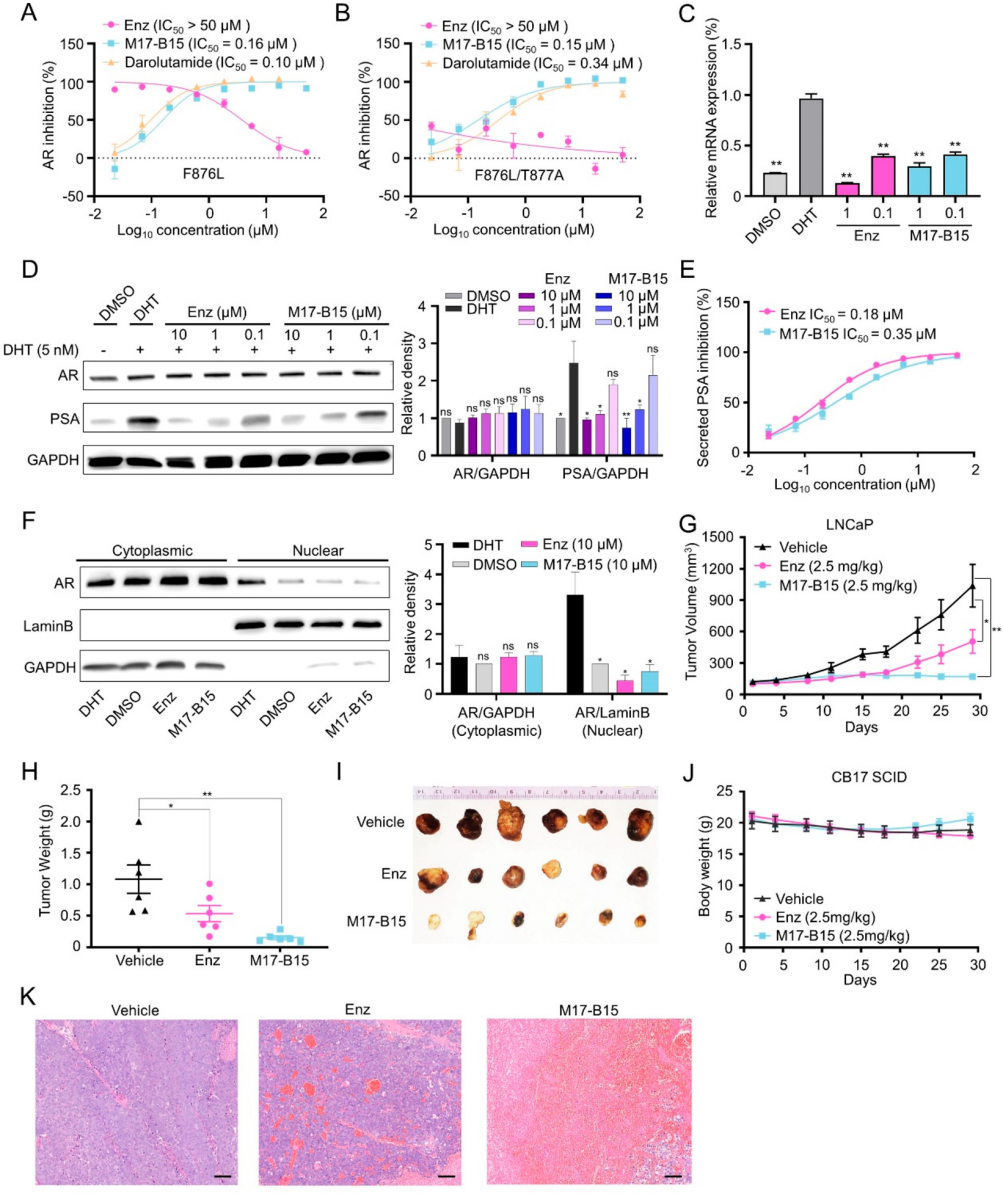
M17-B15 suppresses AR signaling to inhibit PSA expression and is efficacious in mouse xenograft tumor. M17-B15 and darolutamide antagonize FL-AR^F876L^ (A) and FL-AR^F876L/T877A^ (B) with Enz as the control. (C) Relative mRNA expression of PSA in LNCaP cells determined by qPCR. (D) M17-B15 reduces the endogenous PSA levels but does not affect the AR levels in LNCaP cells. The densities of the dimethyl sulfoxide (DMSO) groups were normalized to 1 for each ratio. (E) M17-B15 reduces the PSA levels of LNCaP cells secreted in the cellular media. (F) Nuclear and cytoplasmic extracts were collected from LNCaP cells. GAPDH and LaminB were used as the controls for the cytoplasmic fraction and nuclear fraction, respectively. The densities of the DMSO groups were normalized to 1 for each ratio. M17-B15 (2.5 mg/kg/week, *n* = 6 per group) significantly inhibited tumor volume (G) and tumor weight (H), versus vehicle control (*n* = 6 per group) and positive drug Enz (2.5 mg/kg/week, *n* = 6 per group). (I) Photographs of xenograft tumors harvested at day 29. (J) Body weight of mice in each group. (K) H&E staining of representative sections of xenograft tumors (bar = 100 μm). *P < 0.05, **P < 0.01 vs DHT group for in vitro assays or vs vehicle for in vivo assays; ns, not significant.

A previous study indicated the AR LBP point mutation F876L and F876L/T877A convert Enz from an antagonist to a partial agonist, thus contributing to Enz resistance.^20^ The impact of the full-length AR^F876L^ (FL-AR^F876L^) and AR^F876L/T877A^ (FL-AR^F876L/T877A^) on M17-B15 was evaluated using the dual-luciferase reporter assay. As a comparison, a recently approved AR antagonist darolutamide, which targets the AR LBP and could significantly inhibit the transcriptional activity of the AR F876L and F876L/T877A mutants, was also applied.^21^ As shown in Figures 4A–B, Enz lost its antagonistic activity in both cases, while M17-B15 and darolutamide still exhibited good antagonistic activities with IC_50_ values of 0.16 μM and 0.10 μM for FL-AR^F876L^, and 0.15 μM and 0.34 μM for FL- AR^F876L/T877A^, respectively. In addition, M17-B15 significantly inhibits the DHT-induced transcriptional and translational levels of PSA (Figures 4C–E). The qPCR results show that the mRNA expression of PSA in LNCaP cells was inhibited by both Enz and M17-B15 (Figure 4C). The Western blot results exhibit that both Enz and M17-B15 remarkably inhibited the endogenous PSA but not AR production (Figure 4D). The curve of M17-B15 for the secreted PSA inhibition is dose-dependent and similar to that of Enz (Figure 4E). An important difference between the first- and second-generation AR antagonists is that the latter not only inhibits AR signaling like the former, but also inhibits the AR nuclear translocation. To evaluate the effect of M17-B15 on the DHT-induced translocation of AR from the cytoplasm to the nucleus, the nuclear and cytoplasmic fractions were extracted and analyzed. As shown in Figure 4F, both M17-B15 and Enz inhibit the DHT-induced AR nuclear translocation compared with the DHT-treated group.

The efficacy of M17-B15 in vivo was further evaluated using a mouse xenograft model. Due to the poor solubility, oral administration and intravenous injection were not suitable for M17-B15. As a result, direct intratumor injections of vehicle (saline), Enz (2.5 mg/kg/week), and M17-B15 (2.5 mg/kg/week) were administered to the established subcutaneous xenografts of LNCaP cells. As shown in Figures 4G–I, both Enz and M17-B15 significantly inhibited tumor volume and weight. The tumor growth inhibition (TGI, %) for M17-B15 achieves 83.59% and is much superior to that for Enz (51.35%). In addition, no mortality or significant loss of body weight was observed in any of the treatment groups (Figure 4J). The TGI levels are not well consistent with the experimental data from the cell viability and colony forming assays, which can be explained by the hematoxylin-eosin (H&E) staining results that the abilities of M17-B15 to decrease cell density and number and increase necrotic areas are much better than those of Enz (Figure 4K).

### DIP Can be a Selective Binding Pocket

**Figure 5:**
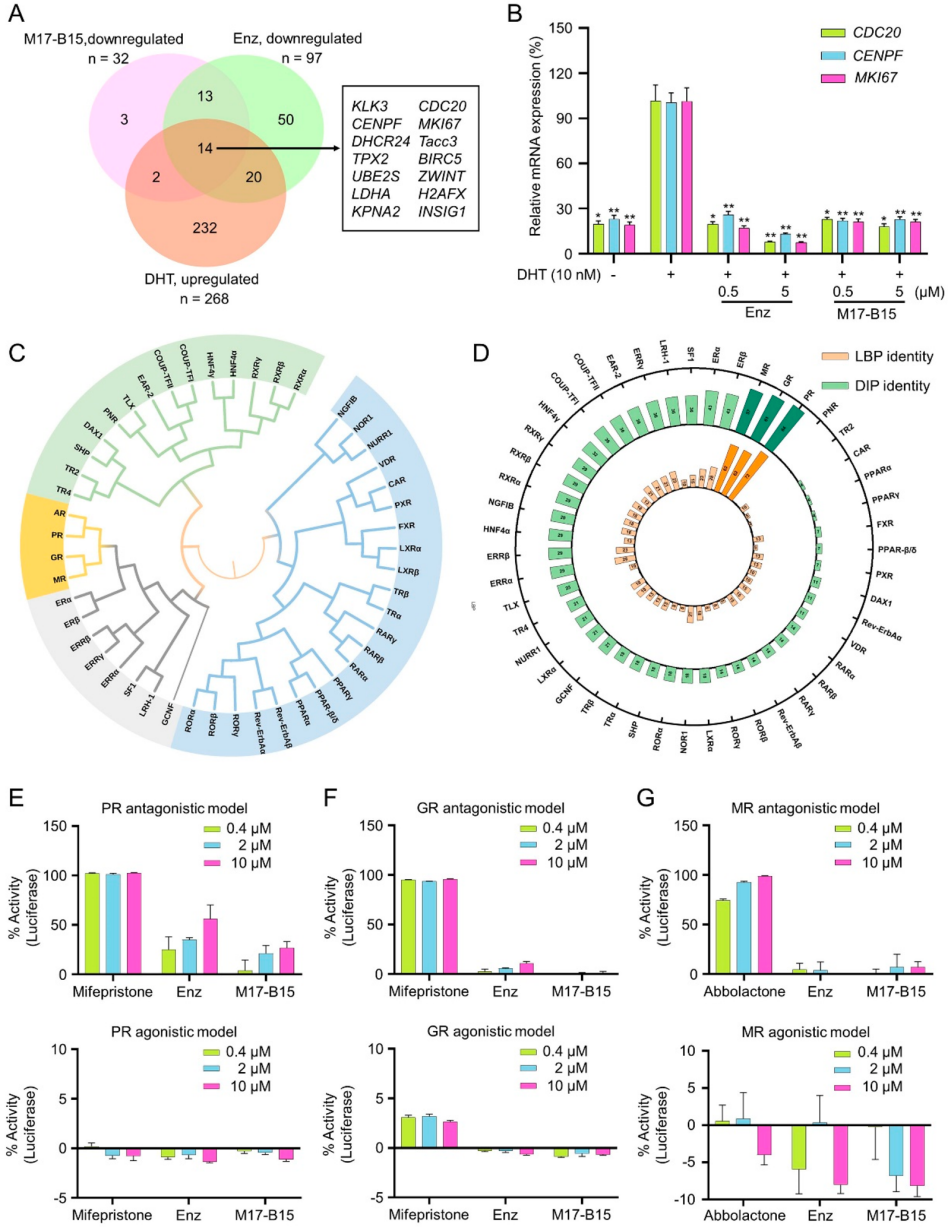
DIP is a selective binding pocket for M17-B15. (A) Venn diagram of DEGs in treated groups of DHT, Enz, and M17-B15. (B) The relative mRNA expression of *CDC20*, *CENPF* and *MKI67* in LNCaP cells determined by qPCR. (C) Phylogenetic tree of the LBDs of 48 human NRs. (D) Sequence identities of the AR LBP and DIP to other 47 NR LBDs. The antagonistic activities (top) and agonistic activities (bottom) of M17-B15 toward PR (E), GR (F), and MR (G). *P < 0.05, **P < 0.01 vs DHT group; ns, not significant.

Considering that AR is indeed a transcriptional factor, RNA-seq was performed on LNCaP cells for M17-B15 with Enz as the control. The results show that DHT is associated with 268 up-regulated and 222 down-regulated genes, Enz is associated with 56 up-regulated and 97 down-regulated genes, and M17-B15 is associated with 6 up-regulated and 32 down-regulated genes (Figures S5A–C). The Venn diagram displays that there are 14 shared differentially expressed genes (DEGs, Figure 5A, Table S4), which are down-regulated in the antagonist-treated groups of Enz and M17-B15 while up-regulated in the DHT-treated group. The corresponding heatmap shows their relative expressions (Figure S5D). Among these DEGs, 4 of them have been reported and their up-regulations are linked to recurrent PCa, including *KLK3* (encoding *PSA*), *CDC20*, *CENPF*, and *MKI67*.^22^ Therefore, we validated the vital genes of *CDC20*, *CENPF*, and *MKI67* by qPCR. It is shown that the treatment by Enz or M17-B15 significantly reduces the expressions of all these genes compared with those by DHT (Figure 5B), suggesting that the results shown in the Venn diagram are reliable. Notably, the up-regulated and down-regulated genes for M17-B15 are less than those for Enz, indicating a more specific and probably safer profile. Thus, it begs an interesting question: whether the DIP can truly be a selective binding pocket for small molecules? Since the NR family is large and the members are highly conserved, the answer is crucial and probably determines the druggability of the pocket.

We analyzed the LBDs of 48 human nuclear receptors (NRs), and their phylogenetic tree was constructed. The LBDs of progesterone receptor (PR), glucocorticoid receptor (GR), and mineralocorticoid receptor (MR) show the highest evolutionary conservation (Figure 5C). That is why PR, GR, and MR have been commonly tested when developing AR antagonists and agonists.^23−25^ Then the sequence identities of the LBPs and DIPs were analyzed, respectively (Figure 5D). It is observed that the conventional LBPs of PR, GR, and MR share high sequence identities to that of AR. However, marketed drugs like Enz can still exhibit high selectivity on the AR LBP, which encourages us to further explore the selectivity of M17-B15 because the DIPs also show high sequence identities among PR, GR, and MR. Luciferase reporter assays toward PR, GR, and MR were performed. The antagonistic activities of mifepristone for PR and GR are potent (>90%) even at the concentration of 0.4 μM, while those for Enz and M17-B15 are moderate for PR and largely ineffective for GR even at the relatively high concentration of 10 μM. These compounds are largely ineffective on the PR and GR agonistic activities (Figures 5E–F). Similarly, the MR antagonistic activity (IC_50_) for abbolactone is potent at the concentration of 0.4–10 μM, while those for Enz and M17-B15 are not observed even at a relatively high concentration of 10 μM. These compounds do not show an obvious effect on the MR agonistic activities (Figure 5G). These results demonstrated that M17-B15 can well distinguish AR from its homologies of PR, GR, and MR. Structural alignment of the DIPs of AR, PR, GR, and MR further indicated that the unique residues of His714, Thr755, and Pro801 in the AR DIP are vital for the high selectivity of M17-B15 toward the AR DIP rather than its homologies (Figure S6).

In addition, the cell viability inhibition of various of human cancer cell lines for Enz and M17-B15 was evaluated, including osteosarcoma (U2OS), breast cancer (MCF-7), hepatocellular carcinoma (HepG2), leukemia (HL60), non-small cell lung cancer (H1299 and A549), colorectal cancer (SW480), glioma (U87), and cervical adenocarcinoma (Hela) cell lines. As shown in Figure S7, with the concentration only as high as 50 μM, mild to moderate inhibitory effects were observed for either Enz (except for U2OS) or M17-B15 (except for A549). Furthermore, both M17-B15 and Enz do not show an obvious impact on the cell cycle and apoptosis of the LNCaP cell line (Figure S8). The above results highlight the potential high AR specificity of M17-B15 and good druggability of the DIP.

## Conclusions

Investigation of the dynamic behavior of AIS mutations at the AR homodimer interface illuminated the strategy of small molecules targeting the protein interface to disrupt AR homodimerization to treat PCa and AR-related diseases. Herein, we identified a potential pocket named the DIP located at the dimer interface near H5, and the druggability of the previously unexploited pocket was validated by a novel small molecule M17-B15. TM17-B15 exhibits potent anti-PCa efficacy in vitro and in vivo, and it specifically targets AR toward its homologues PR, GR, and MR. However, structural optimization is still urgently required for M17-B15, especially to the chemical modification of the solvent-exposed moiety of 3-(2-oxopropyl)-1,2,4-thiadiazol to improve its pharmacokinetic properties. In addition, we believe that the AR DIP will inspire interesting studies on the discovery of new classes of therapeutics including novel proteolysis-targeting chimeras (PROTACs) and molecular glues for treating PCa and AR-related diseases. Moreover, the DIP is as excellent as the conventional target of the LBP in our study, providing a new option for the other NRs to develop variant NR modulators. Overall, this study provided a successful paradigm from gain-of-function mutations to a potential druggable pocket and new therapeutic strategies.

## Supporting Information Available

Supplementary methods; figures showing structures, binding affinity, cell viability, gene expression profiling, and cell cycle distrubution; tables sources of the analogues presented, cross-links identified, differentially expressed genes, and dimers; 1H-NMR, 13C-NMR, and HRMS spectra and HPLC traces of all target compounds (PDF)
Wild type AR LBD dimer in 1,000 MD simulations (MP4)
W751R mutant AR LBD dimer in 1,000 MD simulations (MP4)
F754V mutant AR LBD dimer in 1,000 MD simulations (MP4)
Modeled structure of the AR LBD bound with M17-B15 (PDB)

## Supplementary Material

oc2c01548_si_001.pdb

## Abbreviations

AR: androgen receptor
BLI: biolayer interferometry
CXMS: cross-linking coupled with mass spectrometry
DBD: DNA-binding domain
DEGs: differentially expressed genes
DHT: 5α-dihydrotestosteron
DIP: dimer interface pocket
*D*_max_: maximum end-to-end distance
DMSO: dimethyl sulfoxide
Enz: enzalutamide
FRET: fluorescence resonance energy transfer
GR: glucocorticoid receptor
LBD: ligand-binding domain
LBP: ligand binding pocket
MD: molecular dynamics
Mono: monomer
MR: mineralocorticoid receptor
NRs: nuclear receptors
NTD: N-terminal domain
PCa: prostate cancer
PR: progesterone receptor
PSA: prostate specific antigen
qPCR: quantitative PCR
RMSD: root-mean square deviation
SAR: structure–activity relationship
SAXS: small-angle X-ray scattering
SBVS: structure-based virtual screening
TMPRSS2: transmembrane protease serine 2

## References

[ref1] Kono M.; Fujii T.; Lim B.; Karuturi M. S.; Tripathy D.; Ueno N. T. Androgen receptor function and androgen receptor-targeted therapies in breast cancer: A Review. JAMA Oncol. 2017, 3 (9), 1266–1273. 10.1001/jamaoncol.2016.4975. 28301631

[ref2] McNamara K. M.; Moore N. L.; Hickey T. E.; Sasano H.; Tilley W. D. Complexities of androgen receptor signalling in breast cancer. Endocr. Relat. Cancer 2014, 21 (4), 161–181. 10.1530/ERC-14-0243. 24951107

[ref3] Watson P. A.; Arora V. K.; Sawyers C. L. Emerging mechanisms of resistance to androgen receptor inhibitors in prostate cancer. Nat. Rev. Cancer 2015, 15 (12), 701–711. 10.1038/nrc4016. 26563462 PMC4771416

[ref4] Sahu B.; Laakso M.; Ovaska K.; Mirtti T.; Lundin J.; Rannikko A.; Sankila A.; Turunen J. P.; Lundin M.; Konsti J.; et al. Dual role of FoxA1 in androgen receptor binding to chromatin, androgen signalling and prostate cancer. EMBO J. 2011, 30 (19), 3962–3976. 10.1038/emboj.2011.328. 21915096 PMC3209787

[ref5] Shang Y.; Myers M.; Brown M. Formation of the androgen receptor transcription complex. Mol. Cell 2002, 9 (3), 601–610. 10.1016/S1097-2765(02)00471-9. 11931767

[ref6] Grasso C. S.; Wu Y. M.; Robinson D. R.; Cao X.; Dhanasekaran S. M.; Khan A. P.; Quist M. J.; Jing X.; Lonigro R. J.; Brenner J. C.; et al. The mutational landscape of lethal castration-resistant prostate cancer. Nature 2012, 487 (7406), 239–243. 10.1038/nature11125. 22722839 PMC3396711

[ref7] Robinson D.; Van Allen E. M.; Wu Y. M.; Schultz N.; Lonigro R. J.; Mosquera J. M.; Montgomery B.; Taplin M. E.; Pritchard C. C.; Attard G.; et al. Integrative clinical genomics of advanced prostate cancer. Cell 2015, 162 (2), 454 10.1016/j.cell.2015.06.053. 28843286

[ref8] Hughes I. A.; Davies J. D.; Bunch T. I.; Pasterski V.; Mastroyannopoulou K.; MacDougall J. Androgen insensitivity syndrome. Lancet 2012, 380 (9851), 1419–1428. 10.1016/S0140-6736(12)60071-3. 22698698

[ref9] Boehmer A. L.; Brinkmann O.; Bruggenwirth H.; van Assendelft C.; Otten B. J.; Verleun-Mooijman M. C.; Niermeijer M. F.; Brunner H. G.; Rouwe C. W.; Waelkens J. J.; et al. Genotype versus phenotype in families with androgen insensitivity syndrome. J. Clin. Endocrinol. Metab. 2001, 86 (9), 4151–4160. 10.1210/jcem.86.9.7825. 11549642

[ref10] Tadokoro-Cuccaro R.; Hughes I. A. Androgen insensitivity syndrome. Curr. Opin. Endocrinol. Diabetes Obes. 2014, 21 (6), 499–503. 10.1097/MED.0000000000000107. 25354046

[ref11] Saranya B.; Bhavani G.; Arumugam B.; Jayashankar M.; Santhiya S. T. Three novel and two known androgen receptor gene mutations associated with androgen insensitivity syndrome in sex-reversed XY female patients. J. Genet. 2016, 95 (4), 911–921. 10.1007/s12041-016-0716-0. 27994190

[ref12] Gottlieb B.; Beitel L. K.; Nadarajah A.; Paliouras M.; Trifiro M. The androgen receptor gene mutations database: 2012 update. Hum. Mutat. 2012, 33 (5), 887–894. 10.1002/humu.22046. 22334387

[ref13] Nadal M.; Prekovic S.; Gallastegui N.; Helsen C.; Abella M.; Zielinska K.; Gay M.; Vilaseca M.; Taules M.; Houtsmuller A. B.; et al. Structure of the homodimeric androgen receptor ligand-binding domain. Nat. Commun. 2017, 8, 14388 10.1038/ncomms14388. 28165461 PMC5303882

[ref14] Hou T.; Li D.; Sheng R.; Fu W.; Hu C.; Yang H.; Zhang M.; Liao J. Thiadiazole amide derivatives and their applications. CN113444081B, 2021.

[ref15] Raviscioni M.; He Q.; Salicru E. M.; Smith C. L.; Lichtarge O. Evolutionary identification of a subtype specific functional site in the ligand binding domain of steroid receptors. Proteins 2006, 64 (4), 1046–1057. 10.1002/prot.21074. 16835908

[ref16] Tadokoro R.; Bunch T.; Schwabe J. W.; Hughes I. A.; Murphy J. C. Comparison of the molecular consequences of different mutations at residue 754 and 690 of the androgen receptor (AR) and androgen insensitivity syndrome (AIS) phenotype. Clin. Endocrinol. 2009, 71 (2), 253–260. 10.1111/j.1365-2265.2008.03462.x. 19178528

[ref17] Li D.; Zhou W.; Pang J.; Tang Q.; Zhong B.; Shen C.; Xiao L.; Hou T. A magic drug target: androgen receptor. Med. Res. Rev. 2019, 39 (5), 1485–1514. 10.1002/med.21558. 30569509

[ref18] Prensner J. R.; Rubin M. A.; Wei J. T.; Chinnaiyan A. M. Beyond PSA: the next generation of prostate cancer biomarkers. Sci. Transl. Med. 2012, 4 (127), 127rv123 10.1126/scitranslmed.3003180. PMC3799996 22461644

[ref19] Gong Z.; Ye S. X.; Tang C. Tightening the crosslinking distance restraints for better resolution of protein structure and dynamics. Structure 2020, 28 (10), 1160–1167. 10.1016/j.str.2020.07.010. 32763142

[ref20] Nelson W. G.; Yegnasubramanian S. Resistance emerges to second-generation antiandrogens in prostate cancer. Cancer Discovery 2013, 3 (9), 971–974. 10.1158/2159-8290.CD-13-0405. 24019330 PMC3800038

[ref21] Borgmann H.; Lallous N.; Ozistanbullu D.; Beraldi E.; Paul N.; Dalal K.; Fazli L.; Haferkamp A.; Lejeune P.; Cherkasov A.; et al. Moving towards precision urologic oncology: Targeting Enzalutamide-resistant prostate cancer and mutated forms of the androgen receptor using the novel inhibitor Darolutamide (ODM-201. Eur. Urol. 2018, 73 (1), 4–8. 10.1016/j.eururo.2017.08.012. 28851578

[ref22] Horning A. M.; Wang Y.; Lin C. K.; Louie A. D.; Jadhav R. R.; Hung C. N.; Wang C. M.; Lin C. L.; Kirma N. B.; Liss M. A.; et al. Single-cell RNA-seq reveals a subpopulation of prostate cancer cells with enhanced cell-cycle-related transcription and attenuated androgen response. Cancer Res. 2018, 78 (4), 853–864. 10.1158/0008-5472.CAN-17-1924. 29233929 PMC5983359

[ref23] He Y.; Hwang D. J.; Ponnusamy S.; Thiyagarajan T.; Mohler M. L.; Narayanan R.; Miller D. D. Pyrazol-1-yl-propanamides as SARD and pan-antagonists for the treatment of enzalutamide-resistant prostate cancer. J. Med. Chem. 2020, 63 (21), 12642–12665. 10.1021/acs.jmedchem.0c00943. 33095584 PMC7703681

[ref24] Ponnusamy S.; He Y.; Hwang D. J.; Thiyagarajan T.; Houtman R.; Bocharova V.; Sumpter B. G.; Fernandez E.; Johnson D.; Du Z.; et al. Orally bioavailable androgen receptor degrader, potential next-generation therapeutic for enzalutamide-resistant prostate cancer. Clin. Cancer Res. 2019, 25 (22), 6764–6780. 10.1158/1078-0432.CCR-19-1458. 31481513

[ref25] Ostrowski J.; Kuhns J. E.; Lupisella J. A.; Manfredi M. C.; Beehler B. C.; Krystek S. R. Jr.; Bi Y.; Sun C.; Seethala R.; Golla R.; et al. Pharmacological and x-ray structural characterization of a novel selective androgen receptor modulator: potent hyperanabolic stimulation of skeletal muscle with hypostimulation of prostate in rats. Endocrinology 2007, 148 (1), 4–12. 10.1210/en.2006-0843. 17008401

