# Supplementary material for: Small-Molecule Inhibition of Androgen Receptor Dimerization as a Strategy against Prostate Cancer
Source: ACS Cent Sci. 2023 Mar 8;9(4):675–84. doi: 10.1021/acscentsci.2c01548 (PMC10141604; doi:10.1021/acscentsci.2c01548)
Supplement: Supplementary file 5 — oc2c01548_si_005.pdf [file oc2c01548_si_005.pdf]

Supplementary Information

Small-Molecule Inhibition of AR Dimerization as a Strategy Against Prostate Cancer

Weitao Fu[+], Hao Yang[+], Chenxian Hu[+], Jianing Liao[+], Zhou Gong, Minkui Zhang, Shuai Yang, Shangxiang Ye, Yixuan Lei, Rong Sheng, Zhiguo Zhang, Xiaojun Yao, Chun Tang[*], Dan Li[*], Tingjun Hou[*]

[+] These authors contributed equally to this work

***Corresponding authors**

Tingjun Hou

E-mail: tingjunhou@zju.edu.cn

Dan Li

E-mail: lidancps@zju.edu.cn

Chun Tang

E-mail: Tang_Chun@pku.edu.cn

Table of Content

METHODS **S4**

Chemistry S4
Construction of the AR^{W751R} and AR^{F754V} Mutated Models S20
Molecular Dynamics (MD) Simulations for Dimers of AR^{WT}, AR^{W751R} and AR^{F754V} S21
SBVS Protocol S22
MD Simulations for AR LBD Monomer Bound with M17 .... S22
Cell Culture S23
AR Transcriptional Activity Assay S23
Competitive Ligand Binding Assay S24
Secreted Prostate-Specific Antigen (PSA) Assay S24
Cell Viability Assay S24
Protein Expression and Purification S24
BLI Assay S25
CXMS Analysis S26
Structure Modeling of AR LBD Dimer According to the CXMS results S27
Small-Angle X-ray Scattering (SAXS) Analysis S28
Luciferase Reporter Assays for FL-AR^{F876L} and FL- $AR^{F876L/T877A}$ S28
Acceptor Photobleaching Fluorescence Resonance Energy Transfer (FRET) Microscopy Assay S29
Cell Culture Sampling and Transcriptome Sequencing S30
Analysis of Sequences and Structures of Nuclear Receptor Ligand Binding Domains (NR LBDs) S30
Clonogenic Assay S31
Quantitative PCR (qPCR) for *PSA*, *CDC20*, *CENPF*, *MKI67* S31
Western Blot S31
Extraction of Nuclear and Cytoplasmic Fractions S32
Xenograft Studies S32
Luciferase Reporter Assays for PR, GR, and MR S33
Cell Cycle and Apoptosis for LNCaP cells S33
Statistical Analysis S34

Supplementary Figures **S35**

Figure S1. Structure of AR dimer (PDB code: 5JJM). S35
Figure S2. AIS-associated mutations of W751R and F754V inhibit AR LBD dimerization S36
Figure S3. M17 inhibited AR transcription without binding affinity to the LBP of AR LBD S37
Figure S4. Cell viability of M17-B15 against various of cells S38
Figure S5. Comparison of differentially expressed genes (DEGs) S39

Figure S6. Structural differences of AR DIP toward DIPs of PR, GR and MR S40
Figure S7. Cell viability of M17-B15 against various of cancer cells........................S41
Figure S8. M17-B15 showed no cell cycle progression arrest and apoptosis induction activities on LNCaP cells S42
Supplementary Tables S43
Table S1. The source of the 25 analogues of M17 ....S43
Table S2. Cross-links identified for the LBD of AR alone.......................................S44
Table S3. Cross-links identified for the AR LBD with M17-B15 ....S47
Table S4. Differentially expressed genes between DHT, Enz and M17-B15 ....S53
Table S5. Primers used in this study S54
Supplementary Movies S54
^{1}H-NMR, ^{13}C-NMR, HRMS spectra, HPLC Traces of All Target Compounds S55
Supplementary References S93

METHODS

Chemistry

Synthesis of M17 Analogues

Scheme 1. Synthesis of Compounds M17, M17-A2, M17-B1~M17-B4, M17-B10, M17-B12~M17-B18[a]

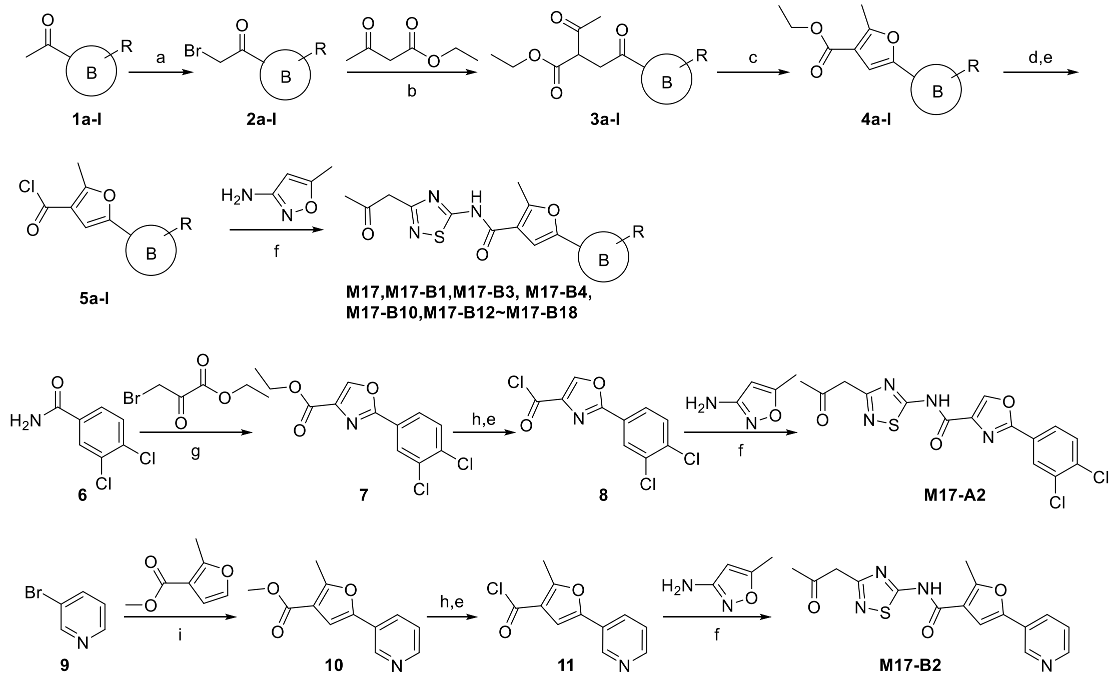

[a]Reagents and conditions: (a) NBS, TsOH, MeCN, 55 °C, 1 h; (b) NaH, anhydrous THF, 0-25 °C, overnight; (c) TfOH, MeCN, reflux, 1 h; (d) i. NaOH, H_2O, EtOH, rt, overnight; ii. HCl, H_2O; (e) $SOCl_2$, reflux, 2.5 h; (f) KSCN, MeCN, 60 °C, 1.5 h; (g) i. $NaHCO_3$, anhydrous THF, 60 °C, overnight; ii. TFAA, anhydrous THF, 0-25 °C, overnight; (h) LiOH, MeOH, H_2O, reflux, 1 h; (i) AcOK, $Pd(OAc)_2$, DMA, 150 °C, overnight.

Scheme 2. Synthesis of Compounds M17-B19~M17-B23[a]

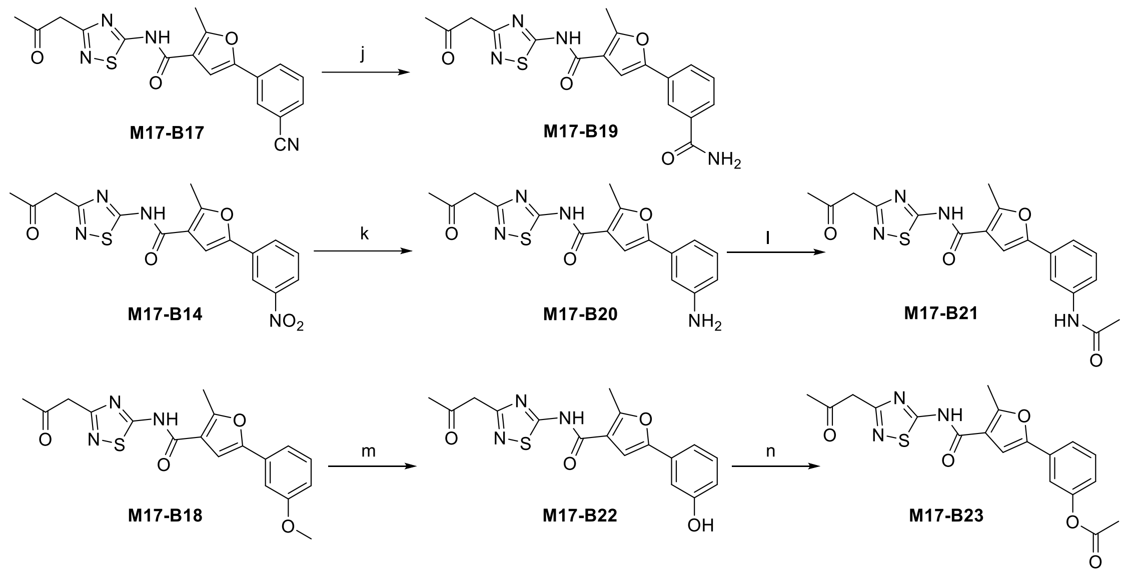

[a]Reagents and conditions: (j) 30% H_2O_2, NaOH, EtOH, 50 °C, overnight; (k) $SnCl_2{\cdot}H_2O$, EtOH, 70 °C, 5 h; (l) acetyl chloride, EtOH, MeCN, 0 °C-reflux, 6 h; (m) BBr_3, anhydrous DCM, -78-25 °C, overnight; (n) acetyl chloride, TEA, MeCN, 0-25 °C, 6 h.

General Methods and Materials

All the chemical reagents and reaction solvents were purchased commercially and of research grade or better. All the reactions were monitored by thin-layer chromatography (TLC) and then visualized with an UV lamp (254 nm). All final compounds were purified to ≥95% by HPLC with UV detection at 254 nm. NMR spectra were acquired on a Bruker AVANCE III 500 spectrometer with TMS as the internal standard in DMSO-*d*6 or $CDCl_3$. The chemical shifts are reported in parts per million (ppm), the coupling constants (*J*) are expressed in hertz (Hz). The HRMS were measured on an Agilent 6546 LC/Q-TOF or an Agilent 1290 HPLC-6224 Time of Fight Mass Spectrometer.

General Procedure for Synthesis of Compounds 3a-3l

To a stirring suspension of *N*-Bromosuccinimide (6.60 mmol) and *p*-Toluenesulfonic acid (3.00 mmol) in 20 mL MeCN was added acetophenone (**1a-1l**, 6.00 mmol). This solution was stirred at 55 °C for 1 h, then cooled to room temperature, and water (20 mL) was added. The mixture was extracted with EtOAc (3 × 30 mL), and the combined organic layer was washed with brine (2 × 50 mL), dried over anhydrous Na_2SO_4, and concentrated

to give a residue, which was purified by column-chromatograph (EtOAc/PE) to give compounds **2a-2l**.

To a suspension of sodium hydride (3.33 mmol) in 20 mL anhydrous THF was added ethyl acetoacetate (3.00 mmol) at 0 °C. This solution was stirred at 0 °C for 30 min and compound **2a-2l** (3.33 mmol) was added to the solution. After a few minutes, the solution was warmed to room temperature, stirred overnight and then quenched with water (15 mL). The mixture was extracted with EtOAc (3 × 30 mL), and the combined organic layer was washed with brine (2 × 50 mL), dried over anhydrous Na_2SO_4, and concentrated to give a residue, which was purified by column-chromatograph (EtOAc/PE) to give the desired product.

*Ethyl 2-acetyl-4-(3,4-dichlorophenyl)-4-oxobutanoate (**3a**)*

Yellow solid, yield: 82 %. ^{1}H NMR (500 MHz, $CDCl_3$): δ 8.06 (d, J = 2.0 Hz, 1H), 7.81 (dd, J_1 = 8.5 Hz, J_2 = 2.0 Hz, 1H), 7.56 (d, J = 8.0 Hz, 1H), 4.27-4.19 (m, 3H), 3.66 (dd, J_1 = 18.5 Hz, J_2 = 8.5 Hz, 1H), 3.44 (dd, J_1 = 18.5 Hz, J_2 = 5.5 Hz, 1H), 2.45 (s, 3H), 1.30 (t, J = 7.0 Hz, 3H). MS (ESI) m/z = 317.0 $[M+H]^+$.

*Ethyl 2-acetyl-4-oxo-4-phenylbutanoate (**3b**)*

Yellow solid, yield: 60 %. ^{1}H NMR (500 MHz, $CDCl_3$): δ 8.00-7.96 (m, 2H), 7.61-7.56 (m, 1H), 7.50-7.44 (m, 2H), 4.26-4.20 (m, 3H), 3.72 (dd, J_1 = 18.5 Hz, J_2 = 8.5 Hz, 1H), 3.53 (dd, J_1 = 18.5 Hz, J_2 = 5.5 Hz, 1H), 2.45 (s, 3H), 1.30 (t, J = 7.0 Hz, 3H). MS (ESI) m/z = 249.1 $[M+H]^+$.

*Ethyl 2-acetyl-4-(naphthalen-1-yl)-4-oxobutanoate (**3c**)*

White solid, yield: 83 %. ^{1}H NMR (500 MHz, $CDCl_3$): δ 8.58 (d, J = 8.5 Hz, 1H), 8.02-8.00 (m, 2H), 7.87 (d, J = 8.0 Hz, 1H), 7.60-7.56 (m, 1H), 7.54-7.50 (m, 2H), 4.32 (dd, J_1 = 8.0 Hz, J_2 = 6.0 Hz, 1H), 4.25 (dd, J_1 = 14.5 Hz, J_2 = 7.0 Hz, 2H), 3.79 (dd, J_1 = 18.0 Hz, J_2 = 8.0 Hz, 1H), 3.59 (dd, J_1 = 8.0 Hz, J_2 = 6.0 Hz, 1H), 2.48 (s, 3H), 1.30 (t, J = 7.0 Hz, 3H). MS (ESI) m/z: 299.1 $[M+H]^+$.

*Ethyl 2-acetyl-4-(naphthalen-2-yl)-4-oxobutanoate (**3d**)*

White solid, yield: 81 %. ^{1}H NMR (500 MHz, $CDCl_3$): δ 8.54 (s, 1H), 8.01 (dd, J_1 = 8.5 Hz, J_2 = 2.0 Hz, 1H), 7.98 (d, J = 8.0 Hz, 1H), 7.89 (t, J = 8.5 Hz, 2H), 7.63-7.60 (m, 1H), 7.58-7.55 (m, 1H), 4.29 (dd, J_1 = 8.0 Hz, J_2 = 6.0 Hz, 1H), 4.25 (dd, J_1 = 14.0 Hz, J_2 = 7.0 Hz, 2H), 3.87 (dd, J_1 = 18.0 Hz, J_2 = 8.0 Hz, 1H), 3.67 (dd, J_1 = 8.0 Hz, J_2 = 6.0 Hz, 1H), 2.48 (s, 3H), 1.31 (t, J = 7.0 Hz, 3H). MS (ESI) *m/z*: 299.1 $[M+H]^+$.

*Ethyl 2-acetyl-4-(3-chlorophenyl)-4-oxobutanoate (**3e**)*

Yellow solid, yield: 63 %. ^{1}H NMR (500 MHz, $CDCl_3$): δ 7.95 (t, J = 2.0 Hz, 1H), 7.88-7.84 (m, 1H), 7.58-7.53 (m, 1H), 7.42 (t, J = 8.0 Hz, 1H), 4.27-4.19 (m, 3H), 3.68 (dd, J_1 = 18.5 Hz, J_2 = 8.0 Hz, 1H), 3.51-3.44 (dd, J_1 = 18.5 Hz, J_2 = 5.5 Hz, 1H), 2.44 (s, 3H), 1.30 (t, J = 7.0 Hz, 3H). MS (ESI) *m/z* = 283.1 $[M+H]^+$.

*Ethyl 2-acetyl-4-(3-fluorophenyl)-4-oxobutanoate (**3f**)*

White solid, yield: 80 %. ^{1}H NMR (500 MHz, $CDCl_3$): δ 7.78 (t, J = 1.5 Hz, 1H), 7.66-7.64 (m, 1H), 7.48-7.44 (m, 1H), 7.31-7.27 (m, 1H), 4.27-4.20 (m, 3H), 3.69 (dd, J_1 = 18.5 Hz, J_2 = 8.0 Hz, 1H), 3.51-3.45 (m, 1H), 2.45 (s, 3H), 1.30 (t, J = 7.0 Hz, 3H). MS (ESI) *m/z*: 267.1 $[M+H]^+$.

*Ethyl 2-acetyl-4-(3-bromophenyl)-4-oxobutanoate (**3g**)*

Yellow solid, yield: 64 %. ^{1}H NMR (500 MHz, $CDCl_3$): δ 8.11 (t, J = 2.0 Hz, 1H), 7.93-7.89 (m, 1H), 7.73-7.69 (m, 1H), 7.36 (t, J = 8.0 Hz, 1H), 4.26-4.19 (m, 3H), 3.68 (dd, J_1 = 18.5 Hz, J_2 = 8.0 Hz, 1H), 3.47 (dd, J_1 = 18.5 Hz, J_2 = 5.5 Hz, 1H), 2.44 (s, 3H), 1.30 (t, J = 7.0 Hz, 3H). MS (ESI) *m/z* = 327.0 $[M+H]^+$.

*Ethyl 2-acetyl-4-(3-nitrophenyl)-4-oxobutanoate (**3h**)*

Yellow solid, yield: 67 %. ^{1}H NMR (500 MHz, $CDCl_3$): δ 8.81 (t, J = 2.0 Hz, 1H), 8.47-8.43 (m, 1H), 8.33-8.29 (m, 1H), 7.70 (t, J = 8.0 Hz, 1H), 4.29-4.22 (m, 3H), 3.76 (dd, J_1

= 18.5 Hz, J_2 = 8.0 Hz, 1H), 3.53 (dd, J_1 = 18.5 Hz, J_2 = 5.5 Hz, 1H), 2.46 (s, 3H), 1.32 (t, *J* = 7.0 Hz, 3H). MS (ESI) *m/z* = 294.1 $[M+H]^+$.

*Ethyl 2-acetyl-4-oxo-4-(3-(trifluoromethyl)phenyl)butanoate (**3i**)*

Yellow solid, yield: 71 %. ^{1}H NMR (500 MHz, $CDCl_3$): δ 8.23 (s, 1H), 8.17 (d, *J* = 7.5 Hz, 1H), 7.85 (d, *J* = 8.0 Hz, 1H), 7.63 (t, *J* = 7.5 Hz, 1H), 4.28-4.21 (m, 3H), 3.74 (dd, J_1 = 18.5 Hz, J_2 = 8.5 Hz, 1H), 3.52 (dd, J_1 = 18.5 Hz, J_2 = 5.5 Hz, 1H), 2.46 (s, 3H), 1.31 (t, *J* = 7.0 Hz, 3H). MS (ESI) *m/z* = 317.1 $[M+H]^+$.

*Ethyl 2-acetyl-4-(3-(tert-butyl)phenyl)-4-oxobutanoate (**3j**)*

Yellow oil, yield: 80 %. ^{1}H NMR (500 MHz, DMSO-d_6): δ 7.85-7.83 (m, 1H), 7.81-7.78 (m, 1H), 7.41-7.38 (m, 1H), 7.33 (t, *J* = 7.5 Hz, 1H), 4.19-4.16 (m, 2H), 4.05 (t, *J* = 9.0 Hz, 1H), 3.50-3.46 (m, 2H), 2.33 (s, 3H), 1.33 (s, 9H), 1.24 (t, *J* = 7.0 Hz, 3H). MS (ESI) *m/z*: 305.2 $[M+H]^+$.

*Ethyl 2-acetyl-4-(3-cyanophenyl)-4-oxobutanoate (**3k**)*

White solid, yield: 82 %. ^{1}H NMR (500 MHz, $CDCl_3$): δ 8.27 (t, *J* = 1.5 Hz, 1H), 8.23-8.18 (m, 1H), 7.88-7.84 (m, 1H), 7.63 (t, *J* = 3.0 Hz, 1H), 4.27-4.22 (m, 3H), 3.70 (dd, J_1 = 18.0 Hz, J_2 = 8.0 Hz, 1H), 3.47 (dd, J_1 = 18.5 Hz, J_2 = 5.5 Hz, 1H), 2.45 (s, 3H), 1.31 (t, *J* = 7.0 Hz, 3H). MS (ESI) *m/z*: 274.1 $[M+H]^+$.

*Ethyl 2-acetyl-4-(3-methoxyphenyl)-4-oxobutanoate (**3l**)*

Yellow solid, yield: 65 %. ^{1}H NMR (500 MHz, $CDCl_3$): δ 7.60-7.56 (m, 1H), 7.48 (dd, J_1 = 3.0 Hz, J_2 = 1.5 Hz, 1H), 7.38 (t, *J* = 8.0 Hz, 1H), 7.15-7.11 (m, 1H), 4.26-4.19 (m, 3H), 3.85 (s, 3H), 3.74-3.67 (m, 1H), 3.51 (dd, J_1 = 18.5 Hz, J_2 = 5.5 Hz, 1H), 2.45 (s, 3H), 1.30 (t, *J* = 7.0 Hz, 3H). MS (ESI) *m/z* = 279.1 $[M+H]^+$.

General Procedure for Synthesis of Compounds 4a-4l

To a solution of compound **3a-3l** (2.50 mmol) in 15 mL MeCN was added trifluoromethanesulfonic acid (2.50 mmol) under a nitrogen atmosphere. This solution was heated to reflux for 1 h, then cooled to room temperature, and water (20 mL) was added. The mixture was extracted with EtOAc (3 × 30 mL), and the combined organic layer was washed with brine (2 × 50 mL), dried over anhydrous Na_2SO_4, and concentrated to give a residue, which was purified by column-chromatograph (EtOAc/PE) to give the desired product.

*Ethyl 5-(3,4-dichlorophenyl)-2-methylfuran-3-carboxylate (**4a**)*

Yellow solid, yield: 81 %. ^{1}H NMR (500 MHz, $CDCl_3$): δ 7.72 (t, *J* = 1.0 Hz, 1H), 7.46-7.43 (m, 2H), 6.92 (s, 1H), 4.32 (q, *J* = 7.0 Hz, 2H), 2.65 (s, 3H), 1.37 (t, *J* = 7.0 Hz, 3H). MS (ESI) *m/z* = 299.0 $[M+H]^+$.

*Ethyl 2-methyl-5-phenylfuran-3-carboxylate (**4b**)*

Yellow solid, yield: 96 %. ^{1}H NMR (500 MHz, $CDCl_3$): δ 7.66-7.62 (m, 2H), 7.41-7.36 (m, 2H), 7.29-7.25 (m, 1H), 6.88 (s, 1H), 4.31 (q, *J* = 7.0 Hz, 2H), 2.65 (s, 3H), 1.37 (t, *J* = 7.0 Hz, 3H). MS (ESI) *m/z* = 231.1 $[M+H]^+$.

*Ethyl 2-methyl-5-(naphthalen-1-yl)furan-3-carboxylate (**4c**)*

White solid, yield: 81 %. ^{1}H NMR (500 MHz, DMSO-d_6): δ 8.28 (d, *J* = 9.0 Hz, 1H), 7.89-7.84 (m, 2H), 7.68 (d, *J* = 7.5 Hz, 1H), 7.55-7.47 (m, 3H), 6.91 (s, 1H), 4.26 (q, *J* = 7.0 Hz, 2H), 2.65 (s, 3H), 1.33 (t, *J* = 7.0 Hz, 3H). MS (ESI) *m/z*: 281.1 $[M+H]^+$.

*Ethyl 2-methyl-5-(naphthalen-2-yl)furan-3-carboxylate (**4d**)*

White solid, yield: 85 %. ^{1}H NMR (500 MHz, DMSO-d_6): δ 8.11 (s, 1H), 7.87-7.80 (m, 3H), 7.72 (dd, J_1 = 8.5 Hz, J_2 = 2.0 Hz, 1H), 7.51-7.44 (m, 2H), 7.00 (s, 1H), 4.33 (q, *J* = 7.0 Hz, 2H), 2.70 (s, 3H), 1.39 (t, *J* = 7.0 Hz, 3H). MS (ESI) *m/z*: 281.1 $[M+H]^+$.

*Ethyl 5-(3-chlorophenyl)-2-methylfuran-3-carboxylate (**4e**)*

Yellow solid, yield: 87 %. ^{1}H NMR (500 MHz, $CDCl_3$): δ 7.62 (t, *J* = 2.0 Hz, 1H), 7.52-7.48 (m, 1H), 7.30 (t, *J* = 8.0 Hz, 1H), 7.25-7.21 (m, 1H), 6.91 (s, 1H), 4.31 (q, *J* = 7.0 Hz, 2H), 2.65 (s, 3H), 1.37 (t, *J* = 7.0 Hz, 3H). MS (ESI) *m/z* = 265.1 $[M+H]^+$.

*Ethyl 5-(3-fluorophenyl)-2-methylfuran-3-carboxylate (**4f**)*

White solid, yield: 75 %. ^{1}H NMR (500 MHz, $CDCl_3$): δ 7.41 (t, *J* = 1.5 Hz, 1H), 7.36-7.32 (m, 2H), 6.98-6.94 (m, 1H), 6.91 (s, 1H), 4.32 (q, *J* = 7.0 Hz, 2H), 2.65 (s, 3H), 1.38 (t, *J* = 7.0 Hz, 3H). MS (ESI) *m/z*: 249.1 $[M+H]^+$.

*Ethyl 5-(3-bromophenyl)-2-methylfuran-3-carboxylate (**4g**)*

Yellow solid, yield: 70 %. ^{1}H NMR (500 MHz, $CDCl_3$): δ 7.79 (t, *J* = 2.0 Hz, 1H), 7.57-7.53 (m, 1H), 7.41-7.37 (m, 1H), 7.24 (t, *J* = 8.0 Hz, 1H), 6.91 (s, 1H), 4.32 (q, *J* = 7.0 Hz, 2H), 2.65 (s, 3H), 1.37 (t, *J* = 7.0 Hz, 3H). MS (ESI) *m/z* = 309.0 $[M+H]^+$.

*Ethyl 2-methyl-5-(3-nitrophenyl)furan-3-carboxylate (**4h**)*

Yellow solid, yield: 62 %. ^{1}H NMR (500 MHz, $CDCl_3$): δ 8.48 (t, *J* = 2.0 Hz, 1H), 8.13-8.09 (m, 1H), 7.95-7.91 (m, 1H), 7.56 (t, *J* = 8.0 Hz, 1H), 7.06 (s, 1H), 4.33 (q, *J* = 7.0 Hz, 2H), 2.69 (s, 3H), 1.39 (t, *J* = 7.0 Hz, 3H). MS (ESI) *m/z* = 276.1 $[M+H]^+$.

*Ethyl 2-methyl-5-(3-(trifluoromethyl)phenyl)furan-3-carboxylate (**4i**)*

Yellow solid, yield: 78 %. ^{1}H NMR (500 MHz, $CDCl_3$): δ 7.90-7.87 (m, 1H), 7.82-7.78 (m, 1H), 7.53-7.48 (m, 2H), 6.99 (s, 1H), 4.33 (q, *J* = 7.0 Hz, 2H), 2.67 (s, 3H), 1.38 (t, *J* = 7.0 Hz, 3H). MS (ESI) *m/z* = 299.1 $[M+H]^+$.

*Ethyl 5-(3-(tert-butyl)phenyl)-2-methylfuran-3-carboxylate (**4j**)*

Yellow oil, yield: 75 %. ^{1}H NMR (500 MHz, $CDCl_3$): δ 7.73-7.72 (m, 1H), 7.54-7.52 (m, 1H), 7.36-7.34 (m, 2H), 7.18 (s, 1H), 4.26 (q, *J* = 7.0 Hz, 2H), 2.62 (s, 3H), 1.32-1.29 (m, 12H). MS (ESI) *m/z*: 287.2 $[M+H]^+$.

Ethyl 5-(3-cyanophenyl)-2-methylfuran-3-carboxylate (***4k***)

White solid, yield: 75 %. ^{1}H NMR (500 MHz, $CDCl_3$): δ 7.92 (t, *J* = 1.5 Hz, 1H), 7.85-7.81 (m, 1H), 7.56-7.52 (m, 1H), 7.49 (t, *J* = 3.0 Hz, 1H), 6.99 (s, 1H), 4.33 (q, *J* = 7.0 Hz, 2H), 2.67 (s, 3H), 1.38 (t, *J* = 7.0 Hz, 3H). MS (ESI) *m/z*: 256.1 $[M+H]^+$.

Ethyl 5-(3-methoxyphenyl)-2-methylfuran-3-carboxylate (***4l***)

Yellow solid, yield: 95 %. ^{1}H NMR (500 MHz, $CDCl_3$): δ 7.29 (t, *J* = 8.0 Hz, 1H), 7.25-7.21 (m, 1H), 7.19-7.16 (m, 1H), 6.88 (s, 1H), 6.84-6.80 (m, 1H), 4.31 (q, *J* = 7.0 Hz, 2H), 3.85 (s, 3H), 2.65 (s, 3H), 1.37 (t, *J* = 7.0 Hz, 3H). MS (ESI) *m/z* = 261.1 $[M+H]^+$.

General Procedure for Synthesis of Compounds M17, M17-B1, M17-B3, M17-B4, M17-B10, M17-B12~M17-B18

A solution of compound **4a-4l** (1.75 mmol) and sodium hydroxide (11.00 mmol) in EtOH/H_2O (3:1, 12 mL) was stirred overnight at room temperature, then ethanol was removed and the residue was acidified with 2.0 mol/L HCl to pH = 1. The mixture was extracted with EtOAc (3 × 20 mL) and the combined organic layer was washed with brine (2 × 50 mL), dried over anhydrous Na_2SO_4, and concentrated to give the 2-methyl-5-phenylfuran-3-carboxylic acids. A mixture of 2-methyl-5-phenylfuran-3-carboxylic acids (1.50 mmol) with 8 mL $SOCl_2$ was heated to reflux for 2.5 h, and then concentrated to dryness to get 2-methyl-5-phenylfuran-3-carbonyl chlorides (**5a-5l**).

A suspension of compound **5a-5l** (1.50 mmol) and potassium thiocyanate (1.65 mmol) in 10 mL MeCN was stirred at 60 °C for 30 min, and then 5-methylisoxazol-3-amine (1.50 mmol) was added and stirred for another 1 h. After cooling to room temperature, water (20 mL) was added to the mixture and was extracted with EtOAc (3 × 30 mL). The combined organic layer was washed with brine (2 × 50 mL), dried over anhydrous Na_2SO_4, and concentrated to give a residue, which was purified by column-chromatograph (EtOAc/PE) to give the desired product.

5-(3,4-Dichlorophenyl)-2-methyl-N-(3-(2-oxopropyl)-1,2,4-thiadiazol-5-yl)furan-3-carboxamide (***M17***)

Yellow solid, yield: 63 %. ^{1}H NMR (500 MHz, DMSO-d_6): δ 13.18 (s, 1H), 7.82 (d, J = 2.0 Hz, 1H), 7.79-7.74 (m, 2H), 7.59 (dd, J_1 = 8.5 Hz, J_2 = 2.0 Hz, 1H), 4.04 (s, 2H), 2.70 (s, 3H), 2.21 (s, 3H). ^{13}C NMR (125 MHz, DMSO-d_6): δ 204.06, 175.90, 165.34, 162.62, 161.04, 149.06, 132.44, 131.92, 130.71, 130.09, 125.19, 123.76, 115.91, 106.86, 47.85, 30.22, 14.34. HRMS (ESI) *m/z* Calcd. for $C_{17}H_{14}Cl_2N_3O_3S^+$ [M+H]$^+$ 410.0127, Found 410.0120. Purity: 96.2 %.

2-Methyl-N-(3-(2-oxopropyl)-1,2,4-thiadiazol-5-yl)-5-phenylfuran-3-carboxamide (***M17-B1***)

Yellow solid, yield: 60 %. ^{1}H NMR (500 MHz, DMSO-d_6): δ 13.16 (s, 1H), 7.68 (s, 1H), 7.66-7.62 (m, 2H), 7.49 (t, J = 8.0 Hz, 2H), 7.39-7.33 (m, 1H), 4.04 (s, 2H), 2.71 (s, 3H), 2.21 (s, 3H). ^{13}C NMR (125 MHz, DMSO-d_6): δ 204.09, 175.99, 165.33, 162.93, 160.30, 151.61, 129.67, 129.62, 128.57, 123.76, 115.62, 104.80, 47.87, 30.23, 14.35. HRMS (ESI) *m/z* Calcd. for $C_{17}H_{15}N_3NaO_3S^+$ [M+Na]$^+$ 364.0726, Found 364.0728. Purity: 96.6 %.

2-Methyl-5-(naphthalen-1-yl)-N-(3-(2-oxopropyl)-1,2,4-thiadiazol-5-yl)furan-3-carboxamide (***M17-B3***)

White solid, yield: 70 %. ^{1}H NMR (500 MHz, DMSO-d_6): δ 13.25 (s, 1H), 8.44 (t, J = 1.5 Hz, 1H), 8.04 (d, J = 8.0 Hz, 1H), 8.00 (d, J = 8.0 Hz, 1H), 7.82-7.78 (m, 2H), 7.69-7.66 (m, 1H), 7.64-7.60 (m, 2H), 4.05 (s, 2H), 2.76 (s, 3H), 2.22 (s, 3H). ^{13}C NMR (125 MHz, DMSO-d_6): δ 204.07, 176.03, 165.35, 162.94, 160.61, 150.49, 134.07, 129.52, 129.48, 129.28, 127.65, 126.91, 126.78, 126.18, 126.01, 125.01, 115.44, 108.96, 47.89, 30.22, 14.33. HRMS (ESI) *m/z* Calcd. for $C_{21}H_{17}N_3NaO_3S^+$ [M+Na]$^+$ 414.0883, Found 414.0881. Purity: 95.1 %.

2-Methyl-5-(naphthalen-2-yl)-N-(3-(2-oxopropyl)-1,2,4-thiadiazol-5-yl)furan-3-carboxamide (***M17-B4***)

White solid, yield: 76 %. ^{1}H NMR (500 MHz, DMSO-d_6): δ 13.17 (s, 1H), 8.17 (s, 1H), 8.02-8.00 (m, 2H), 7.93 (d, J = 8.0 Hz, 1H), 7.80 (s, 1H), 7.73 (d, J = 8.5 Hz, 1H), 7.57-7.51

(m, 2H), 4.05 (s, 2H), 2.74 (s, 3H), 2.22 (s, 3H). ^{13}C NMR (125 MHz, DMSO-d_6): δ204.09, 175.98, 165.34, 162.88, 160.60, 151.66, 133.47, 132.87, 129.36, 128.62, 128.19, 127.39, 127.01, 126.92, 122.04, 115.78, 105.56, 47.88, 30.23, 14.40. HRMS (ESI) *m/z* Calcd. for $C_{21}H_{17}N_3NaO_3S^+$ [M+Na]$^+$ 414.0883, Found 414.0882. Purity: 97.2 %.

5-(3-Chlorophenyl)-2-methyl-N-(3-(2-oxopropyl)-1,2,4-thiadiazol-5-yl)furan-3-carboxamide (***M17-B10***)

Yellow solid, yield: 55 %. ^{1}H NMR (500 MHz, $CDCl_3$): δ 7.65 (t, *J* = 2.0 Hz, 1H), 7.54-7.50 (m, 1H), 7.36 (t, *J* = 8.0 Hz, 1H), 7.32-7.28(m, 1H), 6.88 (s, 1H), 4.02 (s, 2H), 2.77 (s, 3H), 2.28 (s, 3H). ^{13}C NMR (125 MHz, DMSO-d_6): δ 204.08, 175.95, 165.36, 162.76, 160.84, 149.97, 134.38, 131.64, 131.60, 128.25, 123.27, 122.33, 115.82, 106.33, 47.86, 30.23, 14.34. HRMS (ESI) *m/z* Calcd. for $C_{17}H_{15}ClN_3O_3S^+$ [M+H]$^+$ 376.0517, Found 376.0515. Purity: 95.2 %.

5-(3-Fluorophenyl)-2-methyl-N-(3-(2-oxopropyl)-1,2,4-thiadiazol-5-yl)furan-3-carboxamide (***M17-B12***)

White solid, yield: 77 %. ^{1}H NMR (500 MHz, DMSO-d_6): δ 13.15 (s, 1H), 7.73 (s, 1H), 7.55-7.51 (m, 1H), 7.47-7.45 (m, 1H), 7.40-7.35 (m, 1H), 7.22-7.17 (m, 1H), 4.04 (s, 2H), 2.70 (s, 3H), 2.21 (s, 3H). ^{13}C NMR (125 MHz, DMSO-d_6): δ 204.06, 175.94, 165.35, 163.02 (d, *J* = 241.2 Hz), 162.77, 160.72, 150.26 (d, *J* = 3.0 Hz), 131.89, 131.82, 119.88 (*J* = 2.6 Hz), 115.78, 115.25 (d, *J* = 21.1 Hz), 110.37 (d, *J* = 23.5 Hz), 106.21, 47.86, 30.21, 14.34. HRMS (ESI) *m/z* Calcd. for $C_{17}H_{15}FN_3O_3S^+$ [M+H]$^+$ 360.0813, Found 360.0811. Purity: 96.2 %.

5-(3-Bromophenyl)-2-methyl-N-(3-(2-oxopropyl)-1,2,4-thiadiazol-5-yl)furan-3-carboxamide (***M17-B13***)

Yellow solid, yield: 51 %. ^{1}H NMR (500 MHz, DMSO-d_6): δ 13.16 (s, 1H), 7.79-7.76 (m, 2H), 7.64-7.61 (m, 1H), 7.57-7.53 (m, 1H), 7.45 (t, *J* = 8.0 Hz, 1H), 4.04 (s, 2H), 2.70 (s, 3H), 2.21 (s, 3H). ^{13}C NMR (125 MHz, DMSO-d_6): δ 204.09, 175.93, 165.38, 162.78,

160.88, 149.84, 131.89, 131.82, 131.15, 126.13, 122.90, 122.69, 115.81, 106.39, 47.86, 30.24, 14.35. HRMS (ESI) *m/z* Calcd. for $C_{17}H_{15}BrN_3O_3S^+$ $[M+H]^+$ 420.0012, Found 420.0009. Purity: 96.0 %.

2-Methyl-5-(3-nitrophenyl)-N-(3-(2-oxopropyl)-1,2,4-thiadiazol-5-yl)furan-3-carboxamide (***M17-B14***)

Yellow solid, yield: 74 %. ^{1}H NMR (500 MHz, DMSO-*d*$_6$): δ 13.13 (s, 1H), 8.32 (t, *J* = 2.0 Hz, 1H), 8.18-8.14 (m, 1H), 8.03-7.99 (m, 1H), 7.89 (s, 1H), 7.76 (t, *J* = 8.0 Hz, 1H), 4.04 (s, 2H), 2.72 (s, 3H), 2.21 (s, 3H). ^{13}C NMR (125 MHz, DMSO-*d*$_6$): δ 204.07, 175.89, 165.38, 162.59, 161.36, 149.27, 148.80, 131.43, 131.01, 129.62, 122.83, 117.93, 115.93, 107.29, 47.85, 30.23, 14.35. HRMS (ESI) *m/z* Calcd. for $C_{17}H_{14}N_4NaO_5S^+$ $[M+Na]^+$ 409.0577, Found 409.0576. Purity: 97.4 %.

2-Methyl-N-(3-(2-oxopropyl)-1,2,4-thiadiazol-5-yl)-5-(3(trifluoromethyl)phenyl)-furan-3-carboxamide (***M17-B15***)

Yellow solid, yield: 71 %. ^{1}H NMR (500 MHz, $CDCl_3$): δ 7.92-7.89 (m, 1H), 7.83-7.79 (m, 1H), 7.60-7.53 (m, 2H), 6.96 (s, 1H), 4.02 (s, 2H), 2.79 (s, 3H), 2.28 (s, 3H). ^{13}C NMR (125 MHz, $CDCl_3$): δ 203.91, 175.18, 164.28, 161.35, 161.03, 151.41, 131.52 (q, *J* = 32.6 Hz), 129.99, 129.52, 126.89, 124.84 (q, *J* = 3.8 Hz), 123.85 (q, *J* = 272.9 Hz), 120.61 (q, *J* = 3.8 Hz), 114.94, 103.39, 47.87, 29.94, 14.17. HRMS (ESI) *m/z* Calcd. for $C_{18}H_{15}F_3N_3O_3S^+$ $[M+H]^+$ 410.0781, Found 410.0777. Purity: 97.8 %.

5-(3-(Tert-butyl)phenyl)-2-methyl-N-(3-(2-oxopropyl)-1,2,4-thiadiazol-5-yl)furan-3-carboxamide (***M17-B16***)

Yellow solid, yield: 86 %. ^{1}H NMR (500 MHz, DMSO-*d*$_6$): δ 13.11 (s, 1H), 7.72 (s, 1H), 7.65-7.63 (m, 1H), 7.47-7.44 (m, 1H), 7.41-7.40 (m, 2H), 4.04 (s, 2H), 2.71 (s, 3H), 2.21 (s, 3H), 1.34 (s, 9H). ^{13}C NMR (125 MHz, DMSO-*d*$_6$): δ204.06, 175.98, 165.36, 162.93, 160.21, 152.08, 151.92, 129.44, 129.41, 125.73, 121.06, 120.40, 115.52, 104.68, 47.88, 34.95, 31.47, 30.22, 14.34. HRMS (ESI) *m/z* Calcd. for $C_{21}H_{24}N_3O_3S^+$ $[M+H]^+$ 398.1533, Found 398.1514. Purity: 96.8 %.

5-(3-Cyanophenyl)-2-methyl-N-(3-(2-oxopropyl)-1,2,4-thiadiazol-5-yl)furan-3-carboxamide (***M17-B17***)

White solid, yield: 76 %. ^{1}H NMR (500 MHz, DMSO-d_6): δ 13.17 (s, 1H), 7.99 (t, *J* = 1.5 Hz, 1H), 7.66-7.62 (m, 1H), 7.81-7.78 (m, 2H), 7.68 (t, *J* = 8.0 Hz, 1H), 4.04 (s, 2H), 2.70 (s, 3H), 2.21 (s, 3H). ^{13}C NMR (125 MHz, DMSO-d_6): δ 204.06, 175.92, 165.36, 162.69, 161.08, 149.43, 131.84, 130.95, 130.73, 128.11, 126.99, 118.82, 115.93, 112.81, 106.92, 47.85, 30.22, 14.36. HRMS (ESI) *m/z* Calcd. for $C_{18}H_{15}N_4O_3S^+$ $[M+H]^+$ 367.0859, Found 367.0858. Purity: 95.3 %.

5-(3-Methoxyphenyl)-2-methyl-N-(3-(2-oxopropyl)-1,2,4-thiadiazol-5-yl)furan-3-carboxamide (***M17-B18***)

Yellow solid, yield: 63 %. ^{1}H NMR (500 MHz, DMSO-d_6): δ 13.14 (s, 1H), 7.70 (s, 1H), 7.40 (t, *J* = 8.0 Hz, 1H), 7.24-7.20 (m, 1H), 7.16-7.13 (m, 1H), 6.96-6.92 (m, 1H), 4.04 (s, 2H), 3.82 (s, 3H), 2.70 (s, 3H), 2.21 (s, 3H). ^{13}C NMR (125 MHz, DMSO-d_6): δ 204.09, 175.97, 165.36, 162.89, 160.32, 160.16, 151.44, 130.95, 130.86, 116.17, 115.60, 114.38, 108.88, 105.23, 55.61, 47.87, 30.23, 14.35. HRMS (ESI) *m/z* Calcd. for $C_{18}H_{18}N_3O_4S^+$ $[M+H]^+$ 372.1013, Found 372.1012. Purity: 95.4 %.

Synthesis of 2-(3,4-dichlorophenyl)-*N*-(3-(2-oxopropyl)-1,2,4-thiadiazol-5-yl)oxazole-4-carboxamide (M17-A2)

*Step 1. ethyl 2-(3,4-dichlorophenyl)oxazole-4-carboxylate (**7**)*

To a suspension of 3,4-dichlorobenzamide (300 mg, 1.58 mmol) and sodium bicarbonate (637 mg, 7.58 mmol) in anhydrous THF (15 mL) was added ethyl 3-bromo-2-oxopropanoate (298 μL, 2.37 mmol). This mixture was refluxed overnight and the resulting solid impurities were removed by filtration. The filtrate was concentrated to a residue, which was dissolved in anhydrous THF (10 mL), after cooling to 0°C, trifluoroacetic anhydride (263 μL, 1.89 mmol) was added. The solution was then warmed at room temperature and stirred overnight. The reaction mixture was slowly quenched with saturated aqueous $NaHCO_3$ (10 mL) and extracted with EtOAc (3 × 20 mL). The

combined organic layer was washed with brine (2 × 30 mL), dried over anhydrous Na_2SO_4, and concentrated to give a residue, which was purified by column-chromatograph (EtOAc/PE) to give compound **7**.

Yellow solid. Yield: 35 %. ^{1}H NMR (500 MHz, $CDCl_3$): δ 8.29 (s, 1H), 8.24 (d, J = 2.0 Hz, 1H), 7.95 (dd, J_1 = 8.5 Hz, J_2 = 2.0 Hz, 1H), 7.57 (d, J = 8.0 Hz, 1H), 4.44 (q, J = 7.0 Hz, 2H), 1.42 (t, J = 7.0 Hz, 3H). MS (ESI) m/z = 286.0 $[M+H]^+$.

*Step 2. 2-(3,4-dichlorophenyl)-N-(3-(2-oxopropyl)-1,2,4-thiadiazol-5-yl)oxazole-4-carboxamide (**M17-A2**)*

A solution of compound **7** (180 mg, 0.63 mmol) and lithium hydroxide (48 mg, 2.00 mmol) in $MeOH/H_2O$ (3:1, 4 mL) was heated to reflux and stirred for 1 h. The reaction mixture was cooled to room temperature, and concentrated under reduced pressure to get a residue, which was acidified with 2.0 mol/L HCl to pH = 1. The mixture was extracted with EtOAc (3 × 10 mL) and the combined organic layer was washed with brine (2 × 10 mL), dried over anhydrous Na_2SO_4, and concentrated to give 2-(3,4-dichlorophenyl)oxazole-4-carboxylic acid. Then, 5 mL $SOCl_2$ was added and heated to reflux for 2.5 h. The solution was concentrated to dryness to achieve 2-(3,4-dichlorophenyl)oxazole-4-carbonyl chloride (**8**).

A suspension of compound **8** (92 mg, 0.33 mmol) and potassium thiocyanate (36 mg, 0.37 mmol) in MeCN (8 mL) was stirred at 60 °C for 30 min, then 5-methylisoxazol-3-amine (33 mg, 0.33 mmol) was added and stirred for another 1 h. After cooling to room temperature, water (20 mL) was added. The mixture was extracted with EtOAc (3 × 20 mL), and the combined organic layer was washed with brine (2 × 30 mL), dried over anhydrous Na_2SO_4, and concentrated to give a residue, which was purified by column-chromatograph (EtOAc/PE) to give compound **M17-A2**.

Yellow solid. Yield: 55 %. ^{1}H NMR (500 MHz, $CDCl_3$): δ 8.48 (s, 1H), 8.16 (d, J = 2.0 Hz, 1H), 7.89 (dd, J_1 = 8.5 Hz, J_2 = 2.0 Hz, 1H), 7.62 (d, J = 8.5 Hz, 1H), 4.04 (s, 2H), 2.29 (s, 3H). ^{13}C NMR (125 MHz, $CDCl_3$): δ 203.12, 174.37, 164.87, 160.35, 158.57,

143.49, 136.37, 134.86, 133.84, 131.37, 128.66, 125.79, 125.52, 47.83, 29.92. HRMS (ESI) *m/z* Calcd. for $C_{15}H_{11}Cl_2N_4O_3S^+$ $[M+H]^+$ 396.9923, Found 396.9914. Purity: 97.8 %.

Synthesis of 2-methyl-*N*-(3-(2-oxopropyl)-1,2,4-thiadiazol-5-yl)-5-(pyridin-3-yl)furan-3-carboxamide (M17-B2)

*Step 1. methyl 2-methyl-5-(pyridin-3-yl)furan-3-carboxylate (**10**)*

A suspension of 3-bromopyridine (122 μL, 1.27 mmol), methyl 2-methylfuran-3-carboxylate (317 μL, 2.53 mmol), potassium acetate (248 mg, 2.53 mmol) and palladium diacetate (0.1 % equiv) in DMA (5 mL) was heated to 150 °C and stirred overnight. The reaction mixture was cooled to room temperature, and then water (20 mL) was added. The mixture was extracted with EtOAc (3 × 10 mL). The combined organic layer was washed with water (3× 50 mL) and then brine (2 × 50 mL), dried over anhydrous Na_2SO_4, and concentrated to give a residue, which was purified by column-chromatograph (EtOAc/PE) to give compound **10**.

Yellow solid. Yield: 73 %. ^{1}H NMR (500 MHz, $CDCl_3$): δ 8.92-8.89 (m, 1H), 8.51 (dd, J_1 = 5.0 Hz, J_2 = 1.5 Hz, 1H), 7.93-7.89 (m, 1H), 7.35-7.30 (m, 1H), 6.98 (s, 1H), 3.87 (s, 3H), 2.67 (s, 3H). MS (ESI) *m/z* = 218.1 $[M+H]^+$.

Step 2. 2-methyl-N-(3-(2-oxopropyl)-1,2,4-thiadiazol-5-yl)-5-(pyridin-3-yl)furan-3-carboxamide (M17-B2)

This step referred to the synthesis step 2 of compound **M17-A2**.

Yellow solid, yield: 89 %. ^{1}H NMR (500 MHz, DMSO-d_6): δ 13.21 (s, 1H), 8.86 (s, 1H), 8.57-8.53 (m, 1H), 8.01-7.96 (m, 1H), 7.78 (s, 1H), 7.54-7.48 (m, 1H), 4.04 (s, 2H), 2.71 (s, 3H), 2.21 (s, 3H). ^{13}C NMR (125 MHz, DMSO-d_6): δ 204.09, 175.95, 165.34, 162.76, 161.07, 149.32, 148.92, 144.96, 131.06, 125.75, 124.60, 115.78, 106.38, 47.85, 30.23, 14.36. HRMS (ESI) *m/z* Calcd. for $C_{16}H_{15}N_4O_3S^+$ $[M+H]^+$ 343.0859, Found 343.0859. Purity: 96.7 %.

Synthesis of 5-(3-carbamoylphenyl)-2-methyl-*N*-(3-(2-oxopropyl)-1,2,4-thiadiazol-5-yl) furan-3-carboxamide (M17-B19)

To a stirring solution of **M17-B17** (1.84 g, 5.02 mmol) and 30% H_2O_2 (1.8 mL, 17.5 mmol) in EtOH (5 mL) was added 6 mol/L NaOH (0.21 mL, 1.26 mmol) at 50 °C. This solution was stirred overnight and neutralized with 6.0 mol/L HCl. The solution was concentrated, diluted with water (15 mL) and extracted with DCM (3 × 30 mL). The combined organic layer was washed with brine (2 × 50 mL), dried over anhydrous Na_2SO_4, and concentrated to give a residue, which was purified by column-chromatograph (DCM/MeOH) to give compound **M17-B19.**

White solid, yield: 66 %. ^{1}H NMR (500 MHz, DMSO-d_6): δ 13.16 (s, 1H), 8.13 (s, 1H), 8.17 (t, *J* = 1.5 Hz, 1H), 7.86-7.82 (m, 1H), 7.78-7.77 (m, 2H), 7.56 (t, *J* = 8.0 Hz, 1H), 7.50 (s, 1H), 4.04 (s, 2H), 2.73 (s, 3H), 2.21 (s, 3H). ^{13}C NMR (125 MHz, DMSO-d_6): δ204.10, 176.04, 167.88, 165.37, 162.95, 160.54, 151.10, 135.61, 129.74, 129.64, 127.25, 126.25, 123.11, 115.78, 105.59, 47.87, 30.23, 14.36. HRMS (ESI) *m/z* Calcd. for $C_{18}H_{16}N_4NaO_4S^+$ $[M+Na]^+$ 407.0784, Found 407.0780. Purity: 96.7 %.

Synthesis of 5-(3-aminophenyl)-2-methyl-*N*-(3-(2-oxopropyl)-1,2,4-thiadiazol-5-yl)furan-3-carboxamide (M17-B20)

A suspension of compound **M17-B14** (100 mg, 0.26 mmol) and stannous chloride dihydrate (204 mg, 0.90 mmol) in EtOH (8 mL) was heated to 70 °C and stirred for 5 h. The reaction mixture was cooled to room temperature, alkalized with saturated aqueous $NaHCO_3$ to pH = 10 and filtrated. The filtrate was extracted with EtOAc (3 × 20 mL), and the combined organic layer was washed with brine (2 × 30 mL), dried over anhydrous Na_2SO_4, and concentrated to give a residue, which was purified by column-chromatograph (DCM/MeOH) to give compound **M17-B20**.

Yellow solid. Yield: 60 %. ^{1}H NMR (500 MHz, DMSO-d_6): δ 13.12 (s, 1H), 7.54 (s, 1H), 7.10 (t, *J* = 8.0 Hz, 1H), 6.87 (t, *J* = 2.0 Hz, 1H), 6.80-6.76 (m, 1H), 6.57-6.52 (m, 1H), 5.30 (s, 2H), 4.04 (s, 2H), 2.68 (s, 3H), 2.21 (s, 3H). ^{13}C NMR (125 MHz, DMSO-d_6): δ 204.10, 176.01, 165.30, 163.04, 159.80, 152.53, 149.67, 130.21, 130.06, 115.41,

114.30, 111.51, 108.86, 103.93, 47.88, 30.22, 14.34. HRMS (ESI) *m/z* Calcd. for $C_{17}H_{17}N_4O_3S^+$ $[M+H]^+$ 357.1016, Found 357.1025. Purity: 96.9 %.

Synthesis of 5-(3-acetamidophenyl)-2-methyl-*N*-(3-(2-oxopropyl)-1,2,4-thiadiazol-5-yl) furan-3-carboxamide (M17-B21)

To a solution of compound **M17-B20** (30 mg, 0.08 mmol) in EtOH/MeCN (1:1, 5 mL) was added acetyl chloride (30 μL, 0.42 mmol) at 0 °C. The solution was heated to reflux and stirred for 6 h. Then, the solution was cooled to room temperature and water (20 mL) was added. The mixture was extracted with EtOAc (3 × 10 mL), and the combined organic layer was washed with brine (2 × 20 mL), dried over anhydrous Na_2SO_4, and concentrated to give a residue, which was purified by column-chromatograph (DCM/MeOH) to give compound **M17-B21**.

Yellow solid, yield: 54 %. ^{1}H NMR (500 MHz, DMSO-d_6): δ 13.18 (s, 1H), 10.11 (s, 1H), 8.07 (t, *J* = 2.0 Hz, 1H), 7.68 (s, 1H), 7.48-7.44 (m, 1H), 7.39 (t, *J* = 8.0 Hz, 1H), 7.32-7.28 (m, 1H), 4.04 (s, 2H), 2.71 (s, 3H), 2.21 (s, 3H), 2.08 (s, 3H). ^{13}C NMR (125 MHz, DMSO-d_6): δ 204.12, 176.00, 169.03, 165.35, 162.93, 160.37, 151.61, 140.50, 130.06, 130.04, 118.97, 118.42, 115.60, 114.07, 104.89, 47.87, 30.24, 24.58, 14.37. HRMS (ESI) *m/z* Calcd. for $C_{19}H_{18}N_4NaO_4S^+$ $[M+Na]^+$ 421.0941, Found 421.0938. Purity: 96.2 %.

Synthesis of 5-(3-hydroxyphenyl)-2-methyl-*N*-(3-(2-oxopropyl)-1,2,4-thiadiazol-5-yl)furan -3-carboxamide (M17-B22)

To a solution of compound **M17-B18** (100 mg, 0.27 mmol) in anhydrous DCM (6 mL) was slowly added boron tribromide (2.0 mol/L in DCM, 404 μL, 0.81 mmol) under nitrogen atmosphere at -78 °C. Then the solution was warmed to room temperature, stirred overnight and quenched with water (20 mL). The mixture was extracted with EtOAc (3 × 20 mL), and the combined organic layer was washed with brine (2 × 30 mL), dried over anhydrous Na_2SO_4, and concentrated to give a residue, which was purified by column-chromatograph (EtOAc/PE) to give compound **M17-B22**.

White solid. Yield: 73 %. ^{1}H NMR (500 MHz, DMSO-d_6): δ 13.13 (s, 1H), 9.69 (s, 1H), 7.61 (s, 1H), 7.27 (t, J = 8.0 Hz, 1H), 7.09-7.02 (m, 2H), 6.77-7.73 (m, 1H), 4.04 (s, 2H), 2.69 (s, 3H), 2.20 (s, 3H). ^{13}C NMR (125 MHz, DMSO-d_6): δ 204.09, 176.00, 165.34, 162.94, 160.17, 158.31, 151.73, 130.85, 130.76, 115.76, 115.53, 114.67, 110.40, 104.65, 47.87, 30.23, 14.35. HRMS (ESI) *m/z* Calcd. for $C_{17}H_{16}N_3O_4S^+$ [M+H]$^+$ 358.0856, Found 358.0849. Purity: 97.1 %.

Synthesis of 3-(5-methyl-4-((3-(2-oxopropyl)-1,2,4-thiadiazol-5-yl)carbamoyl)furan-2-yl) phenyl acetate (M17-B23)

To a solution of compound **M17-B22** (50 mg, 0.14 mmol) in MeCN (5 mL) was added triethylamine (20 μL, 0.14 mmol) and acetyl chloride (20 μL, 0.28 mmol) at 0 °C. The solution was warmed to room temperature, stirred for 6 h, and then water (20 mL) was added. The mixture was extracted with EtOAc (3 × 10 mL), and the combined organic layer was washed with brine (2 × 20 mL), dried over anhydrous Na_2SO_4, and concentrated to give a residue, which was purified by column-chromatograph (EtOAc/PE) to give compound **M17-B23**.

White solid. Yield: 62 %. ^{1}H NMR (500 MHz, DMSO-d_6): δ 13.15 (s, 1H), 7.72 (s, 1H), 7.55-7.49 (m, 2H), 7.41-7.38 (m, 1H), 7.14-7.10 (m, 1H), 4.04 (s, 2H), 2.70 (s, 3H), 2.31 (s, 3H), 2.21 (s, 3H). ^{13}C NMR (125 MHz, DMSO-d_6): δ 204.08, 175.96, 169.64, 165.38, 162.85, 160.62, 151.56, 150.63, 130.98, 130.94, 121.89, 121.14, 117.20, 115.75, 105.77, 47.87, 30.23, 21.37, 14.34. HRMS (ESI) *m/z* Calcd. for $C_{19}H_{18}N_3O_5S^+$ [M+H]$^+$ 400.0962, Found 400.0951. Purity: 95.9 %.

Construction of the AR^{W751R} and AR^{F754V} Mutated Models

The AR^{W751R} and AR^{F754V} homodimer models were generated by mutating W751 to R and F754 to V respectively in the crystal structure of the wild type AR LBD dimer in complex with an agonist DHT in each monomer (PDB entry: 5JJM) by the *mutate* module in *Schrödinger* (v.2015).[1] The structures were then prepared by the *Protein Preparation Wizard*

module in *Schrödinger*, including removing all non-bonded hetero-atoms and water molecules, adding missing side chains, and optimizing the structure to relieve steric clashes.

Molecular Dynamics (MD) Simulations for Dimers of AR^{WT}, AR^{W751R} and AR^{F754V}

The three prepared structures were used as the initial structure for the MD simulations. The partial charges of DHTs were obtained using the restrained electrostatic potential (RESP) fitting technique based on the electrostatic potentials computed at the Hartree-Fock (HF) SCF/6-31G* level by *Gaussian16*.[2, 3] Afterwards, the topology and parameter files of DHTs were generated by using the *antechamber* module in *Amber 18*.[2, 4] The molecular mechanics (MM) parameters from the ff14SB and GAFF2 force fields were assigned to the proteins and DHTs, respectively, using the *LEaP* module of *Amber 18* [5, 6]. Each system was solvated into a cubic TIP3P water box with 20 Å away from the surface of the protein complex. Finally, an appropriate number of chloride ions were added to neutralize each system.

A prior multistage equilibration strategy was used to remove unfavorable contacts for each system. First, each system was submitted to 5,000 steps of steepest descent MM minimization, followed by 5,000 steps of conjugate gradient MM minimization to the solvent molecules. Then, the side chains were optimized by 5,000 steps of steepest descent and 5,000 steps of conjugate gradient MM minimizations. Thereafter, 5,000 steps of steepest descent and 5,000 steps of conjugate gradient MM minimizations were conducted to the water box. Afterwards, each system was heated to 300 K over a period of 0.2 ns, and then equilibrated over 1.5 ns in the NPT ensemble (T = 300 K and P = 1 bar). Finally, each system was submitted to 500 ns NPT (T = 300 K and P = 1 bar) MD simulations. In all the stages, the temperature was controlled by the Langevin temperature equilibration scheme with a collision frequency of 2.0 ps^{-1} and the pressure was controlled by using a Berendsen barostat.[7, 8] The particle mesh Ewald (PME) algorithm was applied to handle the long-range electrostatic interactions under periodic

boundary condition and a cutoff of 8 Å was used for the van der Waals interactions.[9] The SHAKE method was employed to constrain all covalent bonds of hydrogen atoms.[10] The simulation time step was set as 2 fs and the snapshot was recorded every 10 ps.

SBVS Protocol

The SBVS was employed using the *Virtual Screening Workflow* module in *Schrödinger* through cascade docking, including drug-likeness filtering via Lipinski's Rule-of-five, Glide standard precision (SP) mode docking, and Glide extra precision (XP) mode docking.[11] The top-ranked 5,000 compounds were structural clustered by the Tanimoto coefficient based on the hashed linear fingerprints using the *Canvas* module in *Schrödinger*. The binding poses of the clustered compounds were manually inspected and filtered. Finally, 476 potential compounds were purchased from ChemDiv chemical library for in vitro bioassays.

MD Simulations for AR LBD Monomer Bound with M17

The docked structure of the AR LBD bound with M17 was selected as the initial structure for the 1,000 ns long-time MD simulations. The partial charges of M17 were calculated by *Gaussian16*.[2, 3] Then, the *antechamber* module in *Amber 18* package were employed to generate the topology and parameter files of M17.[2, 4] The *LEaP* module in *Amber 18* were employed to assign the ff14SB and GAFF2 force fields to the AR LBD and M17, respectively.[5, 6] Other system parameters of preparation, minimization, heating, equilibrium procedures were same as methods of 'Molecular Dynamics (MD) Simulations for Dimers of AR^{WT}, AR^{W751R} and AR^{F754V}'. The system was submitted to three independent 1,000 ns MD simulations and the trajectories from 800-1,000 ns with 1,000 snapshots were applied for binding free energy calculation and per-residue decomposition based on MM/GBSA method as our previous reported. [11, 12]

Cell Culture

Cell lines of LNCaP, 22RV1, DU145, PC3, C4-2, NIH-3T3, A549, NCI-H1299, SW480, U2OS, HEK293T were purchased from ATCC (Manassas, VA, USA). Chang, MCF-7, HepG2, and HeLa were purchased from the Cell bank of Chinese Academy of Sciences (Shanghai, China). Cell lines of GES-1 and HL60 were gifted by Prof. Hongchuan Jin (Sir Run Run Shaw Hospital, Hangzhou, China) and Prof. Meidan Ying (Zhejiang University, Hangzhou, China), respectively. The cell lines of LNCaP, 22RV1, PC3, DU145, C4-2, GES-1, Chang, and H1299 were cultured in RPMI-1640 (Sigma-Aldrich, #R8758) supplemented with 10% fetal bovine serum (FBS, #10099141C, Gibco). Cell lines of NIH-3T3, A549, MCF-7, HepG2, U87, U2OS, SW480, HeLa, HEK293T were cultured in Dulbecco's modified Eagle's medium (DMEM, #D6429, Sigma-Aldrich) supplemented with 10% FBS. Cell line of HL60 was cultured in Iscove-modified Dulbecco medium (#12440053, Gibco) supplemented with 20% FBS. All the cell lines were cultured in a humidified, 5% CO_2-containing atmosphere incubator.

AR Transcriptional Activity Assay

The AR transcriptional activities were determined using the constructed LNCaP-ARR_2PB-eGFP cell as previously reported.[11, 12] The cells of LNCaP-ARR_2PB-eGFP were starved with 5% charcoal-stripped serum (CSS) in RPMI-1640 media for 5 days, followed by seeding into a 96-well plate (3.5×10^4 cells/well) for 1 day incubation at 37 °C. Then, the cells were treated with 5 nM DHT and various concentrations of compounds (10 μM for 476 screened compounds, 22.86 nM - 50 μM for M17 and its analogues). After 3 days, the fluorescence intensities were determined using a Synergy H1 microplate reader (BioTek. excitation, 485 nm; emission, 535 nm).

Competitive Ligand Binding Assay

The binding affinities of M17 and M17-B15 to the LBP of AR LBD were determined using PolarScreen™ Androgen Receptor Competitor Assay Kit (#A15880, Invitrogen Life Technologies) according to the user manual.

Secreted Prostate-Specific Antigen (PSA) Assay

After the AR transcriptional activity assay was completed, 300 μl of the media supernatant including the secreted PSA was measured using IMMULITE® 2000 XPi Immunoassay System (Siemens Ltd., Erlangen, Germany).

Cell Viability Assay

3-(4,5-dimethythiazol-2-yl)-2,5-diphenyl-2-H-tetrazolium bromide (MTT) colorimetric assay was employed to investigate cell viability. Cells were seeded into 96-well plates with 2-5×10^3 cells per well (5×10^3 cells/well for LNCaP, 22RV1, NIH-3T3, GES-1, Chang, 3×10^3 cells/well for DU145, PC3, C4-2, A549, MCF-7, HepG2, HeLa, HL60, SW480, NCI-H1299, U2OS, U87) for 1 day incubation at 37 °C. Then, the cells were treated with various concentrations of compounds (22.86 nM - 50 μM) and incubated for 72 h. Then, 10 μl MTT (5 mg/ml) was added into each well and incubated for 4 h. Afterwards, 100 μl of triplex 10% SDS-0.1% HCl-phosophate-buffered saline (PBS) solutions was added into each well and incubated for overnight. The absorbance at 570 nm was measured (Eon, Bioteck, Winooski, VT).

Protein Expression and Purification

The AR LBD (663-919) was fused into the pET50b vector with Nus-tag. The plasmid was then transformed into *E. coli* BL21 (DE3) for protein expression. The *E. coli* was cultured

in 4 L LB media at 37°C until OD600 arrived to 0.6, then supplemented with 0.5 mM Isopropyl β-D-1-Thiogalactopyranoside (IPTG) and 20 μM DHT. After incubated at 16 °C for 16-18 h, the cells were collected by centrifugation at 3,500 × g for 0.5 h. The cell mass was resuspended in Lysis buffer (20 mM Tris-HCL pH 7.4, 5 mM imidazole,150 mM NaCl, 10% Glycerol, 2 mM Dithiothreitol (DTT) and 10 μM DHT) and lysed at 800 bar for at least 5 cycles. The lysis was centrifugated at 14,000 × g for 0.5 h, and the supernatant was loaded to HisTrap™ Heparin HP column (#17524802, GE Healthcare), followed by washing and eluting steps using 65 mM imidazole and 300 mM imidazole, respectively. The elution from Ni-NTA were cut by HRV-3C protease, and further purified by HiLoad™ 16/600 Superdex 200 (#28989335, GE Healthcare) using S200 buffer (20 mM Hepes pH7.4, 5% Glycerol, 150 mM NaCl, 2 mM DTT and 10 μM DHT). The purified protein was concentrated and stored at -80 °C.

BLI Assay

The purified AR LBD protein was biotinylated using EZ-Link™ Sulfo-NHS-LC-LC-Biotin (#A35358, ThermoFisher) in 500 μL phosphate-buffered saline with 0.02% Tween-20 (v/v, PBST) buffer for 2 h at 4 °C. Then, the protein was dialyzed using a Slide-A-Lyzer dialysis cassettes for 3 times with an 8 h interval change of the PBST buffer at 4 °C. BLI assay was performed using a FortéBio Octet Red system. The biotinylated protein in 250 μL PBST buffer was loaded to super streptavidin (SSA) sensors and equilibrated for 300 s. The kinetics of the AR LBD and M17-B15 association was evaluated by soaking the AR LBD-loaded sensors into wells containing various concentrations of M17-B15 (6.25, 12.5, 25, 50, 100 μM) for 60 s, followed by dissociation of M17-B15 by soaking the sensors in the PBST buffer for 60 s. The kinetics values were calculated by fitting the kinetic data based on a 1:1 binding model using FortéBio data analysis (v.11.1) software.

CXMS Analysis

30 μM AR LBD protein was crosslinked with a 50× molar excess of freshly prepared BS^3 (#21580, ThermoFisher) in 20 mM HEPES (pH 7.4), 5% Glycerol, 150 mM NaCl, 10 μM DHT, with or without 90 μM M17-B15. The cross-linking reaction was conducted at room temperature with gentle mixing, and was quenched after 45 min with the addition of Tris-HCl buffer (pH 8.0) to a concentration of 20 mM.

Cross-linked proteins were precipitated with 6-fold volume of pre-cooled acetone for 2 h at -20 °C. Centrifuge at 14,000 rpm for 20 min, and discard the supernatant. Then added 20 μL 100 mM Tris-HCl buffer (pH 8.5), 8 M urea, and ultrasound for 20 min to assist protein dissolution. 5 mM reducing agent TCEP, 10 mM alkylating agent iodoacetamide, 1 mM $CaCl_2$ and 20 mM methylamine were added in tandem. And 3-fold volume of 100 mM Tris pH 8.5 was added to dilute urea to 2 M. Then added trypsin (the mass ratio of enzyme to protein is 1:50) and digested the protein samples at 37 °C for 14 h. Finally, quenched the digestion reaction by adding formic acid with a final concentration of 5%.

An Easy nLC 1000 system (ThermoFisher) coupled to a Velos Pro Orbitrap Elite mass spectrometer (ThermoFisher) was used for mass spectrometry analysis. The resulting peptide mixture was vacuum-centrifuged to dryness, and reconstituted in 0.1% formic acid. Then peptides were loaded onto a pre-column (100 μm × 2 cm) and further separated on a capillary column (75 μm × 10 cm) with laser-pulled sprayer. Luna 3 μm C18 bulk packing material (Phenomenex) was used to pack the columns. And peptides were separated with a reverse-phase gradient: 2-35% buffer B in 90 min, 35-80% buffer B in 4 min, then held at 80% buffer B for 10 min (buffer A = 0.1% formic acid in water, buffer B = 0.1% formic acid in acetonitrile) at a flow rate of 300 nL/min. Data-dependent mode with a full MS scan (350-1500 M/z) in the FT mode at a resolution of 120,000 was performed in mass spectrometry. Automatic gain control (AGC) targets were 1E×6 ions for Orbitrap scans and 5E×4 for MS/MS scans. Excluded 1+, 2+, more than 7+, and unassigned charge state during data collection.

Data processing was carried out using *pLink2* (v.2.3.8).[13] The pLink search parameters

include, 1) Cys carbamidomethyl selected as a static modification and Met oxidation a variable modification; 2) precursor mass tolerance of 20 ppm; 3) fragment mass tolerance of 20 ppm; 4) cross-linker BS^3 was selected with a cross-link mass-shift of 138.068 Da and a mono-link mass-shift of 156.079 Da; 5) a minimum peptide length of 3 amino acids and a maximum peptide length of 60 amino acids; 6) a minimum peptide mass of 300 Da and a maximum of 6,000 Da; 7) enzymatic digestion by trypsin with up to 3 missed cleavage sites per cross-linked peptide. The protein sequence AR LBD was used for database searching. The results were filtered with ≤10 ppm in mass and ≤5% in false discovery rate (FDR) at the PSM level.

Structure Modeling of AR LBD Dimer According to the CXMS results

The structure modeling was performed using *Xplor-NIH* (v.3.3).[14] Crystal structure, PDB code 5V8Q,[15] was used as the starting coordinate for the monomer. A total of 37 and 24 cross-linking restraints were applied to Lys C_β atoms for modeling the AR LBD dimer structure either without the M17-B15 or with the M17-B15 bound, respectively. Cross-linking involving the same residue (for example, K777-K777', K825-K825', and K845-K845') is specified as intermolecular. For other cross-link pairs, ambiguous distance restraints were used, without specifying intramolecular or intermolecular.

During the refinement, one AR LBD subunit was fixed while the other AR LBD subunit was grouped as a rigid body and allowed to reorient. The simulation annealing protocol has been described previously.[16, 17] In addition to the cross-linking distance restraints (an upper-bound of 20.4 Å between cross-linked C_β atoms), a weak radius-of-gyration restraint and van der Waals repulsive energy terms are also included. The number of conformers has to be increased to 7 (ensemble size N = 7) to account for all cross-linking restraints. The refinement was repeated 1,200 times with different random seeds. The ensemble structures that satisfy all the restraints and have no steric clashes were selected for further analysis.

Small-Angle X-ray Scattering (SAXS) Analysis

The SAXS data for the free or antagonist-bound AR LBD were collected at the National Center for Protein Science Shanghai using the BL19U2 beamline at 25 °C. The concentrations of proteins for AR^{WT}, AR^{W751R} and AR^{F754V} were 35 μM. The concentrations for DHT, Enz and M17-B15 were 10 μM, 105 μM, and 105 μM, respectively. The SAXS scattering signal were collected for 20 frames (1 s exposure time), and further averaged to produce the final data. The scattering profile for matching buffer was also recorded and was subtracted. The theoretical scattering profile takes into consideration of both AR LBD monomer and dimer in solution. To estimate monomer/dimer ratio and to assess the structural change upon inhibitor binding, we took the following two steps in data analysis. First, we calculate the theoretical scattering curve of monomer based on the crystal structure (PDB code 5V8Q) using *CRYSOL* modules in the *ATSAS* (v.2.8) software package.[18] We then varied the monomer ratio stepwise, from 50% to 95%, in 5% increment, which contribution was subtracted from the experimental value at each angle for the dimer contribution. Subsequently, we used Genetic Algorithm Judging Optimization of Ensembles (*GAJOE*) [19] to select the dimer conformations that can best satisfy the dimer scattering. The library of dimer conformers (containing 8,300 structures) is obtained from the CXMS-based structure modeling. Each structure contains 7 conformers that satisfy all CXMS restraints discussed above. The selection process was repeated 100 times with default parameters in *GAJOE* to identify the ensemble with the smallest χ^2 value.

Luciferase Reporter Assays for FL-AR^{F876L} and FL-$AR^{F876L/T877A}$

The full-length the F876L (FL-AR^{F876L}) and F876L/T877A (FL-$AR^{F876L/T877A}$) mutants were constructed on the basis of the human full length wild type AR plasmid pCMV-hAR (#89078, Addgene) using the primer pairs in Table S5. PC3 cells (1.2 × 10^4/well) were transfected with 70 ng/well of FL-AR plasmid together with 24 ng of ARR2PB-Luc, and 5 ng of Renilla. After 1 day incubation at 37 °C, the antagonistic models were employed

by the cells treated with 10 nM DHT and gradient concentrations of Enz and M17-B15 (22.86 nM - 50 μM). After another 24 h incubation, the luciferase activities were determined by the Dual-Glo Luciferase system (#E1910, Promega) according to the standard protocol.

Acceptor Photobleaching Fluorescence Resonance Energy Transfer (FRET) Microscopy Assay

The pCMV-cyan fluorescent protein (CFP, #D2623) and pCMV-yellow fluorescent protein (YFP, #D2716) plasmids were purchased from Beyotime Biotechnology. We constructed pCMV-YFP-(AR LBD) and pCMV-(AR LBD)-CFP by cloning human AR LBD residues (613-920) into the fluorescent protein vector using seamless cloning kit (#C112-01, CloneExpress II, Vazyme). HEK293T (1×10^5 cells/well) cells were cultured in 5% CSS DMEM media in 12-well plate with glass coverslips. The next day, cells were transfected with 400 ng pCMV-YFP-(AR LBD), 400 ng pCMV-(AR LBD)-CFP together with HG-Trans Gene™ transfection reagent (#TG-10012, Health & Gene™). Free YFP and CFP proteins were transfected together as control. YFP-CFP fusion was also transfected as control. After 4 h incubation, the medium was substituted by 5% CSS DMEM containing 10 nM DHT and 10 μM M17-B15.

The FRET assay was then tested by Olympus FV1000 equipped with a UPlanSApo 60×/1.35 NA oil objective. CFP was detected using 405 nm excitation while YFP was excited using 515 nm at moderate laser power, and the emission was detected using a 480-495 nm bandpass emission filter and 535-565 nm bandpass emission filter, respectively. The first pair of CFP_{before} and YFP_{before} was collected. Then YFP was bleached by scanning 3 seconds at 515 nm at high laser power. After photobleaching, the second pair of CFP_{after} and YFP_{after} was collected. The apparent FRET efficiency was calculated after background subtraction as shown:

$$\mathrm{abFRET} = \frac{(\mathrm{CFP_{after}} - \mathrm{CFP_{before}}) \times \mathrm{YFP_{before}}}{(\mathrm{YFP_{before}} - \mathrm{YFP_{after}}) \times \mathrm{CFP_{after}}}$$

And then, the abFRET efficiency was compared to $abFRET_{YFP\text{-}CFP}$ (YFP-CFP fusion protein) and $abFRET_0$ (free YFP and CFP protein):

$$apparentFRETefficiency = \frac{abFRET - abFRET_0}{abFRET_{YFP-CFP} - abFRET_0}$$

Cell Culture Sampling and Transcriptome Sequencing

The LNCaP cells were treated with 10 nM DHT, 10 nM DHT and 10 μM Enz, 10 nM DHT and 10 μM M17-B15, respectively. After incubation of 48 h, the cells were harvested and submitted to Novogene (Beijing, China) to perform transcriptome sequencing. Low-quality bases in the RNA-Seq raw reads were first filtered using *Fast*QC c and *Trimmomatic* (v.0.40) software.[20] *Trinity* (v.2.10.0) package were used to de novo assembly of transcriptomes based on the clean reads.[21] The expression abundance for each transcript was calculated according to the fragments per kilobase of exon per million fragments mapped (FPKM) value using *HISat2* (v.2.1.0) and *Subread* (v.2.0.1) software.[22, 23] Differentially expressed unigenes (DEGs) were identified among different conditions using *DESeq2* (v.1.32.0) *R* package with a FDR < 10^{-5} and |log2 (fold-change)| > 0 as the criteria.[24] Biological function annotation for DEGs was accomplished using NR public databases and *BLASTX* (v.2.9.0) analysis and the cut-off E-value was set as 10^{-5}. We generated Venn diagrams using the Draw Venn Diagram tool (http://bioinformatics.psb.ugent.be/webtools/Venn/).

Analysis of Sequences and Structures of Nuclear Receptor Ligand Binding Domains (NR LBDs)

The sequences of 48 NR LBDs were retrieved from *UniProt* database. Then, these sequences were applied to construct phylogenetic tree using *Mega-X* (v.10.2.5).[25] The Phylogenetic tree was visualized using iTOL online tool.[26] The structures of NR LBDs were retrieved from *Protein Data Bank* database. The unreported structures of NR LBDs were homology modeled using the *SWISS-MODEL* web server.[27] The structures were

aligned and the binding site were analyzed using *Multiple Sequence Viewer* module in *Schrödinger*.

Clonogenic Assay

1.5×10^3 cells/well of LNCaP or 22RV1 were seeded into a 6-well plate. After 1 day incubation, the cells were treated with DMSO, Enz or M17-B15. After 16-18 days incubation, the medium of each well was removed and washed thrice using PBS. Finally, the cell colonies were fixed by methanol, followed by staining with 0.1% crystal violet for 25 min.

Quantitative PCR (qPCR) for *PSA, CDC20, CENPF, MKI67*

LNCaP cells were cultured in medium containing 5% CSS in six-well plates and treated with Enz and M17-B15 (0.1, 1 μM, in the presence of 5 nM DHT) for 24 h, respectively, where 5 nM DHT and DMSO were separately used in parallel as controls. After 48 h incubation, EZ-10 DNAaway RNA Mini-Preps Kit (Sangon Biotech) and Hifair® III 1st Strand cDNA Synthesis SuperMix for qPCR (YEASEN) were employed for mRNA extraction and cDNA preparation, respectively. Then, the PCR SYBR Green Master Mix (YEASEN) was employed to amplify cDNA using the prime pairs in Table S5.

Western Blot

LNCaP cells were cultured with 5% CSS RPMI-1640 in 6-well plates (4×10^5/well) and incubated for 24 h. Then, the cells were treated with 0.1, 1, 10 μM of Enz and M17-B15, individually, and all in the presence of 10 nM DHT and incubated for 48 h. Then, the cells were harvested, followed by separating the cell lysate using 10% SDS-PAGE and electro-transfer to a polyvinylidene fluoride (PVDF) membrane (Bio-Rad). Blocking buffer (#P0023B, Beyotime Biotechnology) were used to block the membranes. After 0.5

h incubation at room temperature, each membrane was further incubated with specific primary antibody (Androgen Receptor, #8428, Cell Signaling Technology, Inc.; PSA, #5365, Cell Signaling Technology, Inc.; GAPDH, #AF1186 Beyotime Biotechnology; Lamin B1, #AF1408, Beyotime Biotechnology) and then the corresponding secondary antibody (HRP-conjugated Goat Anti-Rabbit IgG, # D110058, Sangon Biotech). The *Image J* program was performed for quantitative analysis of the immunoreactive bands.

Extraction of Nuclear and Cytoplasmic Fractions

LNCaP cells were cultured with 5% CSS RPMI-1640 in 4 dishes (1×10^6/dish) and incubated for 24 h. Then, the cells were treated with 10 μM of Enz and M17-B15 in the presence of 10 nM DHT and incubated for 16 h. The NE-PER Nuclear and Cytoplasmic Extraction Reagents (#78833, ThermoFisher) were applied to fractionation and extraction of nuclear and cytoplasmic proteins according to manufacturer's protocol.

Xenograft Studies

The animal protocols were in accordance with the guidelines of the Animal Welfare Council of China, and were performed by KCBIO company (Chengdu, China). All animal use and studies were performed in compliance with all relevant ethical regulations, and were approved by the Institutional Animal Care and Use Committee (IACUC) at Kangcheng Biotechnology Co., Ltd, Sichuan, China (IACUC-202010-m-001). In brief, male CB17 SCID mice (6-8 weeks old) were housed at a constant room temperature and fed a standard rodent diet and allowed free access to water. LNCaP cells (1×10^7) suspended in 0.1 ml PBS were injected subcutaneously into the right flank of each CB17 SCID mice. Once tumor size reached an average tumor volume of 110 mm^3, the mice were randomized into three groups with 6 mice per group, and then intratumor injection with vehicle (saline), Enz (2.5 mg/kg/week), and M17-B15 (2.5 mg/kg/week), individually. The tumor volumes were estimated by measuring length (L) and width (W) and

calculating volume ($V= 0.5 \times L \times W^2$). Finally, the mice were sacrificed and the tumors were harvested for hematoxylin and eosin (H&E) staining.

Luciferase Reporter Assays for PR, GR, and MR

PC3, HeLa, and HEK293T cells were cultured using 5% CSS RPMI-1640, 5% CSS RPMI-1640 and 10% FBS DMEM media in 96-well plates for 24 h, respectively. For PR induction, PC3 (1×10^4 cells/well) were transfected with 50 ng pcDNA3-PRB (#89130, Addgene), 24 ng pGL4.36[luc2P MMTV Hygro] (Promega) and 5 ng Renilla using HG-TransGene™ transfection reagent (#TG-10012, Genomeditech) for 4 h. For GR induction, Hela (1×10^4 cells/well) were transfected with 71 ng pCMV-GR11 (#89105, Addgene), 24 ng pGL4.36[luc2P MMTV Hygro] (Promega) and 5 ng Renilla using lip3000 transfection reagent (#L3000075, Thermofisher) for 24 h. For MR induction, HEK293T (1×10^4 cells/well) were transfected with 71 ng pCMV-MR gifted by Liu et al.[28], 24 ng of pGL4.36[luc2P MMTV Hygro] (Promega) and 5 ng Renilla using lip3000 transfection reagent for 24 h. For the antagonistic models, 10 nM/well progesterone for PC3 cells, 1 nM/well dexamethasone for HeLa, and 5 nM/well aldosterone for HEK293T cells were added to stimulate the cells. Meanwhile, each cell line was treated with a series concentration of compounds (0.4, 2, and 10 μM). For the agonistic models, the cells were directly treated with the compounds without any hormones. Endogenous hormones progesterone, dexamethasone, and aldosterone were considered as agonists for PR, GR, and MR, respectively. The luciferase activities were assessed using the Dual-Glo Luciferase system (#E1910, Promega).

Cell Cycle and Apoptosis for LNCaP cells

LNCaP were cultured in 6-well plate with 5×10^5 cells/well, after incubated at 37 °C for 24 h, cells were treated with DMSO, Enz or M17-B15. Cell cycle were determined after 48 h incubation by Cell Cycle Staining Kit (#CCS012, MULTI SCIENCES) using

Cytomic FC 500MCL flow cytometer (Beckman Coulter, Inc). Cell apoptosis were determined after 24 h incubation by Annexin V-FITC/PI apoptosis kit (#AP101, MULTI SCIENCES) using BD FACS S ORP ARIA II flow cytometer (BD Biosciences).

Statistical Analysis

Graphpad Prism (v.8.4) program was adopted for statistical analysis and data were presented as the mean ± standard error of mean (SEM). The value of *p* was determined on the basis of at least three independent assays using Student's t-test, one-way ANOVA or two-way ANOVA analysis.

Supplementary Figures

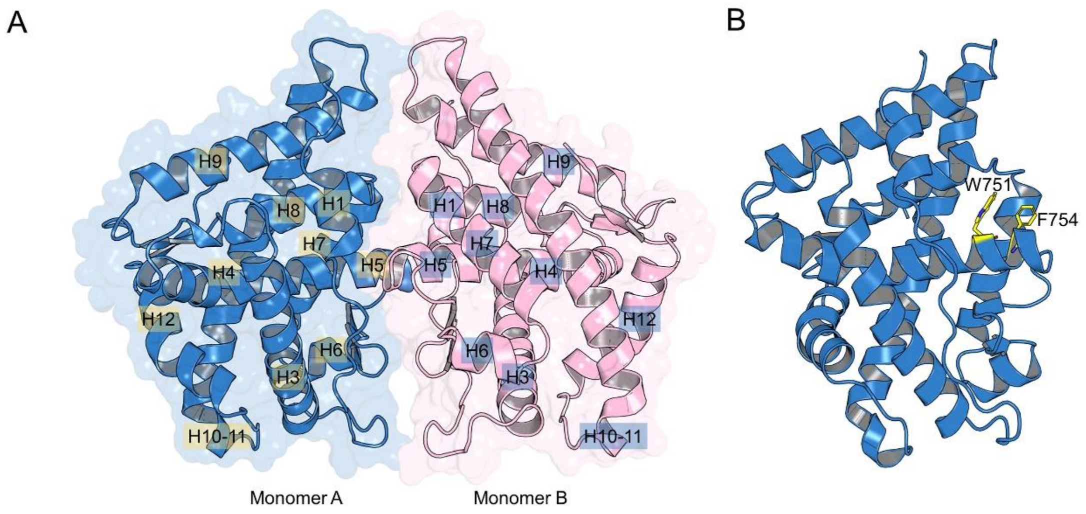

Figure S1. Structure of AR dimer (PDB code: 5JJM). (A) Structure of AR dimer, monomer A (blue) and monomer B (light magenta). (B) The W751 and F754 locates in the AR dimer interface.

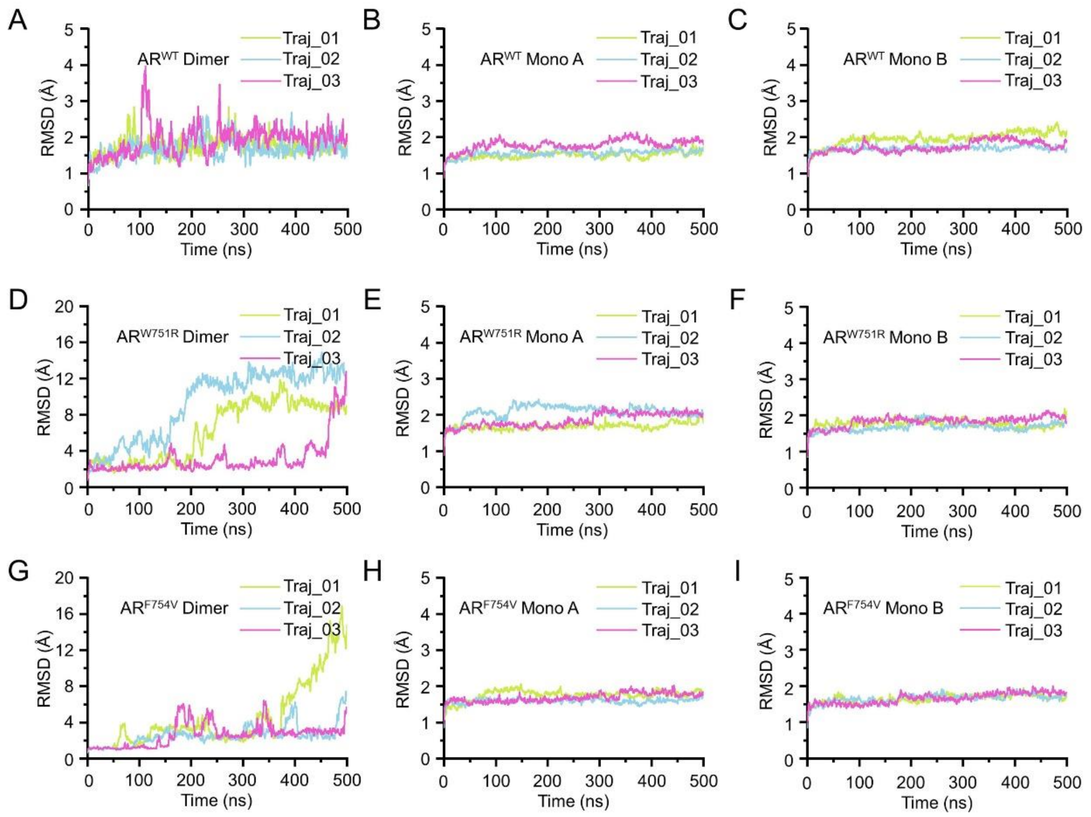

Figure S2. AIS-associated mutations of W751R and F754V inhibit AR LBD dimerization. The RMSDs of the protein backbone C_α atoms of the AR^{WT} LBD of dimer (A), monomer A (B), monomer B (C). The RMSDs of the protein backbone C_α atoms of the AR^{W751R} LBD of dimer (D), monomer A (E), monomer B (F). The RMSDs of the protein backbone C_α atoms of the AR^{F754V} LBD of dimer (G), monomer A (H), monomer B (I).

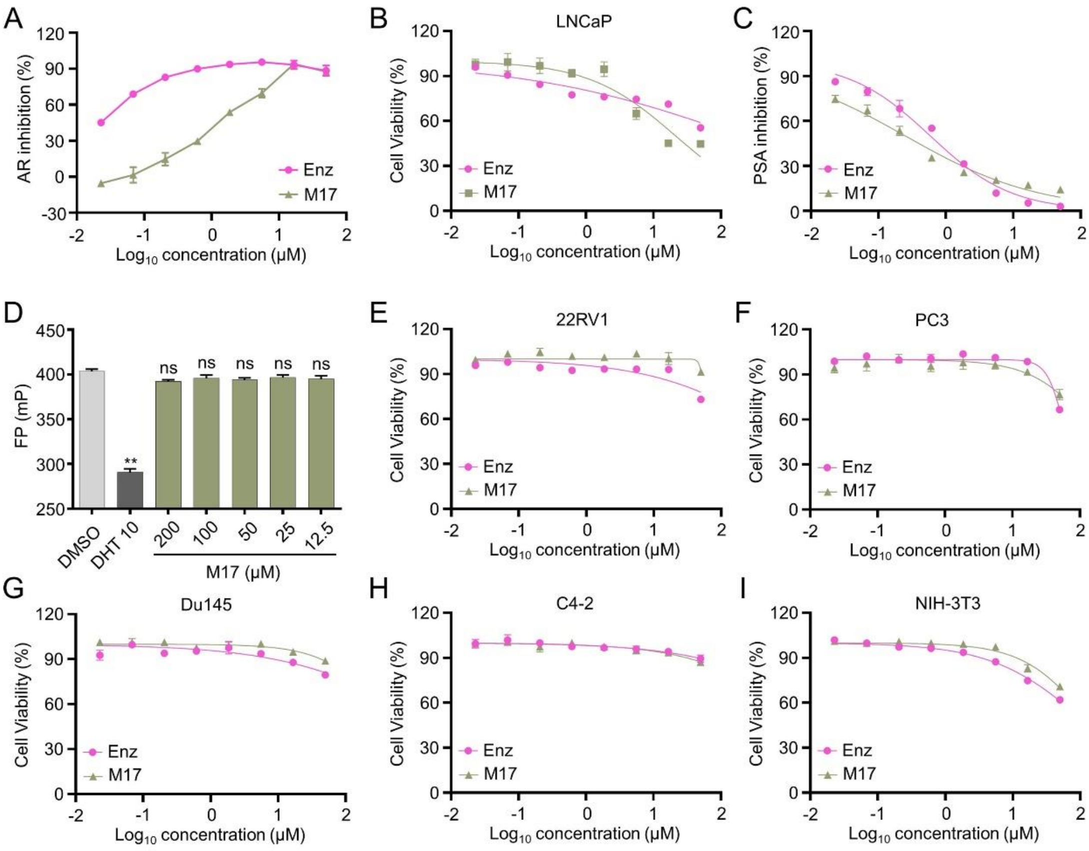

Figure S3. M17 inhibited AR transcription without binding affinity to the LBP of AR LBD. (A) AR transcriptional activity of M17. (B) Cell viability of M17 against LNCaP cells. (C) M17 reduces the PSA levels secreted in the cellular media. (D) Relative LBP of AR LBD binding affinities of the M17 analyzed using PolarScreen™ AR competitor assay. Cell viability of M17 against PCa cells of 22Rv1 (E), PC3 (F), DU145 (G), and C4-2 (H). (I) Cell viability of M17 against normal cells of NIH-3T3. * $P < 0.05$, ** $P < 0.01$ vs. DMSO group. ns, not significant.

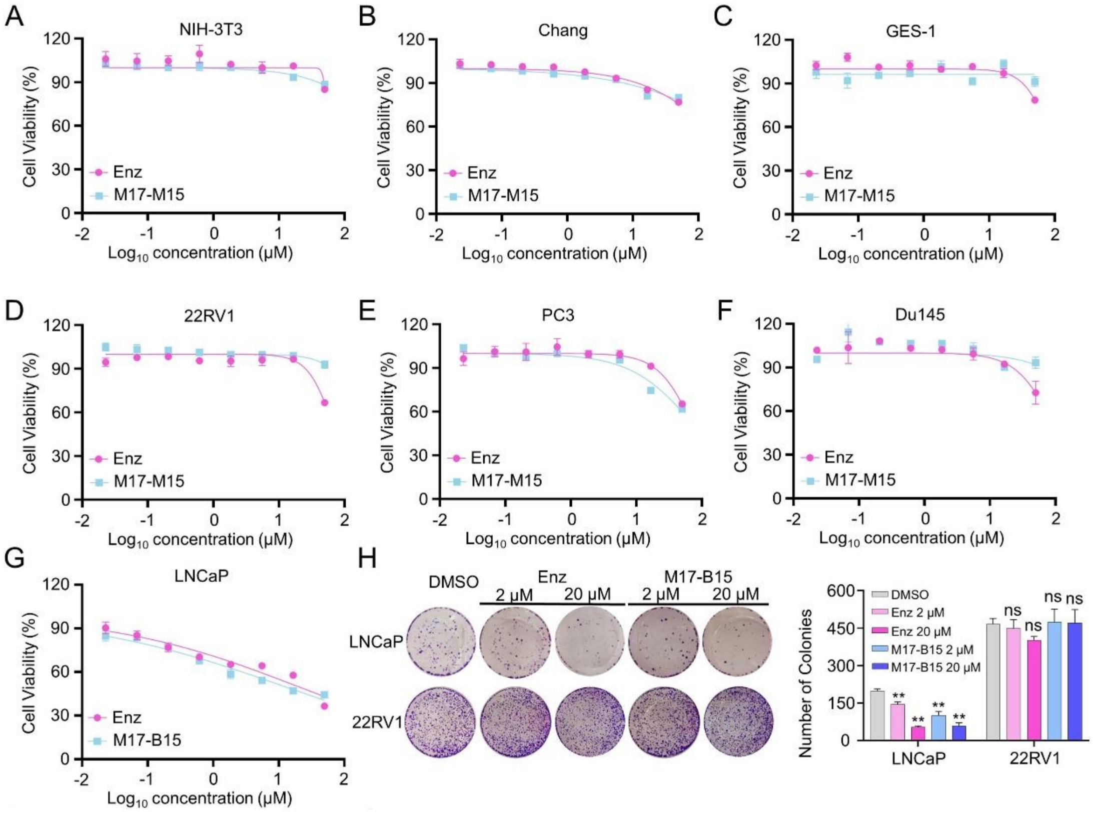

Figure S4. Cell viability of M17-B15 against various of cells. Cell viability of M17-B15 against cells of NIH-3T3 (A), Chang (B), GES-1 (C), 22RV1 (D), PC3 (E), Du145 (F), LNCaP (G). (H) Clonogenic assays of LNCaP and 22RV1 treated with vehicle control (DMSO), 2, and 20 μM of Enz or M17-B15 for 16-18 days. * $P < 0.05$, ** $P < 0.01$ vs. DMSO group; ns, not significant.

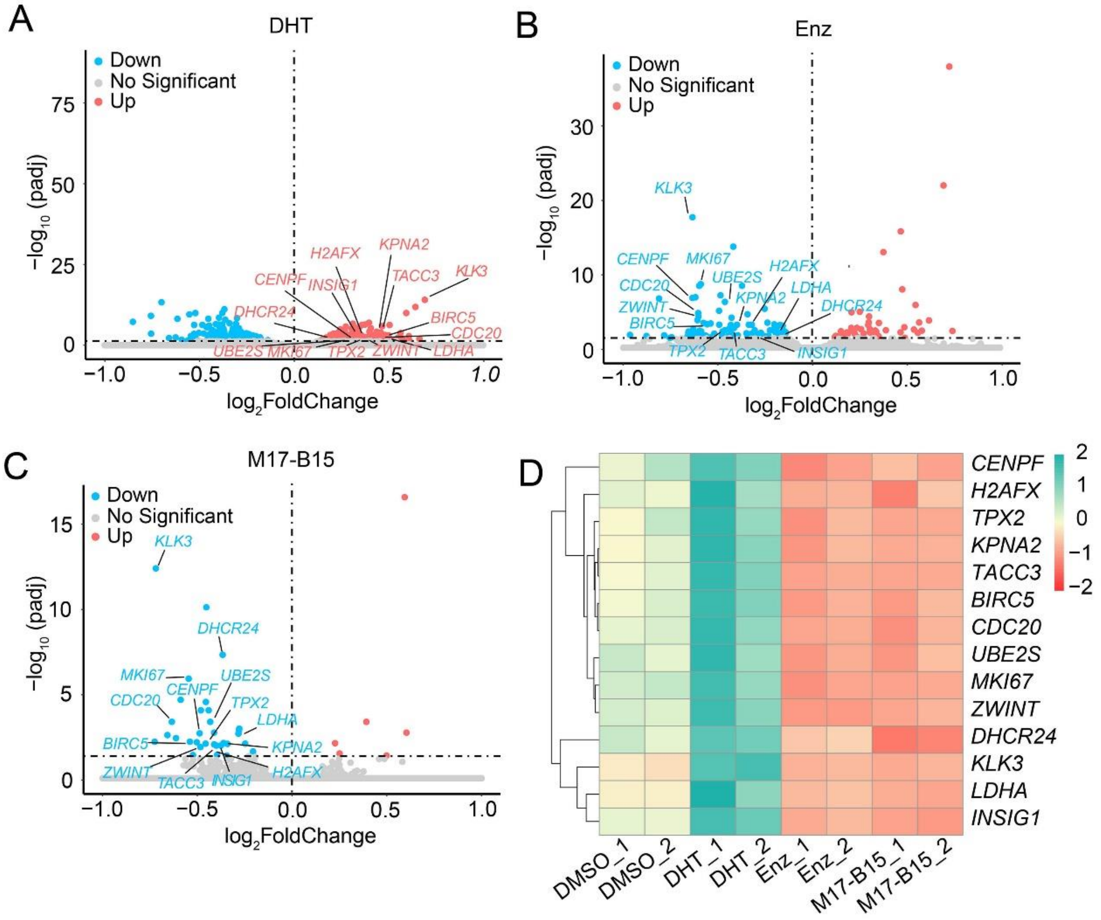

Figure S5. Comparison of differentially expressed genes (DEGs). Gene expression profiling of LNCaP cells after the treatment of DHT (A), Enz (B), and M17-B15 (C). DEGs with fold change > 0 and FDR < 10^{-5} are labeled in red. DEGs with fold change < 0 and FDR < 10^{-5} are labeled in blue. (D) Heatmap of 14 DEGs clustered by hierarchical clustering analysis.

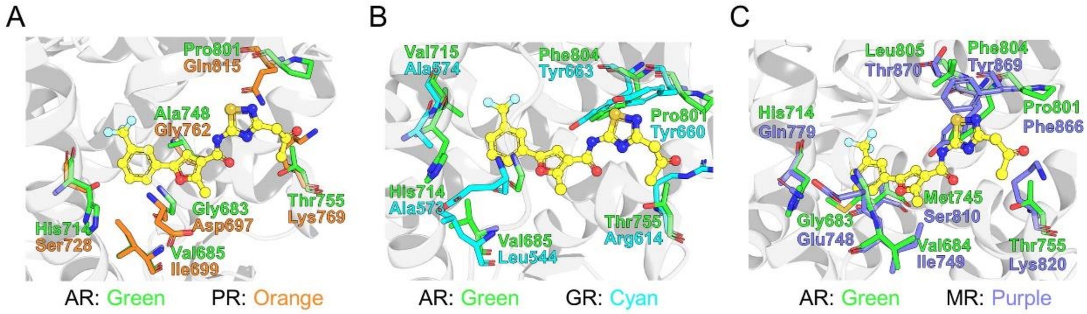

Figure S6. Structural differences of AR DIP toward DIPs of PR, GR and MR. (A) Structural differences between AR DIP (PDB ID: 5JJM) and PR DIP (PDB ID: 1SQN). (B) Structural differences between AR DIP (PDB ID: 5JJM) and GR DIP (PDB ID: 5NFP). (C) Structural differences between AR DIP (PDB ID: 5JJM) and MR DIP (PDB ID: 2A3I). The M17-B15 colored yellow.

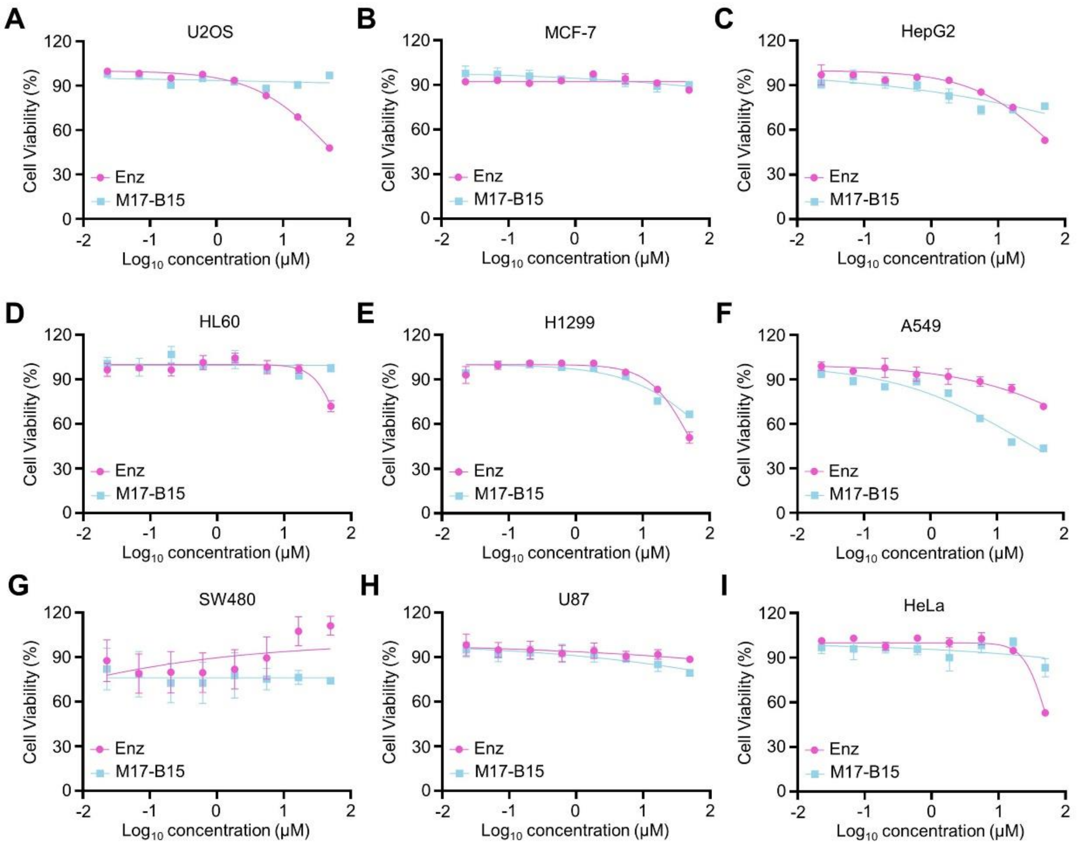

Figure S7. Cell viability of M17-B15 against various of cancer cells. (A) U2OS. (B) MCF-7. (C) HepG2. (D) HL60. (E) H1299. (F) A549. (G) SW480. (H) U87. (I) Hela.

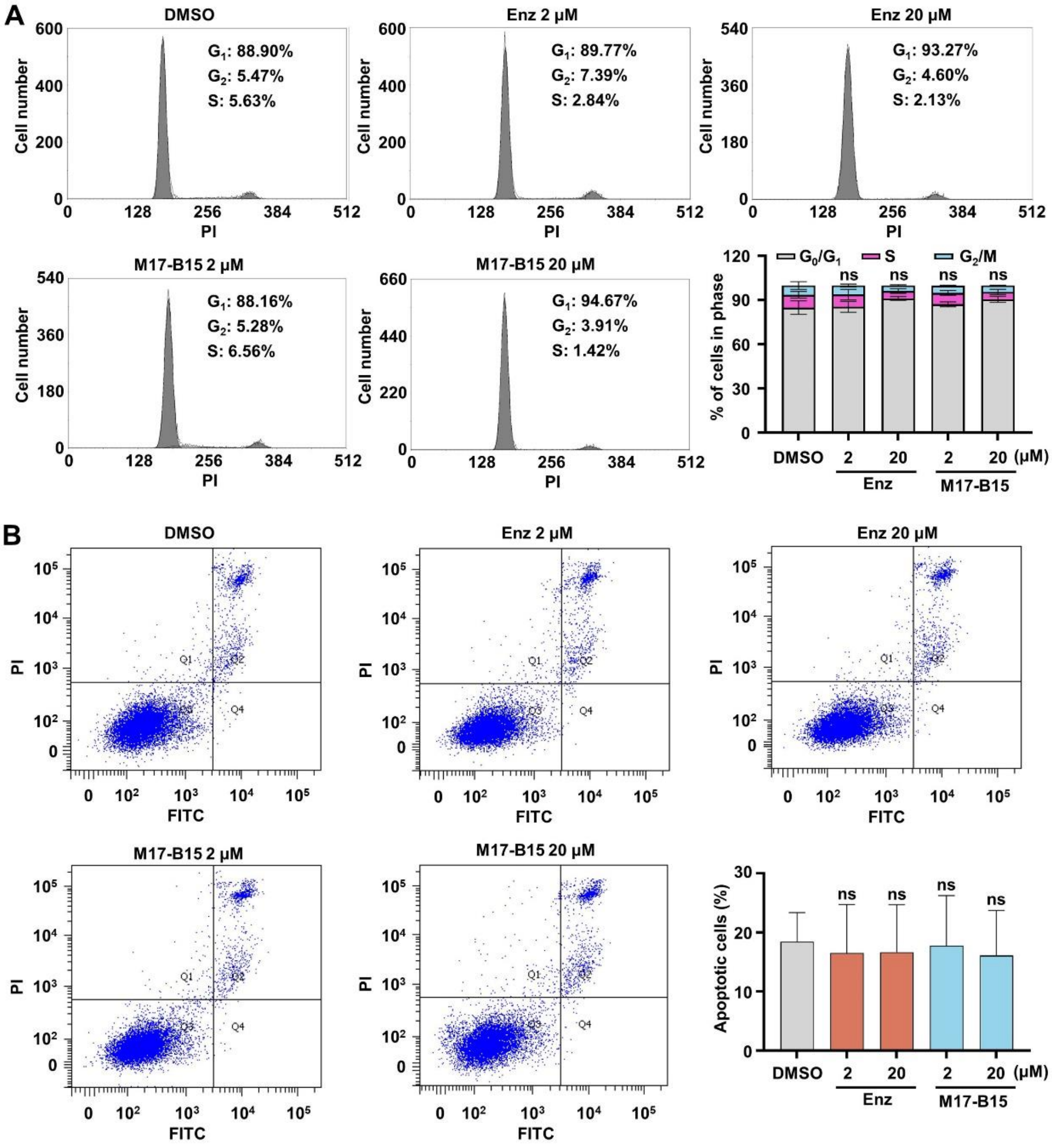

Figure S8. M17-B15 showed no cell cycle progression arrest and apoptosis induction activities on LNCaP cells. (A) The effects of DMSO, Enz or M17-B15 on LNCaP cell cycle distribution. (B) The effects of DMSO, Enz or M17-B15 on LNCaP apoptosis induction activities. * P <0.05, ** P < 0.01 vs. DMSO group; ns, not significant.

Supplementary Tables

Table S1. The source of the 25 analogues of M17

Compound	Source	Compound	Source
M17-A1	D731-0575	M17-B12	Synthesized
M17-A2	Synthesized	M17-B13	Synthesized
M17-B1	Synthesized	M17-B14	Synthesized
M17-B2	Synthesized	M17-B15	Synthesized
M17-B3	Synthesized	M17-B16	Synthesized
M17-B4	Synthesized	M17-B17	Synthesized
M17-B5	6957-0016	M17-B18	Synthesized
M17-B6	D731-0400	M17-B19	Synthesized
M17-B7	D731-0371	M17-B20	Synthesized
M17-B8	7560-0919	M17-B21	Synthesized
M17-B9	6957-0033	M17-B22	Synthesized
M17-B10	Synthesized	M17-B23	Synthesized
M17-B11	D731-0399		

Note: The 8-digit numbers are the compound ID numbers of ChemDiv Vendor

Table S2. Cross-links identified for the LBD of AR alone

Crosslinker	Crosslink type	Cross-linking sites	Peptide	total spectrum	Best E_value
**BS^3**	intra-protein	AR_LBD(836)-AR_LBD(847)	MNYIKELDR(5)-KNPTSCSR(1) / MNYIKELDR(5)-RKNPTSCSR(2) / MNYIKELDR(5)-KNPTSCSRR(1)	31	1.61×10^{-17}
**BS^3**	intra-protein	AR_LBD(836)-AR_LBD(845)	MNYIKELDR(5)-IIACKR(5)	29	1.35×10^{-18}
**BS^3**	intra-protein	AR_LBD(845)-AR_LBD(847)	IIACKR(5)-KNPTSCSR(1) / IIACKRKNPTSCSR(5)-RKNPTSCSR(2) / IIACKR(5)-KNPTSCSRR(1) / IIACKRK(5)-KNPTSCSR(1)	28	1.55×10^{-10}
**BS^3**	inter-protein	AR_LBD(847)-AR_LBD(847)	KNPTSCSR(1)-KNPTSCSR(1) / RKNPTSCSR(2)-KNPTSCSR(1)	16	8.29×10^{-07}
**BS^3**	intra-protein	AR_LBD(777)-AR_LBD(836)	MHKSR(3)-MNYIKELDR(5)	13	3.13×10^{-10}
**BS^3**	intra-protein	AR_LBD(847)-AR_LBD(912)	KNPTSCSR(1)-VKPIYFHTQ(2) / RKNPTSCSR(2)-ILSGKVKPIYFHTQ(7) / RKNPTSCSR(2)-VKPIYFHTQ(2)	12	6.45×10^{-14}

**BS^3**	intra-protein	AR_LBD(825)-AR_LBD(847)	NQKFFDELR(3)-KNPTSCSR(1) / NQKFFDELR(3)-RKNPTSCSR(2)	9	1.23×10^{-15}
**BS^3**	intra-protein	AR_LBD(777)-AR_LBD(847)	MHKSR(3)-KNPTSCSR(1) / MHKSR(3)-RKNPTSCSR(2)	9	5.28×10^{-14}
**BS^3**	intra-protein	AR_LBD(847)-AR_LBD(910)	KNPTSCSR(1)-ILSGKVKPIYFHTQ(5) / RKNPTSCSR(2)-ILSGKVKPIYFHTQ(5)	8	2.14×10^{-15}
**BS^3**	intra-protein	AR_LBD(777)-AR_LBD(825)	MHKSR(3)-NQKFFDELR(3)	8	2.32×10^{-13}
**BS^3**	intra-protein	AR_LBD(836)-AR_LBD(912)	MNYIKELDR(5)-VKPIYFHTQ(2)	8	5.28×10^{-5}
**BS^3**	intra-protein	AR_LBD(720)-AR_LBD(845)	WAKALPGFR(3)-IIACKR(5)	6	1.05×10^{-9}
**BS^3**	intra-protein	AR_LBD(777)-AR_LBD(912)	MHKSR(3)-VKPIYFHTQ(2)	6	2.47×10^{-6}
**BS^3**	intra-protein	AR_LBD(825)-AR_LBD(845)	NQKFFDELR(3)-IIACKR(5)	6	3.55×10^{-14}
**BS^3**	intra-protein	AR_LBD(720)-AR_LBD(847)	WAKALPGFR(3)-KNPTSCSR(1)	5	9.70×10^{-18}
**BS^3**	inter-protein	AR_LBD(836)-AR_LBD(836)	MNYIKELDR(5)-MNYIKELDR(5)	5	1.00
**BS^3**	inter-protein	AR_LBD(777)-AR_LBD(777)	MHKSR(3)-MHKSR(3)	5	1.84×10^{-9}
**BS^3**	inter-protein	AR_LBD(912)-AR_LBD(912)	VKPIYFHTQ(2)-VKPIYFHTQ(2)	5	1.27×10^{-5}
**BS^3**	intra-	AR_LBD(777)-	MHKSR(3)-IIACKR(5)	5	$4.98\times10^{-}$

	protein	AR_LBD(845)			10
**BS^3**	intra-protein	AR_LBD(825)-AR_LBD(836)	NQKFFDELR(3)-MNYIKELDR(5)	4	1.00
**BS^3**	inter-protein	AR_LBD(845)-AR_LBD(845)	IIACKR(5)-IIACKR(5)	4	7.78×10^{-2}
**BS^3**	intra-protein	AR_LBD(825)-AR_LBD(912)	NQKFFDELR(3)-VKPIYFHTQ(2)	4	1.88×10^{-8}
**BS^3**	intra-protein	AR_LBD(717)-AR_LBD(847)	QLVHVVKWAK(7)-KNPTSCSR(1)	4	7.37×10^{-13}
**BS^3**	intra-protein	AR_LBD(845)-AR_LBD(910)	IIACKR(5)-ILSGKVKPIYFHTQ(5)	4	2.83×10^{-5}
**BS^3**	intra-protein	AR_LBD(777)-AR_LBD(910)	MHKSR(3)-ILSGKVKPIYFHTQ(5)	4	2.09×10^{-5}
**BS^3**	intra-protein	AR_LBD(845)-AR_LBD(912)	IIACKR(5)-VKPIYFHTQ(2)	3	6.78×10^{-7}
**BS^3**	intra-protein	AR_LBD(717)-AR_LBD(845)	QLVHVVKWAK(7)-IIACKR(5)	3	2.31×10^{-5}
**BS^3**	intra-protein	AR_LBD(720)-AR_LBD(777)	WAKALPGFR(3)-MHKSR(3)	1	4.62×10^{-22}

Table S3. Cross-links identified for the AR LBD with M17-B15

Crosslinker	Crosslink type	Cross-linking sites	Peptide	total spectrum	Best E values
**BS^3**	intra-protein	AR_LBD(836)-AR_LBD(845)	MNYIKELDR(5)-IIACKR(5)	47	5.88×10^{-17}
**BS^3**	intra-protein	AR_LBD(825)-AR_LBD(836)	NQKFFDELR(3)-MNYIKELDR(5)	42	9.78×10^{-13}
**BS^3**	intra-protein	AR_LBD(836)-AR_LBD(847)	MNYIKELDR(5)-KNPTSCSR(1) / MNYIKELDR(5)-RKNPTSCSR(2) / MNYIKELDRIIACKR(5)-RKNPTSCSR(2) / MNYIKELDR(5)-KNPTSCSRR(1)	36	3.26×10^{-17}
**BS^3**	intra-protein	AR_LBD(845)-AR_LBD(847)	IIACKR(5)-KNPTSCSR(1) / IIACKR(5)-RKNPTSCSR(2) / IIACKRKNPTSCSR(5)-RKNPTSCSR(2)	28	1.61×10^{-10}
**BS^3**	inter-protein	AR_LBD(836)-AR_LBD(836)	MNYIKELDR(5)-MNYIKELDR(5)	23	3.88×10^{-11}
**BS^3**	intra-protein	AR_LBD(777)-	MHKSR(3)-MNYIKELDR(5)	19	4.74×10^{-17}

		AR_LBD(836)			
**BS^3**	intra-protein	AR_LBD(847)-AR_LBD(861)	KNPTSCSR(1)-FYQLTKLLDSVQPIAR(6) / KNPTSCSR(1)-RFYQLTKLLDSVQPIAR(7) / RKNPTSCSR(2)-FYQLTKLLDSVQPIAR(6)	15	2.68×10^{-17}
**BS^3**	intra-protein	AR_LBD(836)-AR_LBD(912)	MNYIKELDR(5)-VKPIYFHTQ(2) / MNYIKELDR(5)-ILSGKVKPIYFHTQ(7)	15	2.11×10^{-9}
**BS^3**	inter-protein	AR_LBD(847)-AR_LBD(847)	KNPTSCSR(1)-KNPTSCSR(1) / RKNPTSCSR(2)-KNPTSCSR(1)	13	5.31×10^{-07}
**BS^3**	intra-protein	AR_LBD(847)-AR_LBD(910)	RKNPTSCSR(2)-ILSGKVKPIYFHTQ(5) / KNPTSCSR(1)-ILSGKVKPIYFHTQ(5)	13	1.50×10^{-12}
**BS^3**	intra-protein	AR_LBD(777)-AR_LBD(861)	MHKSR(3)-FYQLTKLLDSVQPIAR(6)	13	8.18×10^{-10}
**BS^3**	intra-protein	AR_LBD(847)-AR_LBD(912)	KNPTSCSR(1)-VKPIYFHTQ(2) / RKNPTSCSR(2)-ILSGKVKPIYFHTQ(7) / RKNPTSCSR(2)-VKPIYFHTQ(2)	11	5.24×10^{-18}

**BS**^{3}	intra-protein	AR_LBD(825)-AR_LBD(847)	NQKFFDELR(3)-KNPTSCSR(1) / NQKFFDELR(3)-RKNPTSCSR(2)	11	2.02×10^{-15}
**BS**^{3}	intra-protein	AR_LBD(720)-AR_LBD(836)	WAKALPGFR(3)-MNYIKELDR(5)	8	9.67×10^{-12}
**BS**^{3}	intra-protein	AR_LBD(825)-AR_LBD(912)	NQKFFDELR(3)-VKPIYFHTQ(2)	8	4.05×10^{-15}
**BS**^{3}	intra-protein	AR_LBD(777)-AR_LBD(912)	MHKSR(3)-VKPIYFHTQ(2) / MHKSR(3)-ILSGKVKPIYFHTQ(7)	7	7.63×10^{-8}
**BS**^{3}	intra-protein	AR_LBD(845)-AR_LBD(861)	IIACKR(5)-FYQLTKLLDSVQPIAR(6) / IIACKR(5)-RFYQLTKLLDSVQPIAR(7)	7	6.21×10^{-11}
**BS**^{3}	intra-protein	AR_LBD(777)-AR_LBD(825)	MHKSR(3)-NQKFFDELR(3)	7	4.46×10^{-10}
**BS**^{3}	inter-protein	AR_LBD(912)-AR_LBD(912)	VKPIYFHTQ(2)-VKPIYFHTQ(2)	6	6.14×10^{-5}
**BS**^{3}	intra-	AR_LBD(84	IIACKR(5)-VKPIYFHTQ(2)	6	2.88×10^{-6}

	protein	5)-AR_LBD(912)			
BS^3	intra-protein	AR_LBD(777)-AR_LBD(845)	MHKSR(3)-IIACKR(5)	6	6.67×10^{-13}
BS^3	intra-protein	AR_LBD(777)-AR_LBD(847)	MHKSR(3)-KNPTSCSR(1) / MHKSR(3)-RKNPTSCSR(2)	5	1.52×10^{-12}
BS^3	intra-protein	AR_LBD(717)-AR_LBD(847)	QLVHVVKWAK(7)-KNPTSCSR(1) / QLVHVVKWAKALPGFR(7)-RKNPTSCSR(2)	5	4.85×10^{-6}
BS^3	intra-protein	AR_LBD(720)-AR_LBD(825)	WAKALPGFR(3)-NQKFFDELR(3)	5	7.21×10^{-10}
BS^3	intra-protein	AR_LBD(720)-AR_LBD(845)	WAKALPGFR(3)-IIACKR(5)	5	8.93×10^{-11}
BS^3	intra-protein	AR_LBD(720)-AR_LBD(912)	WAKALPGFR(3)-VKPIYFHTQ(2)	5	7.77×10^{-9}
BS^3	inter-protein	AR_LBD(845)-	IIACKR(5)-IIACKR(5)	5	2.94×10^{-10}

		AR_LBD(845)			
**BS**^{3}	intra-protein	AR_LBD(836)-AR_LBD(910)	MNYIKELDR(5)-ILSGKVKPIYFHTQ(5) / FFDELRMNYIKELDR(11)-ILSGKVK(5)	4	4.33×10^{-5}
**BS**^{3}	inter-protein	AR_LBD(777)-AR_LBD(777)	MHKSR(3)-MHKSR(3)	4	2.36×10^{-8}
**BS**^{3}	intra-protein	AR_LBD(825)-AR_LBD(845)	NQKFFDELR(3)-IIACKR(5)	4	3.09×10^{-11}
**BS**^{3}	intra-protein	AR_LBD(910)-AR_LBD(912)	ILSGKVKPIYFHTQ(5)-VKPIYFHTQ(2)	4	1.81×10^{-4}
**BS**^{3}	intra-protein	AR_LBD(845)-AR_LBD(910)	IIACKR(5)-ILSGKVKPIYFHTQ(5)	4	3.74×10^{-8}
**BS**^{3}	intra-protein	AR_LBD(720)-AR_LBD(777)	WAKALPGFR(3)-MHKSR(3)	4	9.78×10^{-7}
**BS**^{3}	intra-protein	AR_LBD(720)-AR_LBD(84	WAKALPGFR(3)-KNPTSCSR(1)	3	5.97×10^{-18}

		7)			
**BS^3**	inter-protein	AR_LBD(825)-AR_LBD(825)	NQKFFDELR(3)-NQKFFDELR(3)	3	1.26×10^{-5}
**BS^3**	intra-protein	AR_LBD(822)-AR_LBD(847)	ALLLFSIIPVDGLKNQK(14)-KNPTSCSR(1)	3	3.54×10^{-6}
**BS^3**	intra-protein	AR_LBD(777)-AR_LBD(910)	MHKSR(3)-ILSGKVKPIYFHTQ(5)	2	4.82×10^{-5}
**BS^3**	intra-protein	AR_LBD(717)-AR_LBD(845)	QLVHVVKWAK(7)-IIACKR(5)	2	5.84×10^{-9}
**BS^3**	intra-protein	AR_LBD(825)-AR_LBD(910)	NQKFFDELR(3)-ILSGKVKPIYFHTQ(5)	1	1.00
**BS^3**	intra-protein	AR_LBD(822)-AR_LBD(845)	ALLLFSIIPVDGLKNQK(14)-IIACKR(5)	1	1.00

Table S4. Differentially expressed genes between DHT, Enz and M17-B15

Gene ID	Gene Name	Gene Description
ENSG00000116133	*DHCR24*	24-dehydrocholesterol reductase
ENSG00000148773	*MKI67*	marker of proliferation Ki-67
ENSG00000108106	*UBE2S*	ubiquitin conjugating enzyme E2 S
ENSG00000134333	*LDHA*	lactate dehydrogenase A
ENSG00000182481	*KPNA2*	karyopherin subunit alpha 2
ENSG00000013810	*TACC3*	transforming acidic coiled-coil containing protein 3
ENSG00000089685	*BIRC5*	baculoviral IAP repeat containing 5
ENSG00000117399	*CDC20*	cell division cycle 20
ENSG00000122952	*ZWINT*	ZW10 interacting kinetochore protein
ENSG00000188486	*H2AFX*	H2A histone family member X
ENSG00000142515	*KLK3*	kallikrein related peptidase 3
ENSG00000186480	*INSIG1*	insulin induced gene 1
ENSG00000117724	*CENPF*	centromere protein F
ENSG00000088325	*TPX2*	TPX2, microtubule nucleation factor

Table S5. Primers used in this study

Primer Name	Sequence (5'->3')
AR^{F876L}_F	AGAGCTGCATCAGCTCACTTTTGACCTGCTAATCAAGTCAC
AR^{F876L}_R	AGCAGGTCAAAAGTGAGCTGATGCAGCTCTCTCGCAATAGG
F876L/T877A-F	AGAGCTGCATCAGCTCGCATTTGACCTGCTAATCAAGTCAC
F876L/T877A-R	AGCAGGTCAAATGCGAGCTGATGCAGCTCTCTCGCAATAGG
PSA_F	GGTGACCAAGTTCATGCTGTG
PSA_R	GTGTCCTTGATCCACTTCCG
CDC20_F	TGGGTTCCTCTGCAGACATTC
CDC20_R	GCTCCTGTAATGGGGAGACC
CENPF_F	CAAGAATATGCACAACGTCCTGC
CENPF_R	GAACGCCTGTTCAGCTCTG
MKI67_F	AGAAGAAGTGGTGCTTCGGAA
MKI67_R	AGTTTGCGTGGCCTGTACTAA

Supplementary Movies

Movie S1. Wild type AR LBD dimer in 1,000 MD simulations

Movie S2. W751R mutant AR LBD dimer in 1,000 MD simulations

Movie S3. F754V mutant AR LBD dimer in 1,000 MD simulations

^{1}H-NMR, ^{13}C-NMR, HRMS spectra, HPLC Traces of All Target Compounds

Compound M17

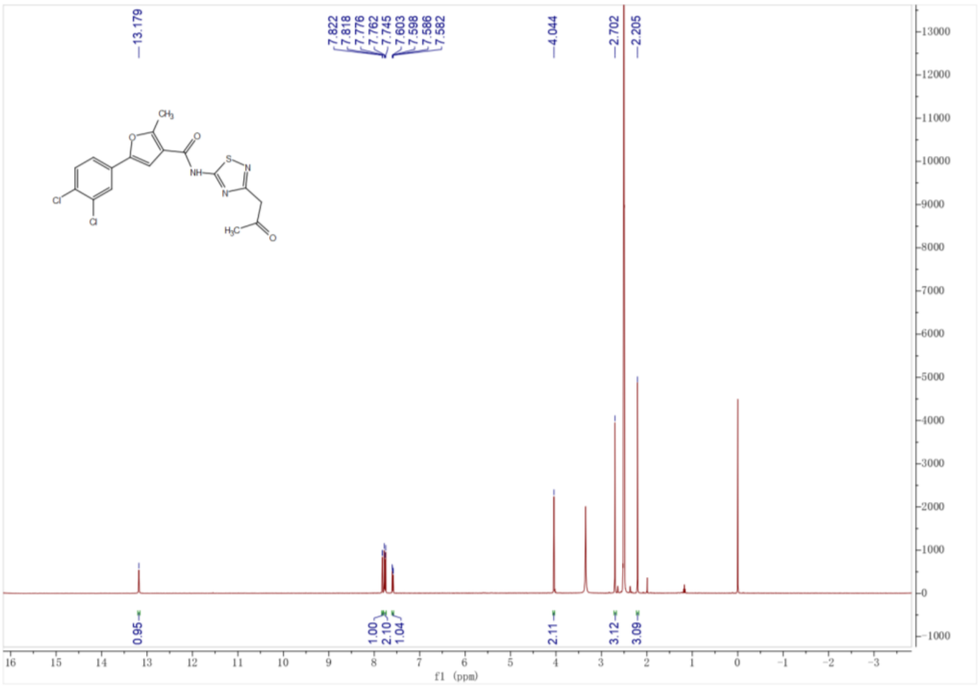

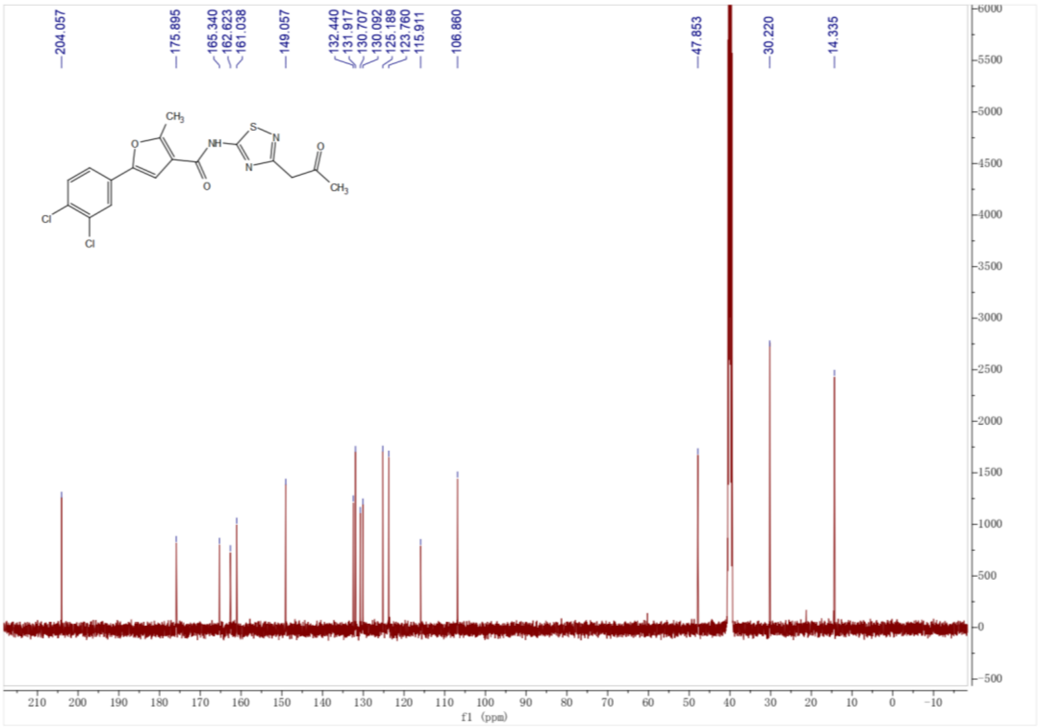

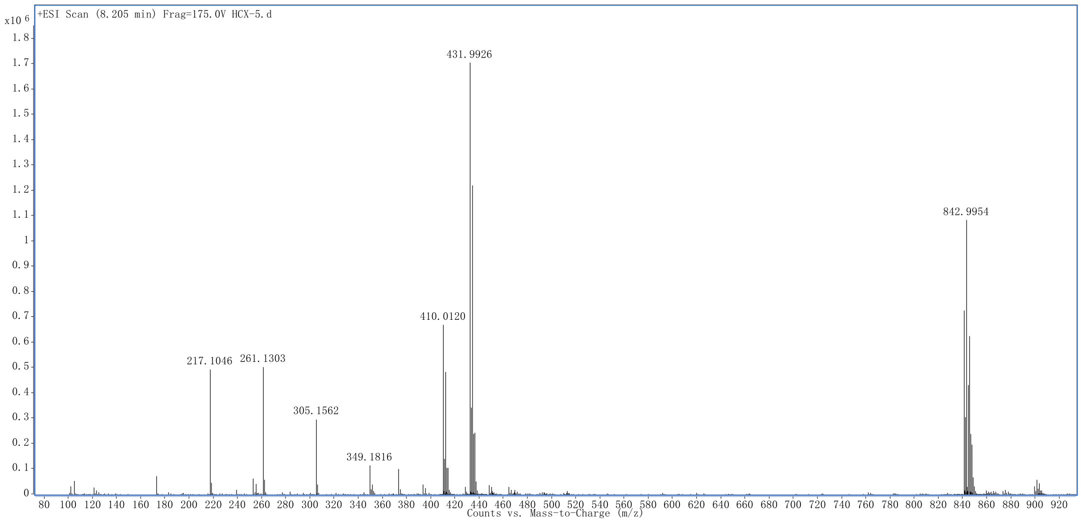

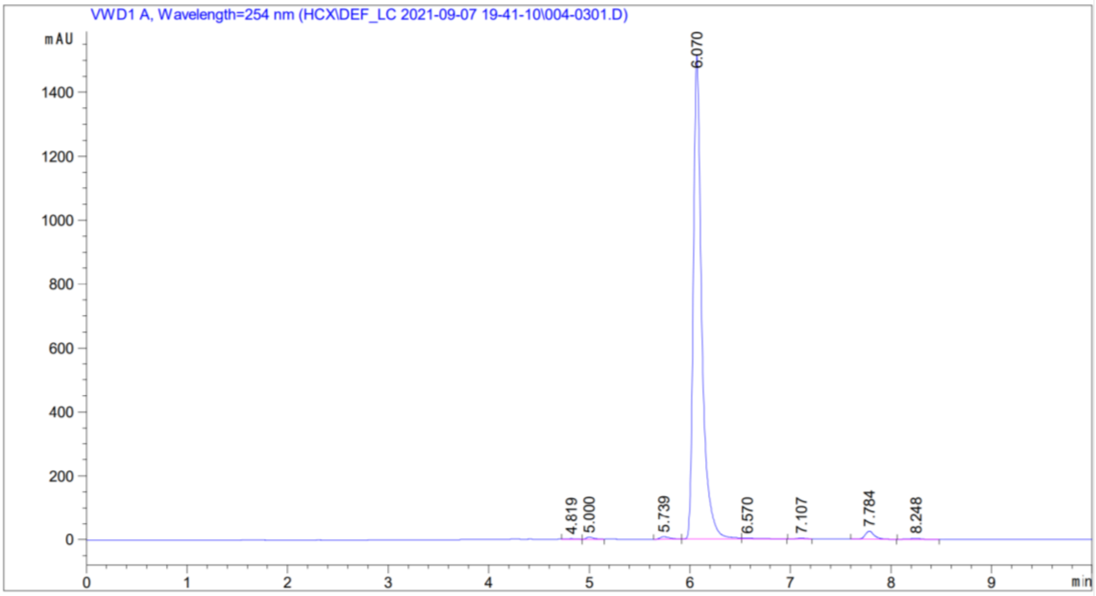

Peak Table

Peak	Ret. Time	Height	Area	Area%
1	4.819	2.2886	17.5015	0.1899
2	5.000	7.3222	40.7117	0.4418
3	5.739	8.4065	54.8866	0.5956
4	6.070	1513.2323	8863.2842	96.1759
5	6.570	3.3628	31.1748	0.3383
6	7.107	2.8600	15.8282	0.1718
7	7.784	25.2716	162.8063	1.7666
8	8.248	3.5839	29.5046	0.3202
Total		1566.3279	9215.6979	100.0000

Compound M17-A2

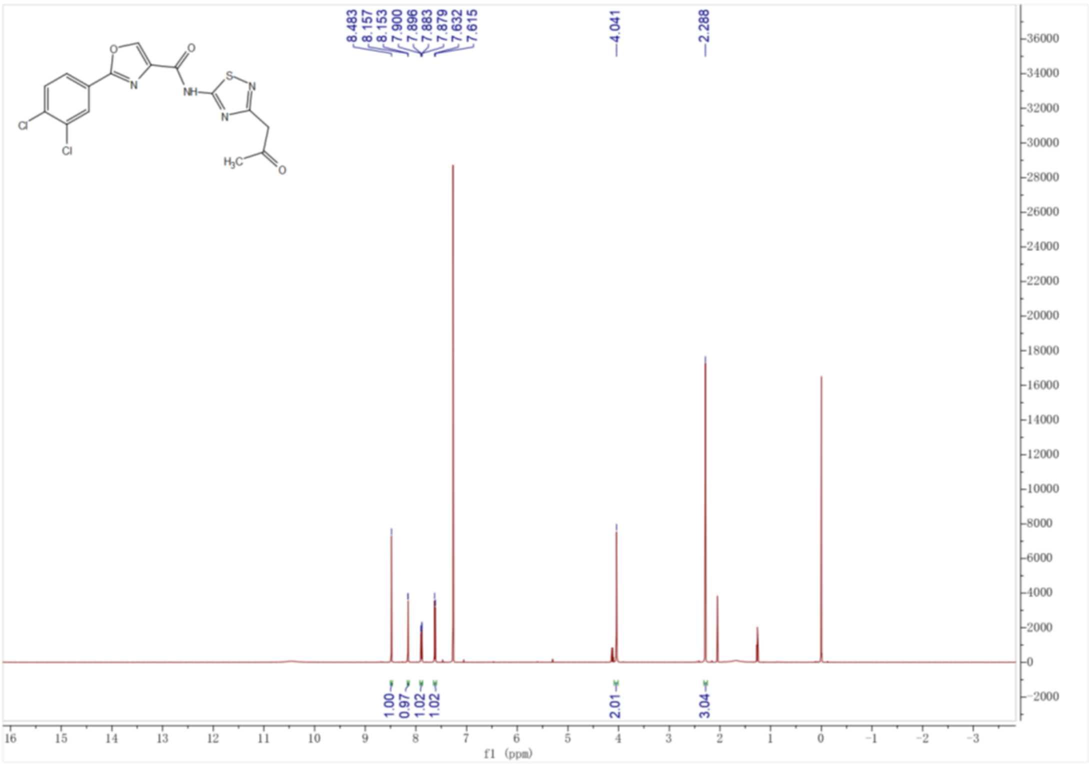

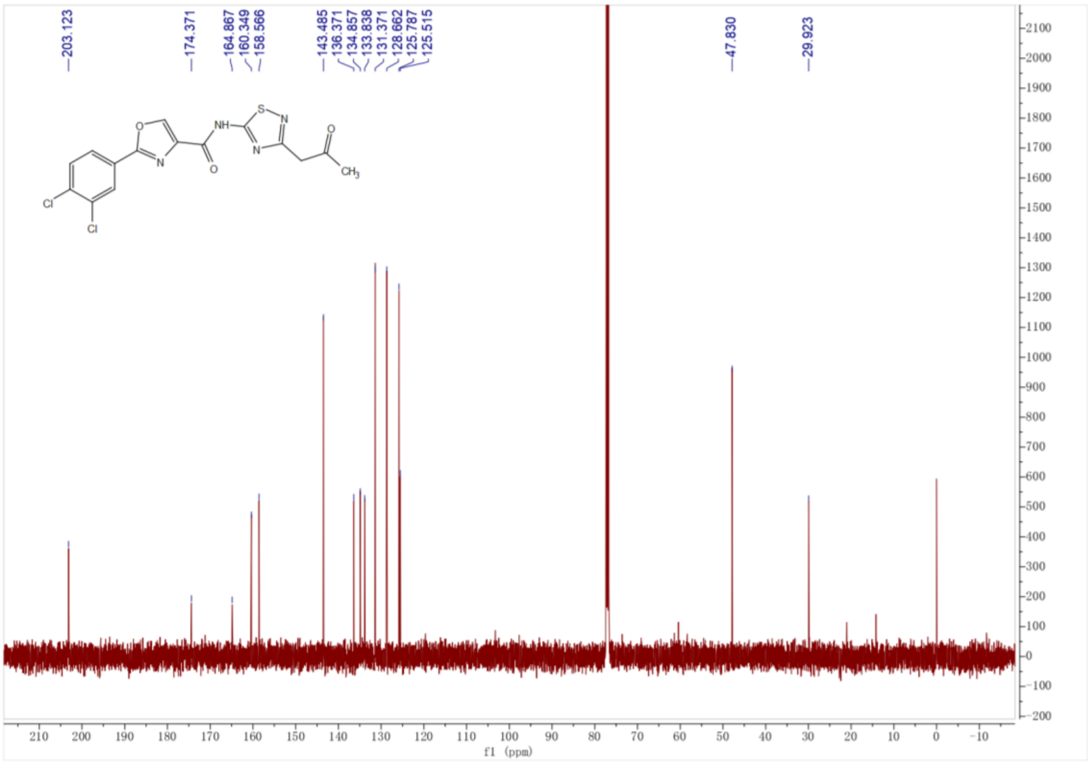

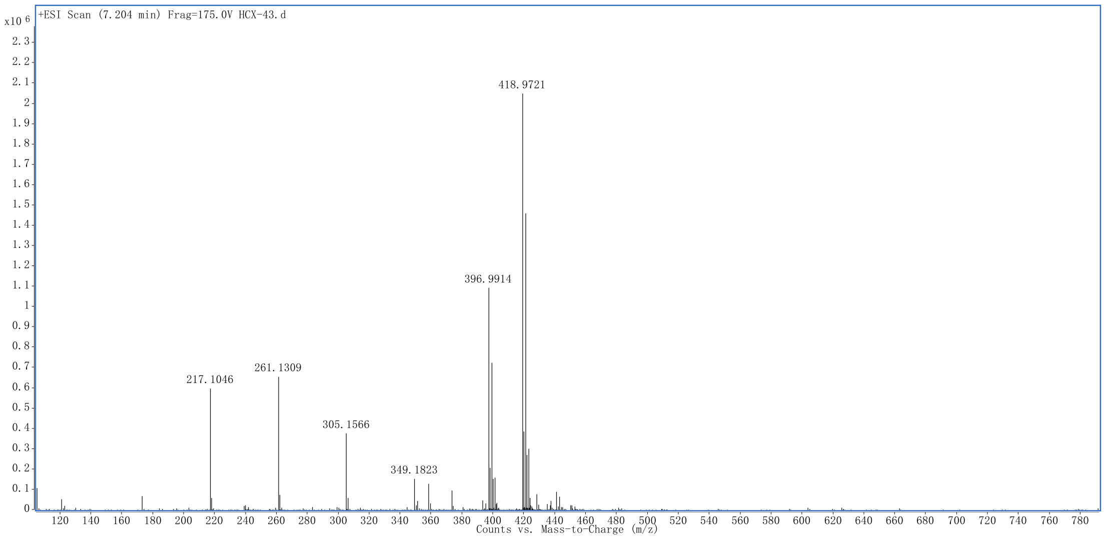

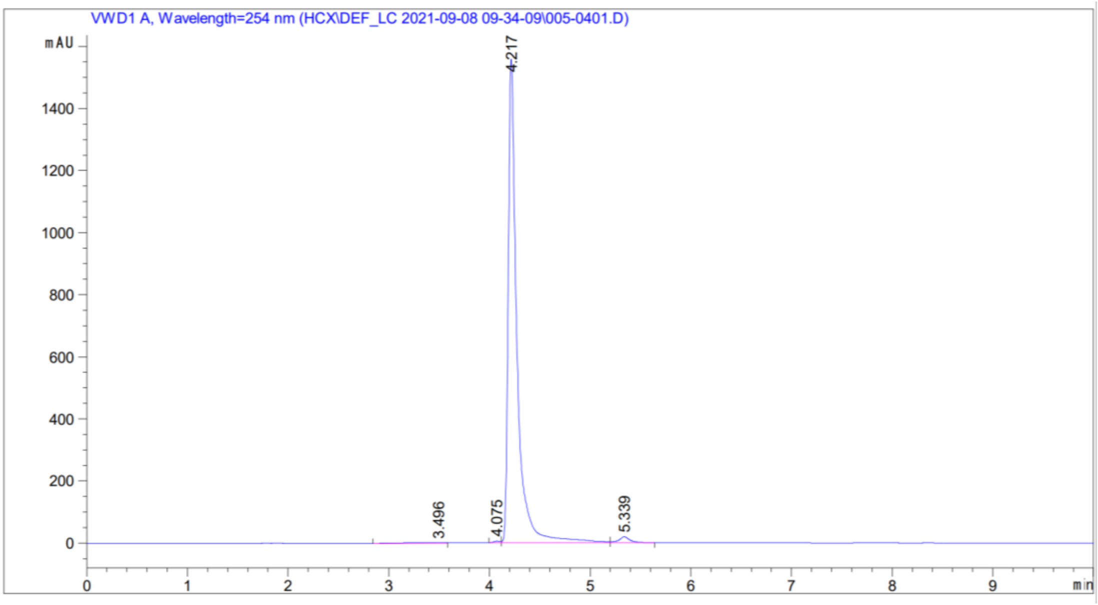

Peak Table

Peak	Ret. Time	Height	Area	Area%
1	3.496	1.8349	39.1732	0.4057
2	4.075	5.0585	21.3741	0.2214
3	4.217	1557.7159	9441.3779	97.7760
4	5.339	19.7147	154.2021	1.5969
Total		1584.3240	9596.1273	100.0000

Compound M17-B1

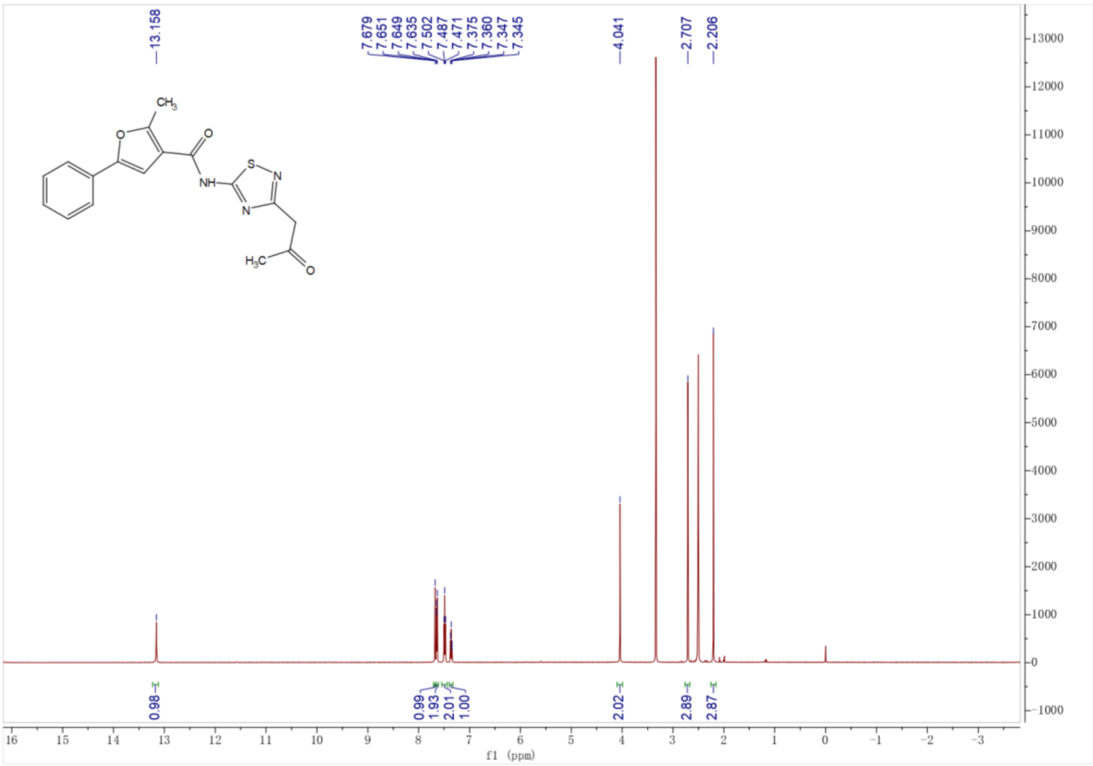

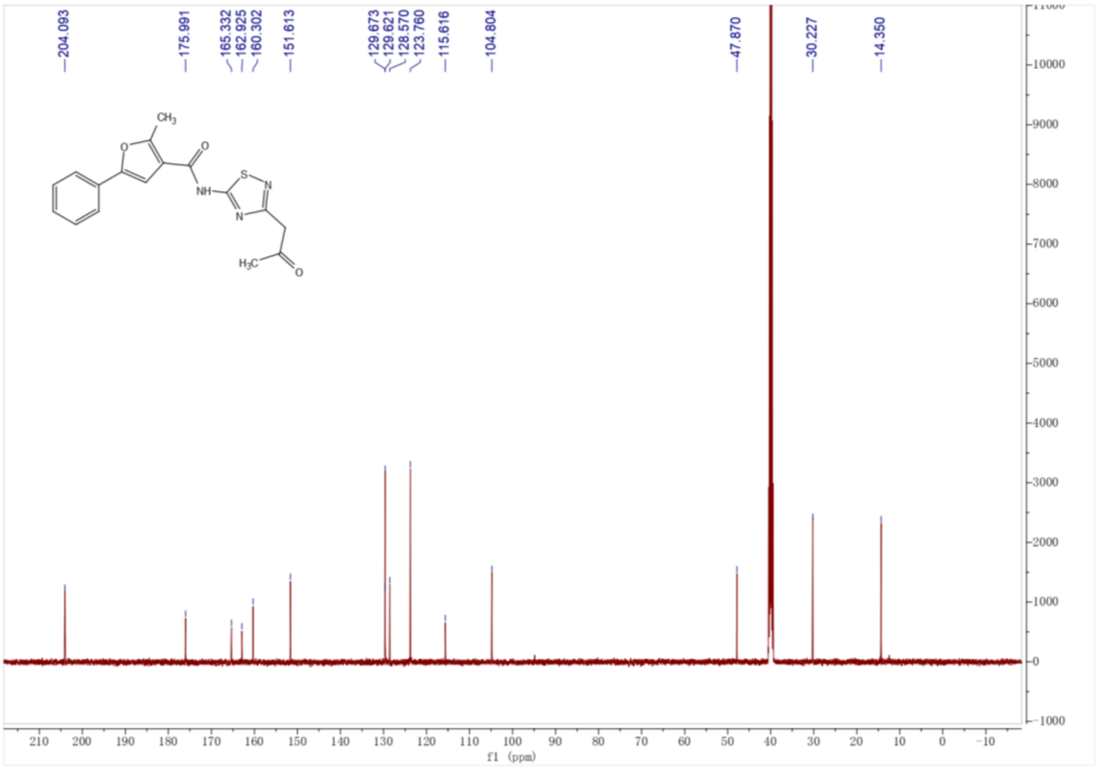

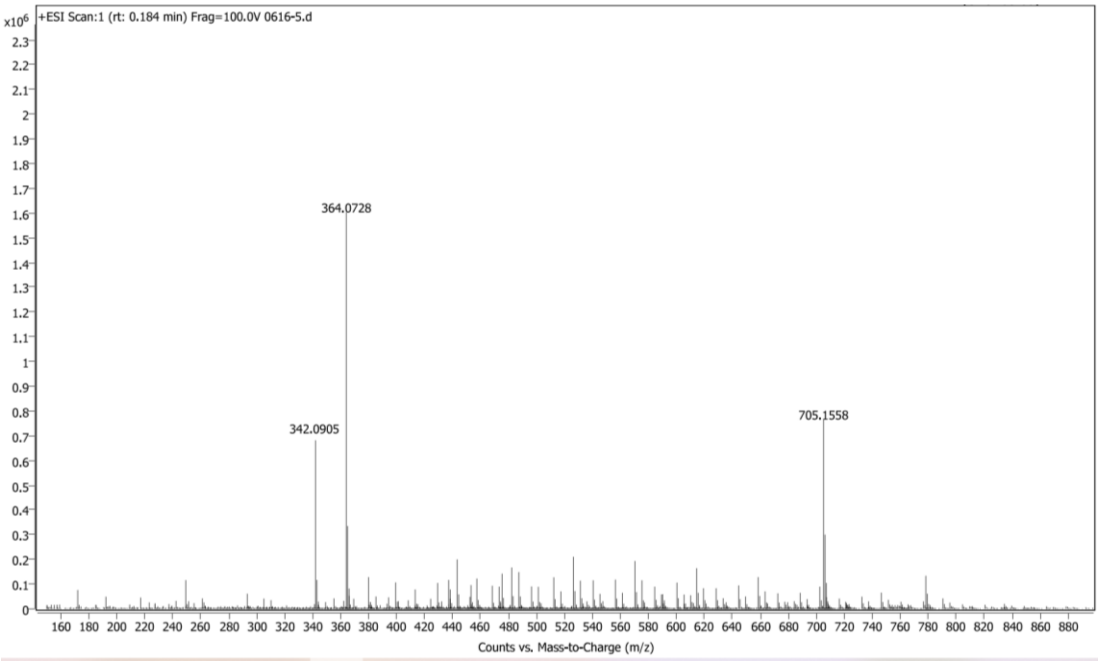

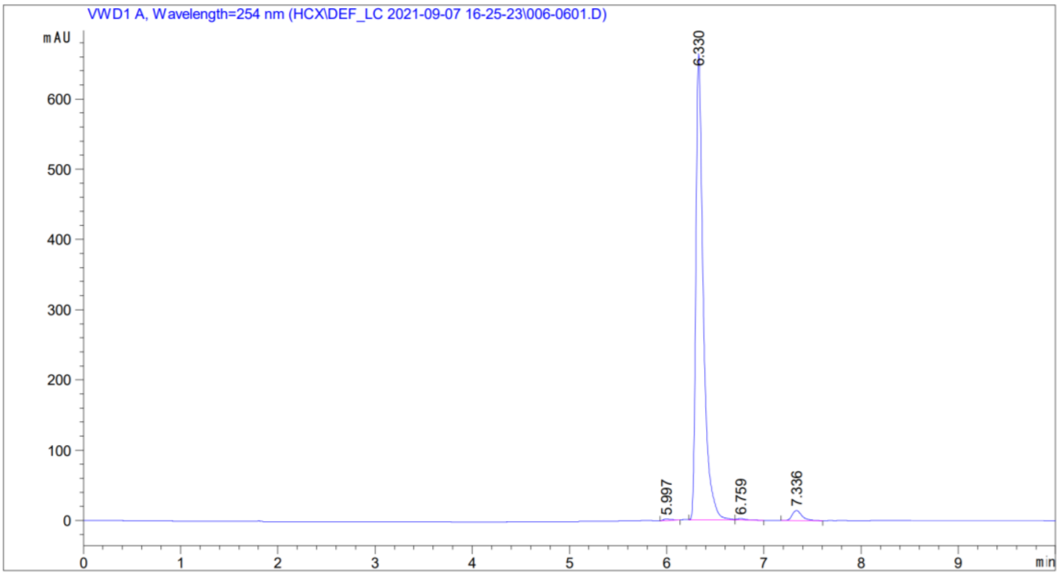

Peak Table

Peak	Ret. Time	Height	Area	Area%
1	5.997	2.2157	11.6164	0.3163
2	6.330	664.1500	3547.2761	96.5814
3	6.759	2.3715	15.3915	0.4191
4	7.336	14.2412	98.5517	2.6833
Total		682.97834	3672.8357	100.0000

Compound M17-B2

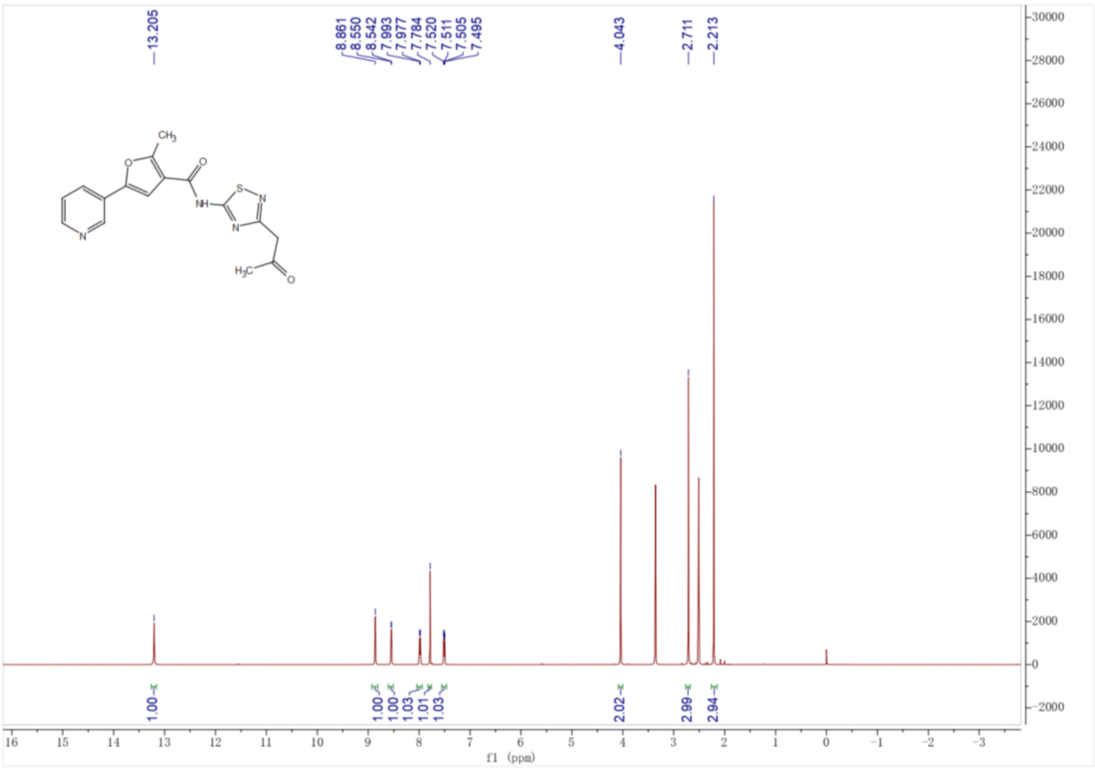

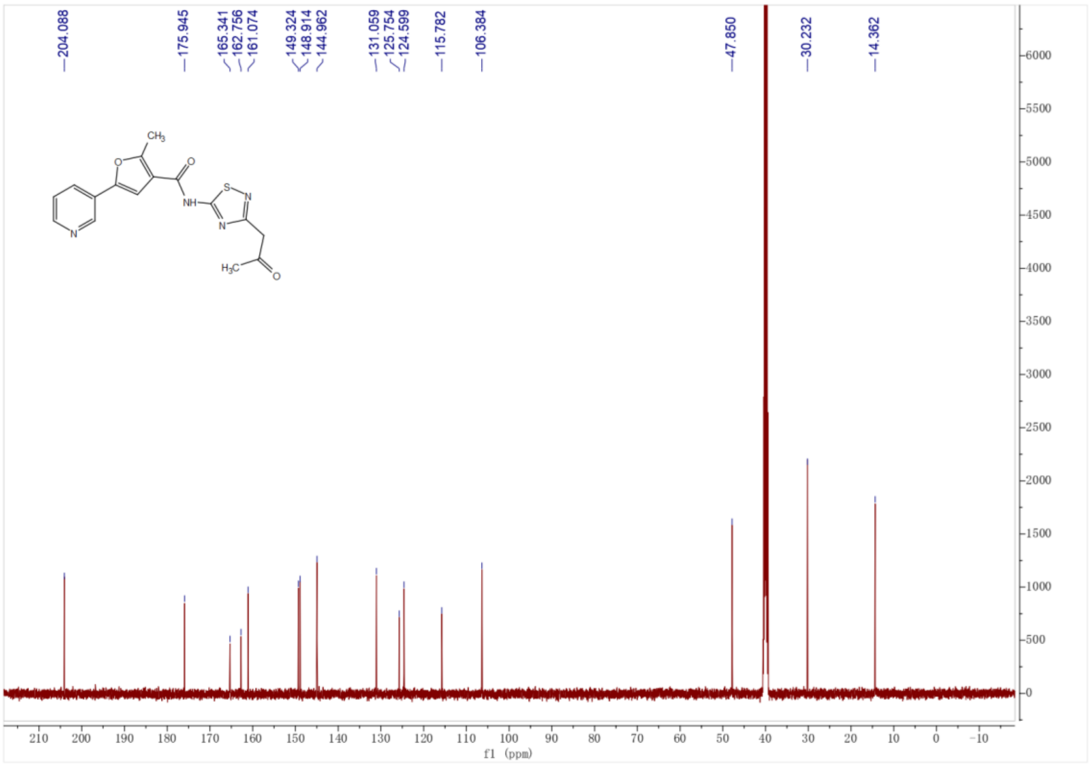

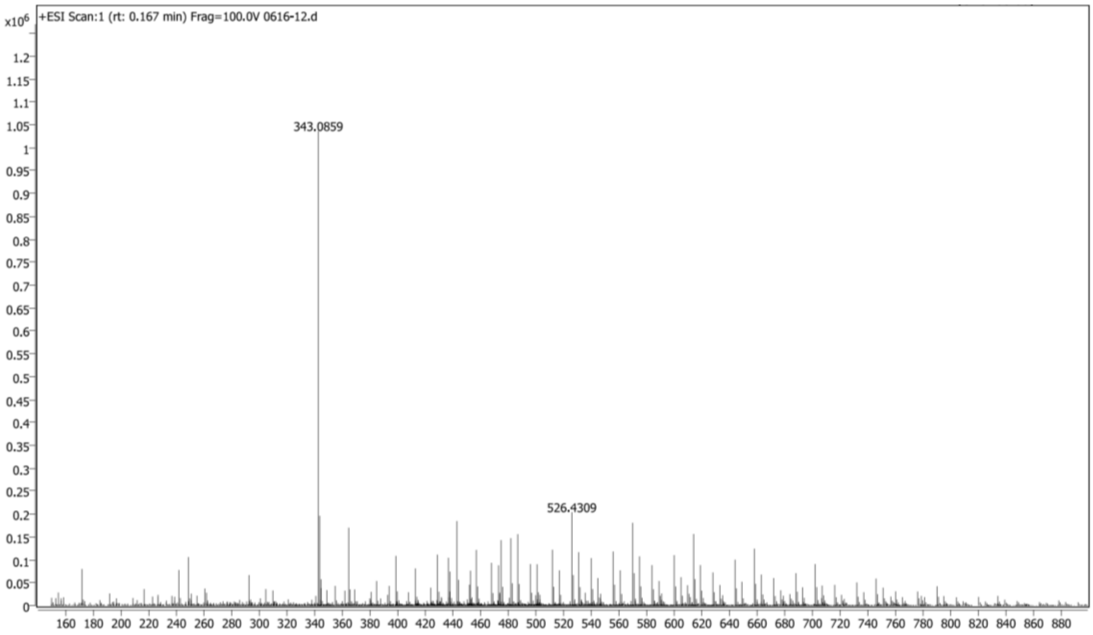

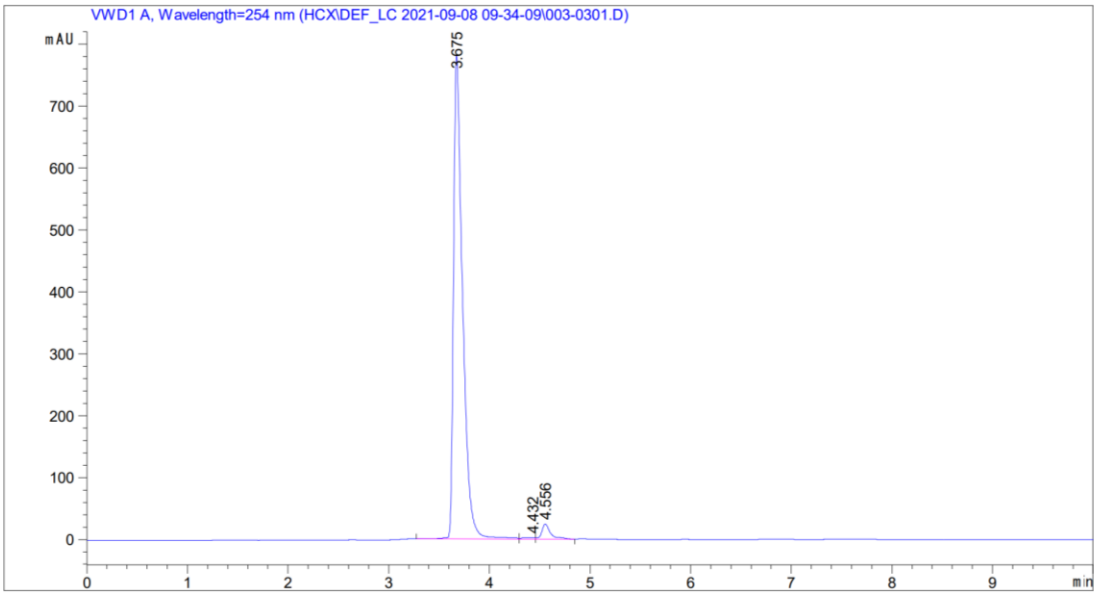

Peak Table

Peak	Ret. Time	Height	Area	Area%
1	3.675	780.9266	4886.8467	96.7376
2	4.432	1.8676	17.1319	0.3391
3	4.556	24.5280	147.6711	2.9232
Total			5051.6497	100.0000

Compound M17-B3

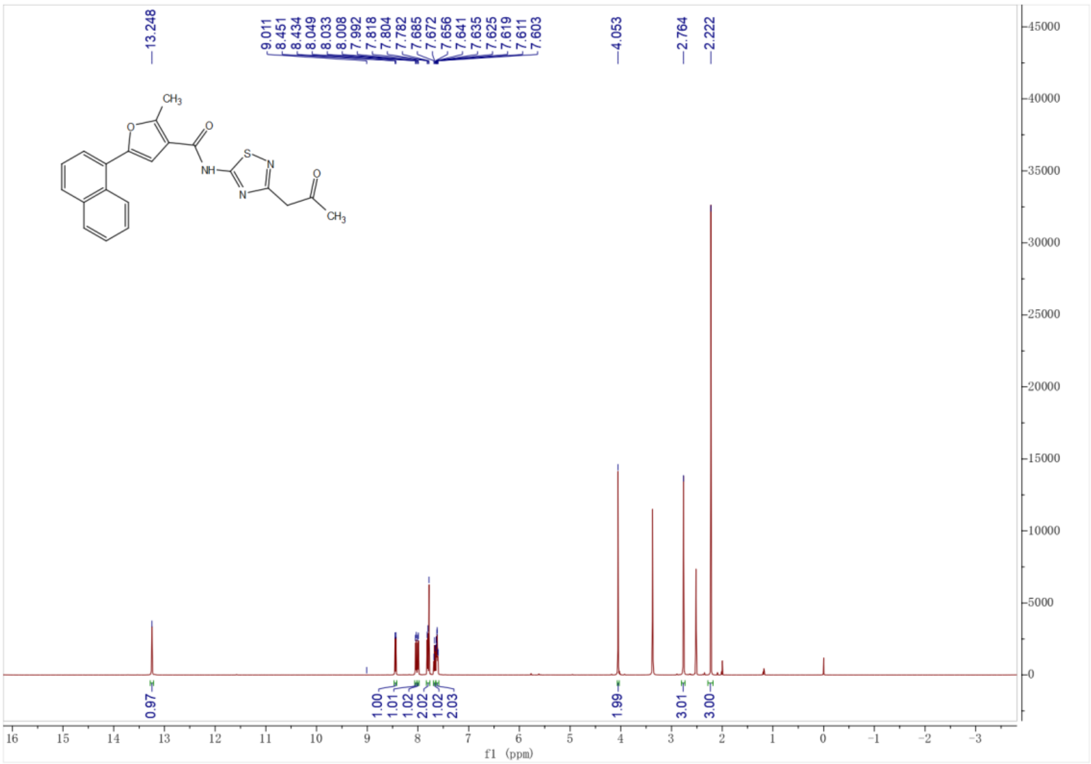

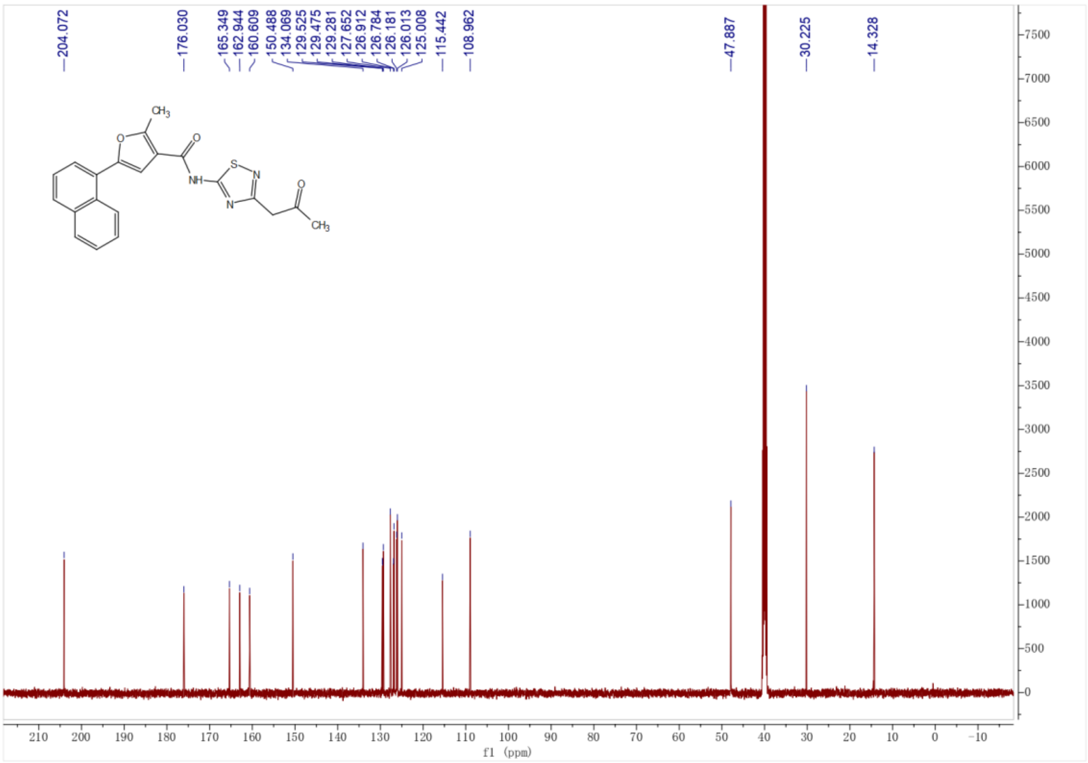

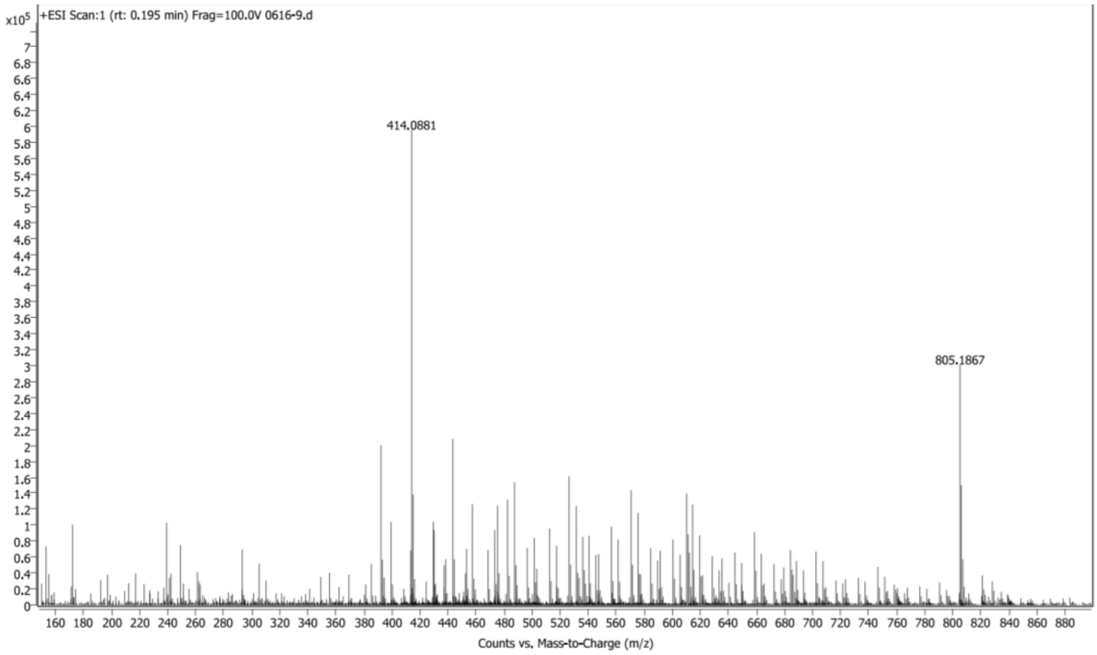

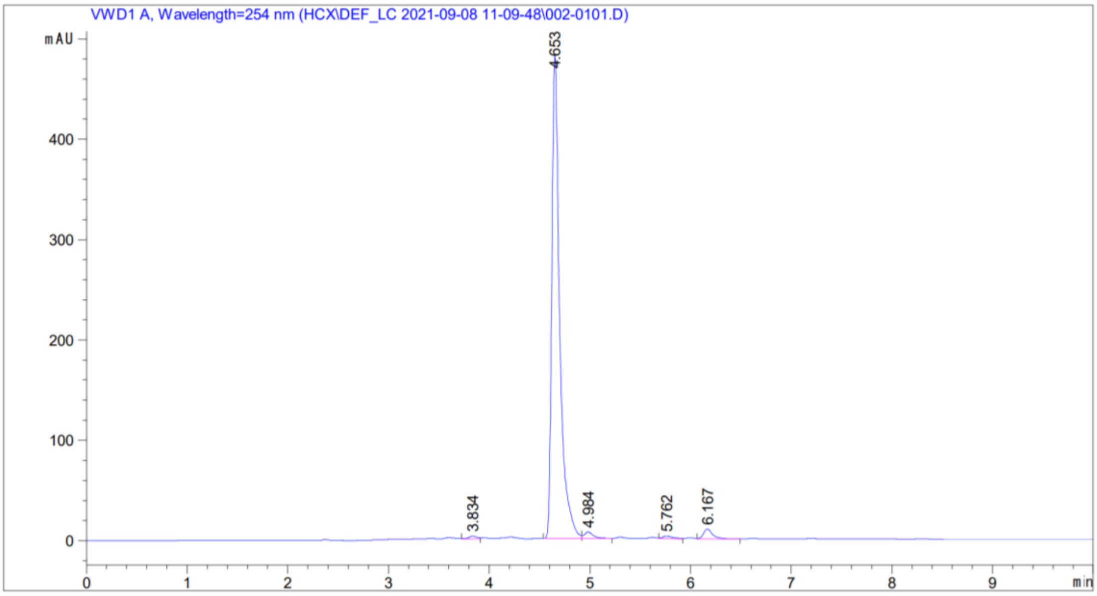

Peak Table

Peak	Ret. Time	Height	Area	Area%
1	3.834	2.7911	16.1240	0.5870
2	4.653	481.4985	2611.2822	95.0565
3	4.984	6.4607	40.6455	1.4796
4	5.762	2.6790	18.6497	0.6789
5	6.167	9.7454	60.3840	2.1981
Total		503.17470	2747.0855	100.0000

Compound M17-B4

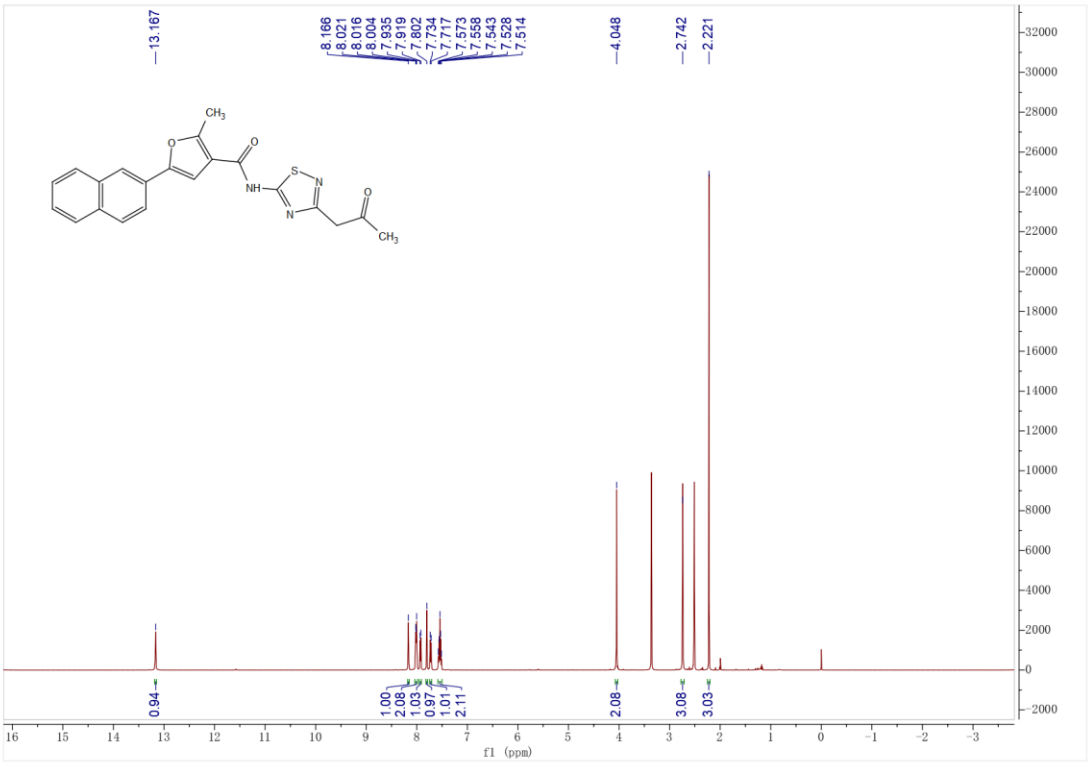

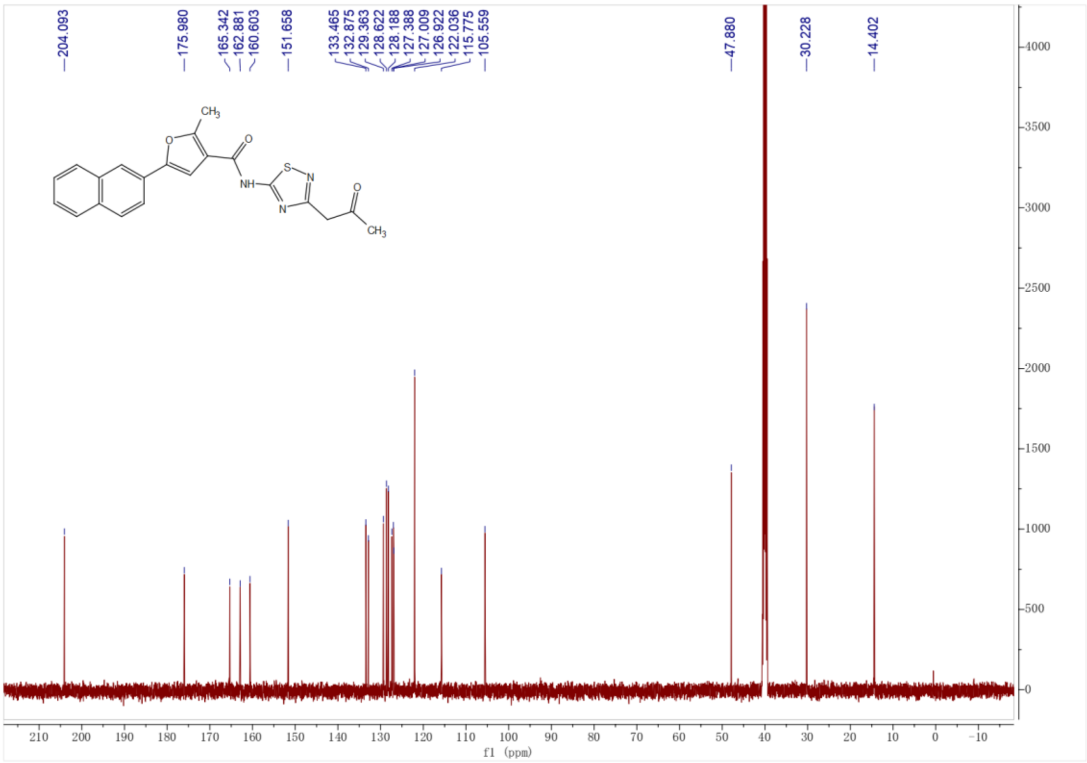

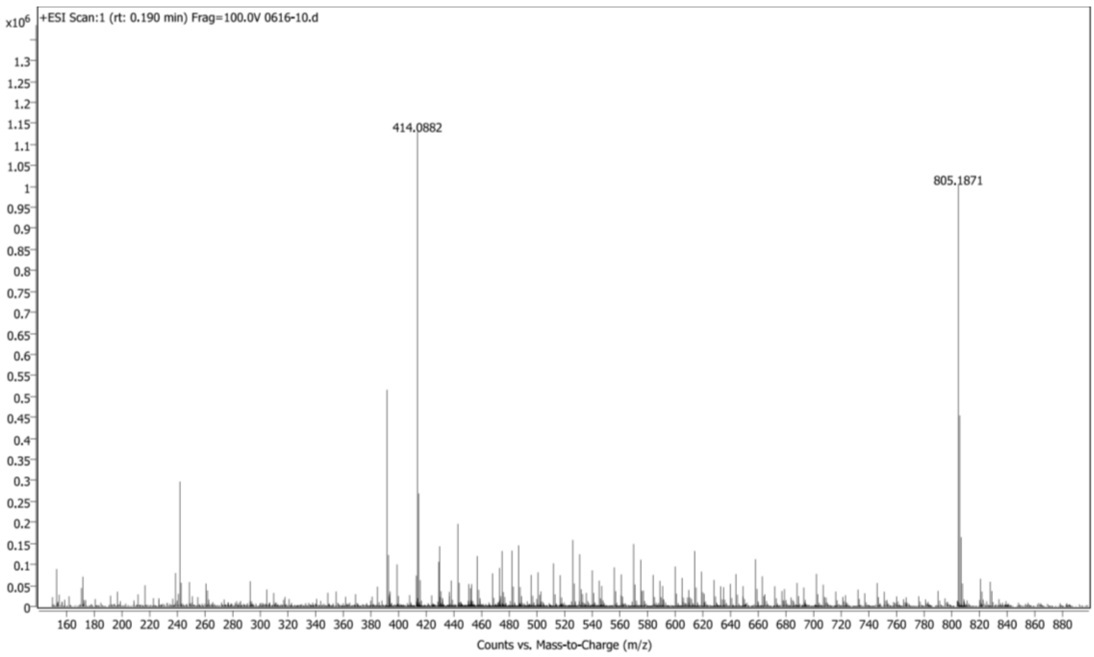

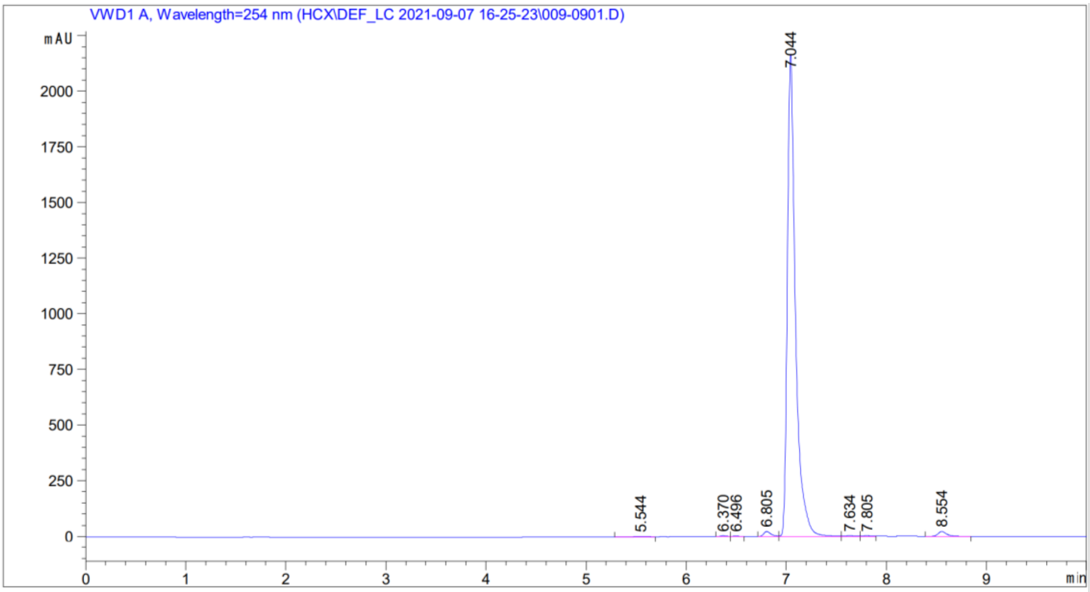

Peak Table

Peak	Ret. Time	Height	Area	Area%
1	5.544	1.9858	14.6436	0.1168
2	6.370	4.0410	18.1434	0.1447
3	6.496	2.4867	10.5293	0.0839
4	6.805	22.4933	116.1597	0.9261
5	7.044	2161.3162	12188.9	97.1794
6	7.634	3.2129	26.2521	0.2093
7	7.805	3.1558	20.1489	0.1606
8	8.554	22.6361	147.9044	1.1792
Total		2221.3298	12542.7	100.0000

Compound M17-B10

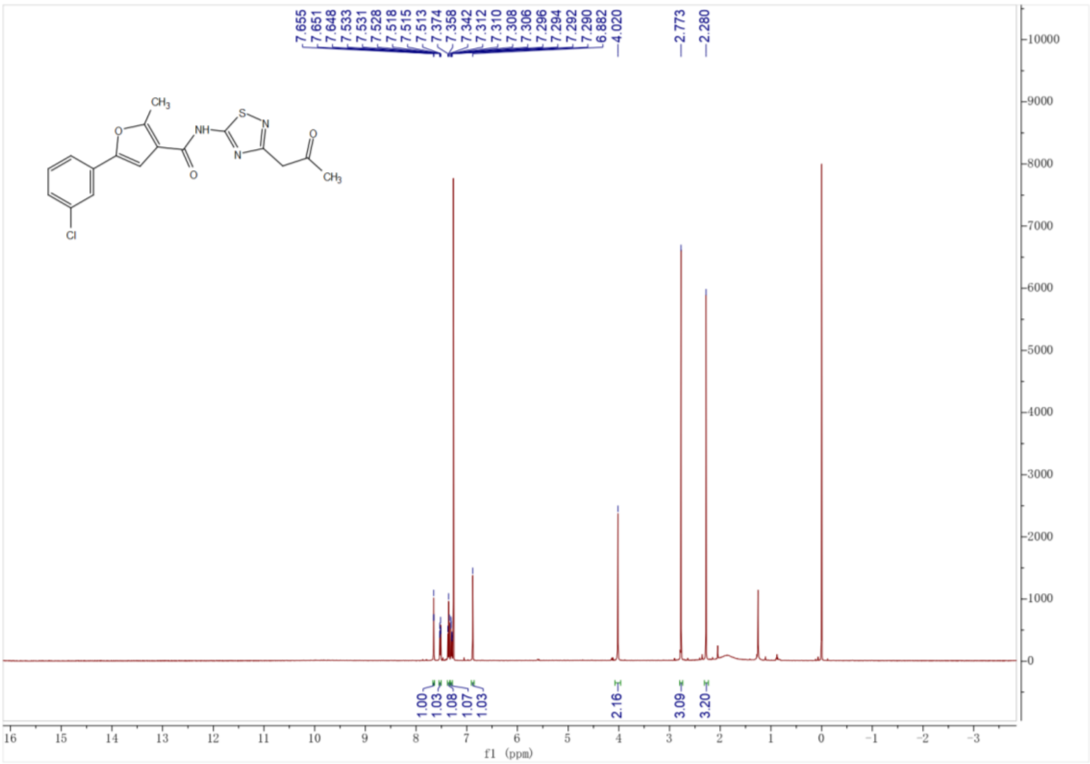

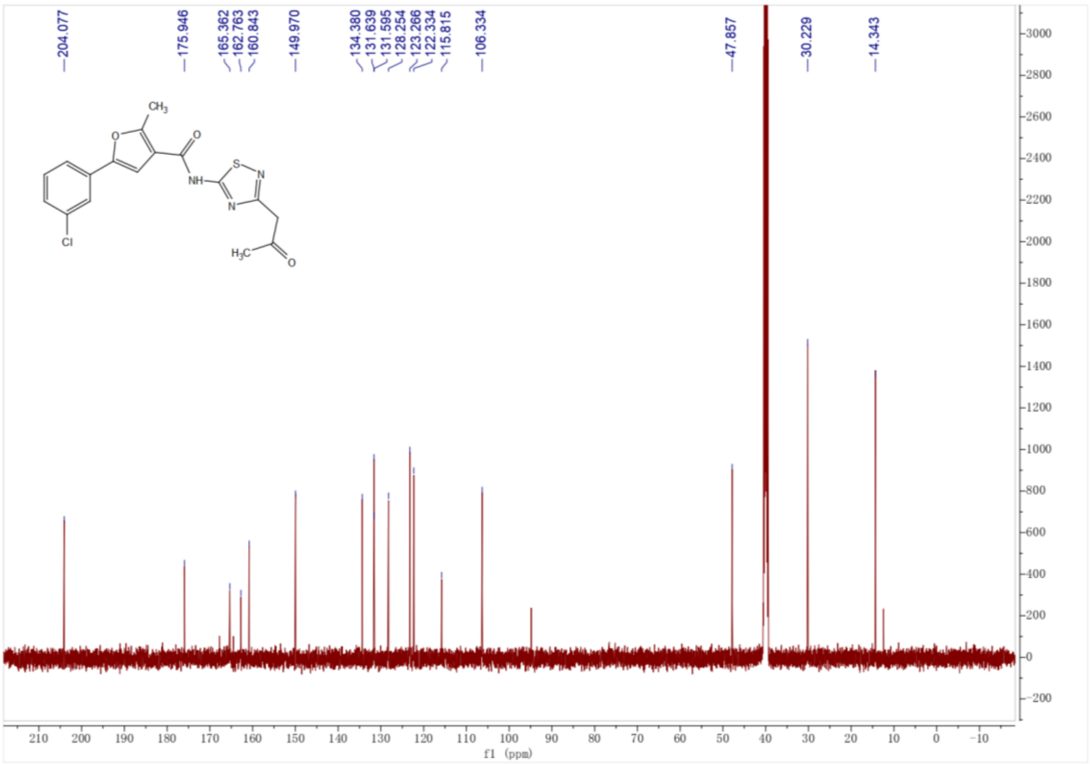

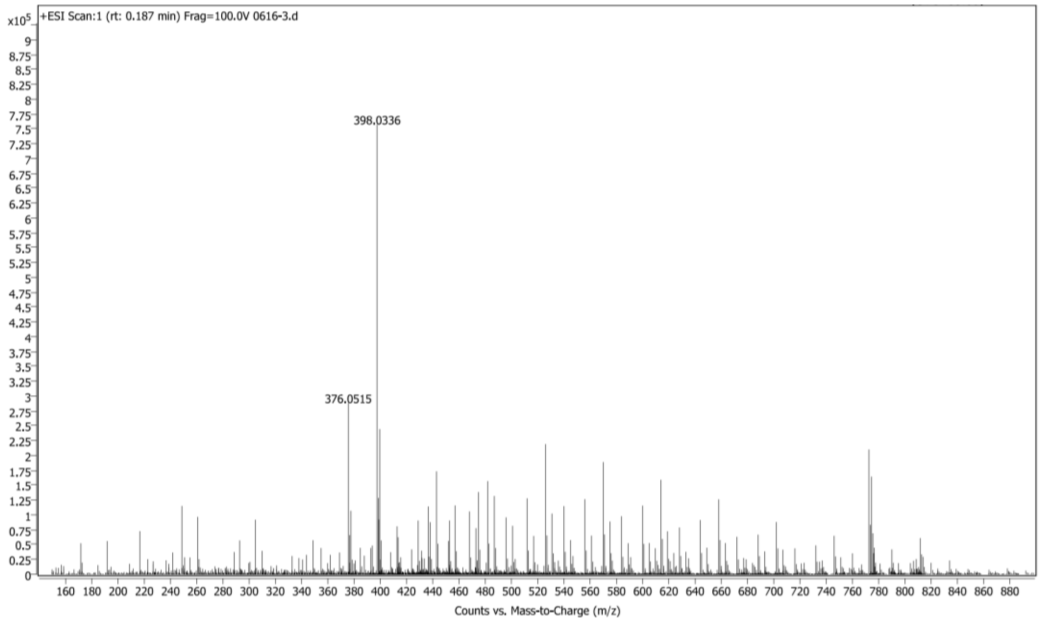

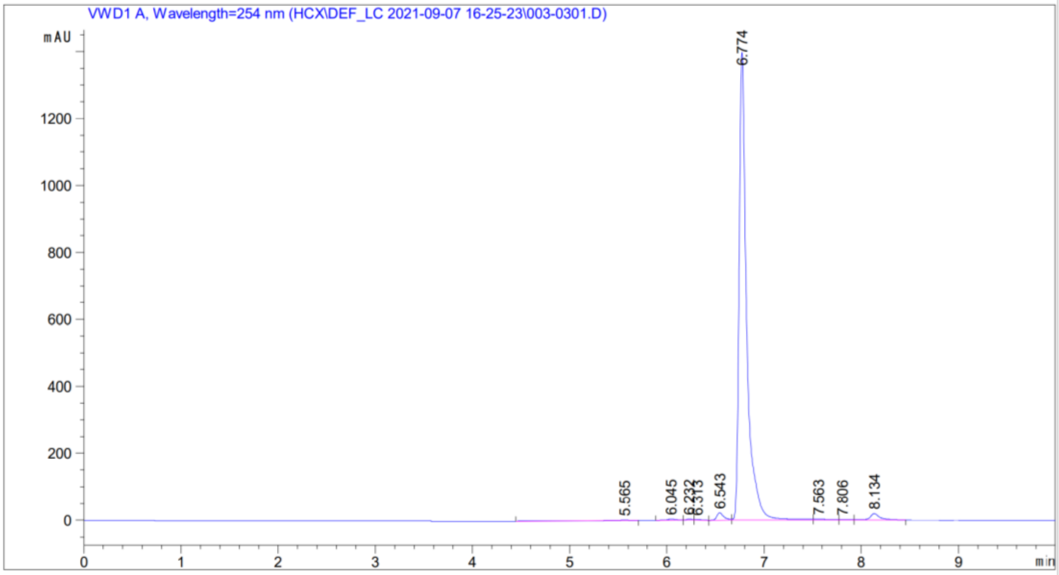

Peak Table

Peak	Ret. Time	Height	Area	Area%
1	5.565	1.7856	25.0827	0.3135
2	6.045	3.9748	27.4118	0.3426
3	6.232	3.3797	13.0178	0.1627
4	6.313	2.2996	10.3813	0.1298
5	6.543	22.1183	111.5621	1.1298
6	6.774	1395.4038	7619.9248	95.2493
7	7.563	2.4522	34.2988	0.4287
8	7.806	1.8437	16.1118	0.2014
9	8.134	19.3450	142.1925	1.7774
Total		1452.6027	7999.9837	100.0000

Compound M17-B12

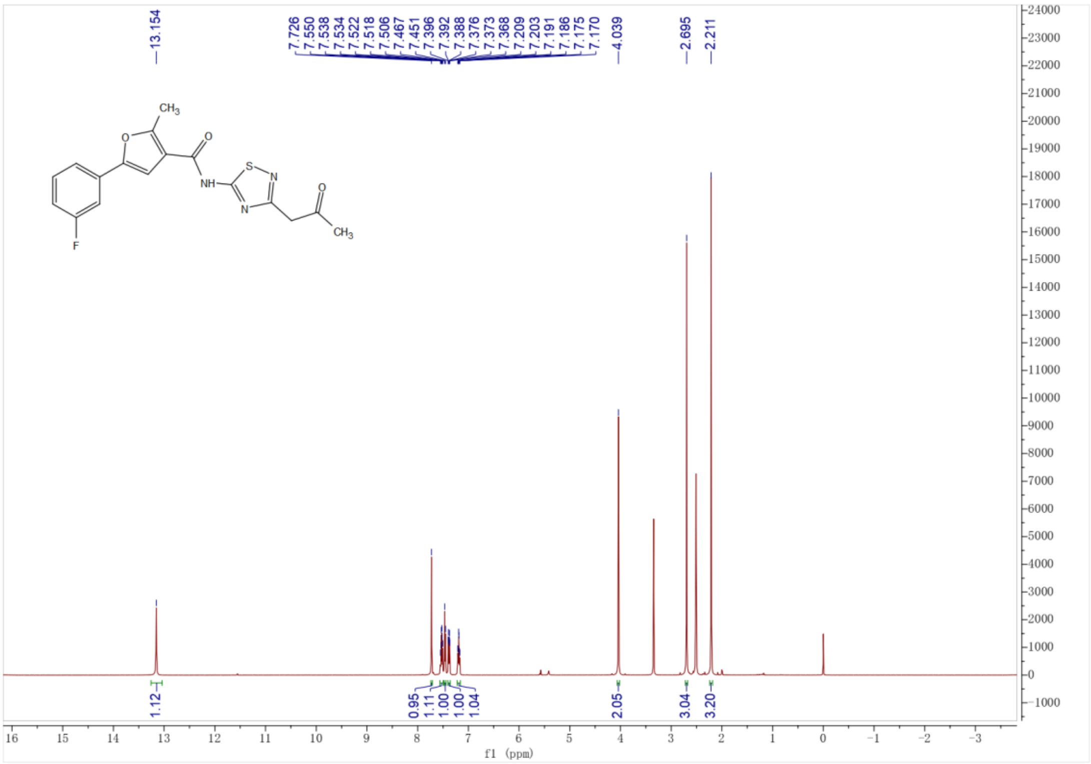

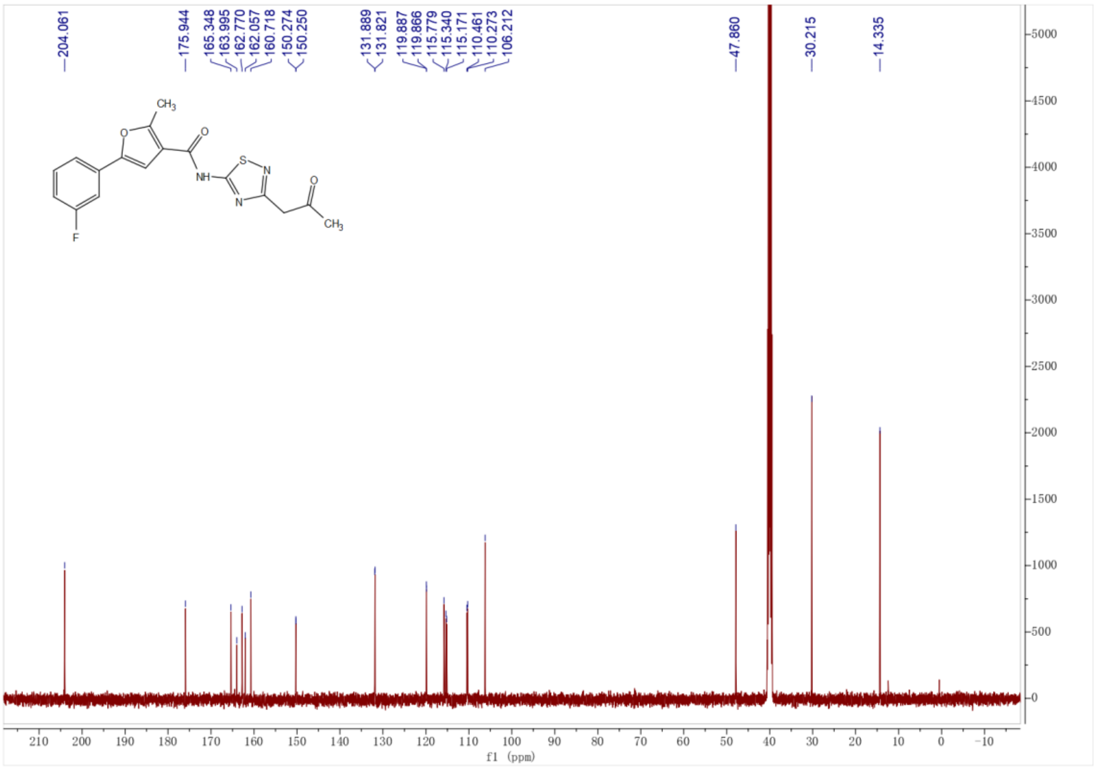

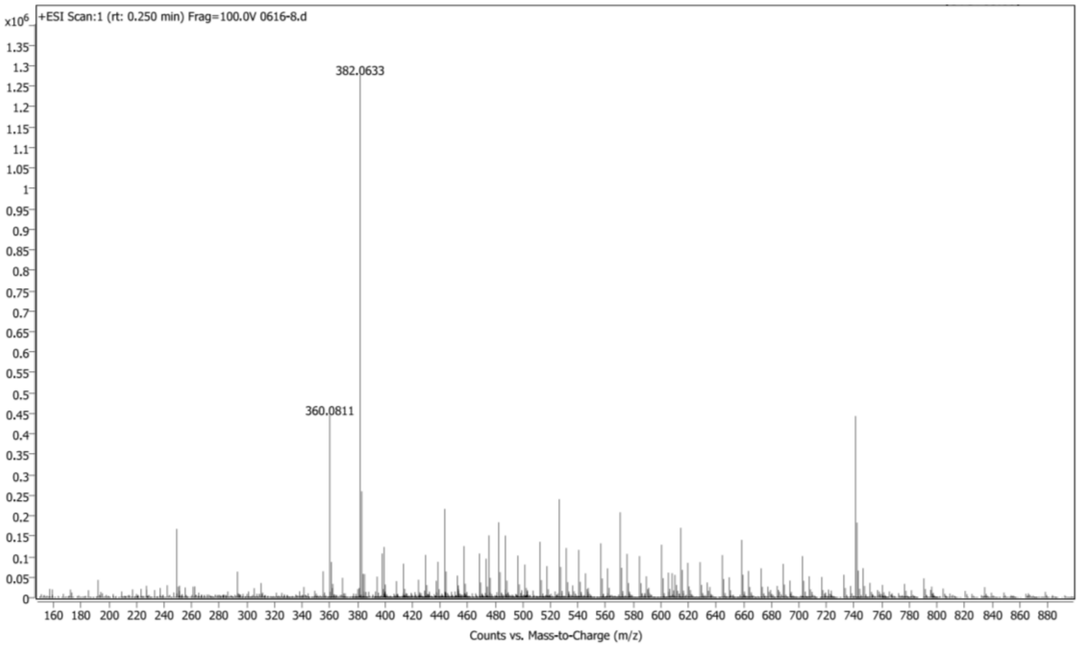

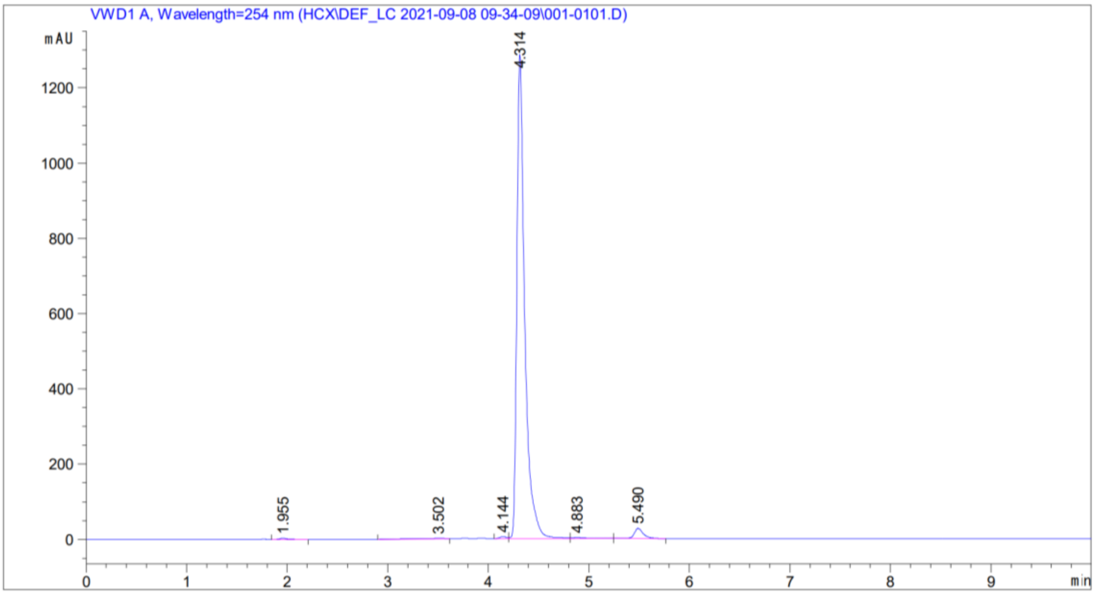

Peak Table

Peak	Ret. Time	Height	Area	Area%
1	1.955	3.5140	15.3704	0.2115
2	3.502	1.8300	37.7755	0.5196
3	4.144	5.0138	23.2452	0.3197
4	4.314	1284.5282	6997.4707	96.2487
5	4.883	3.0139	27.3672	0.3764
6	5.490	27.0792	168.9694	2.3241
Total		1324.9790	7270.2012	100.0000

Compound M17-B13

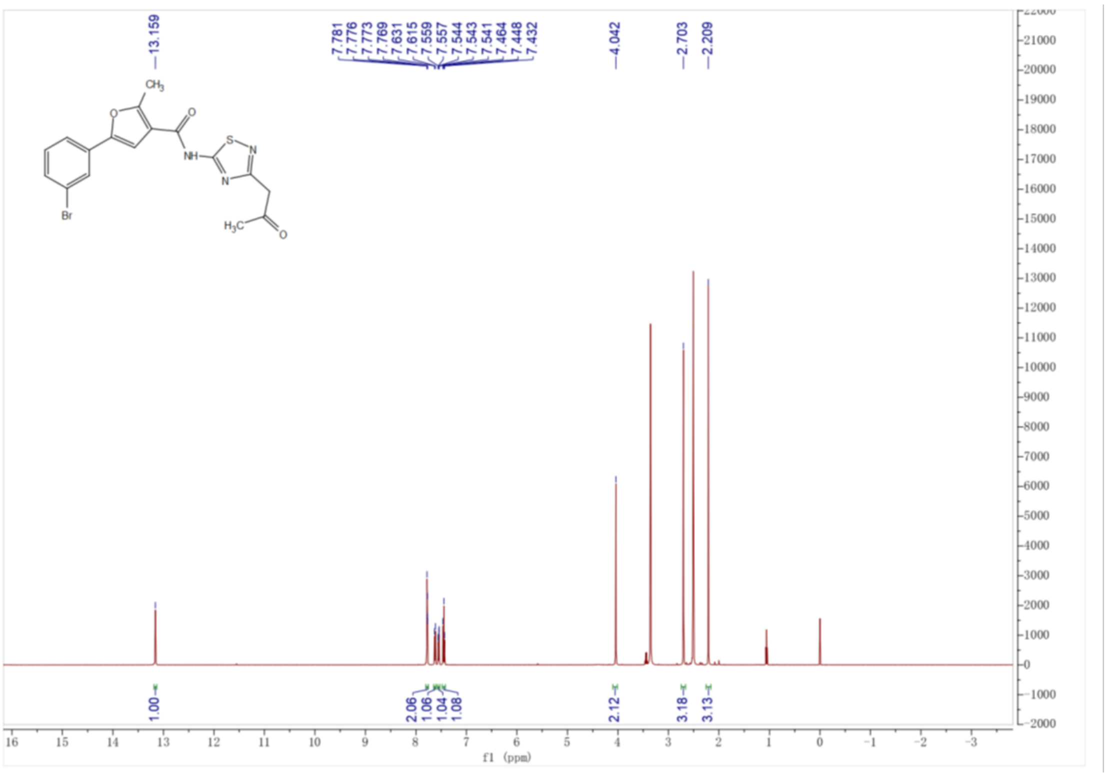

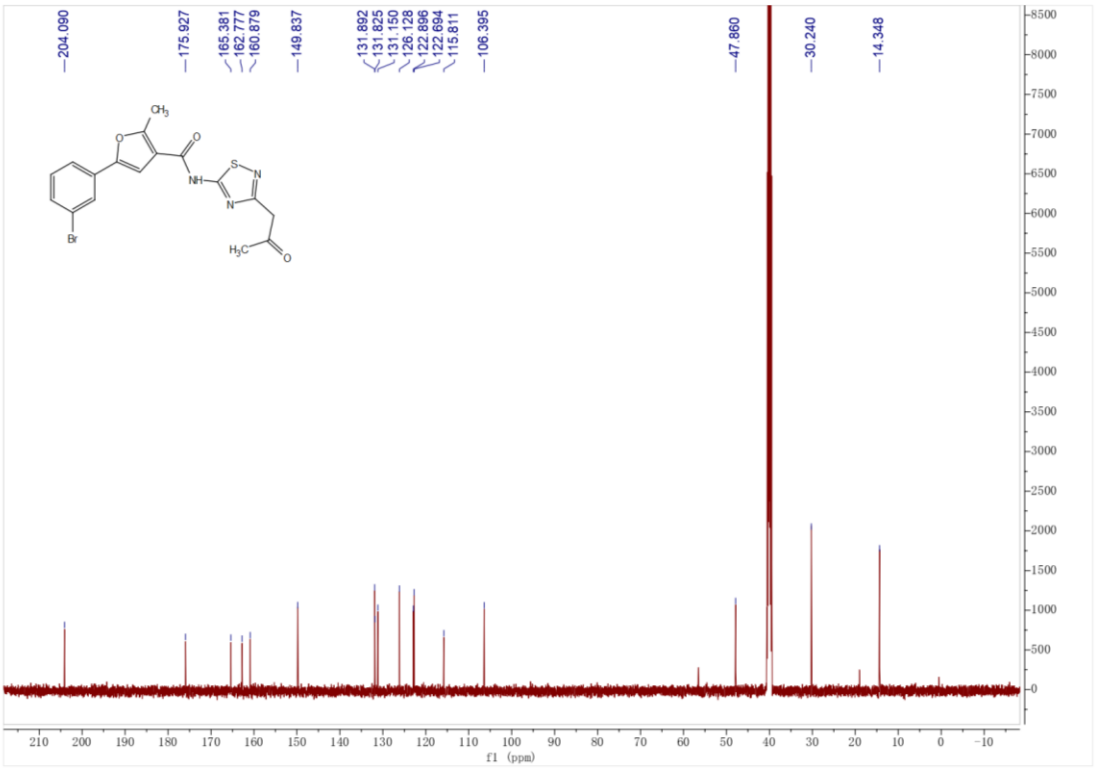

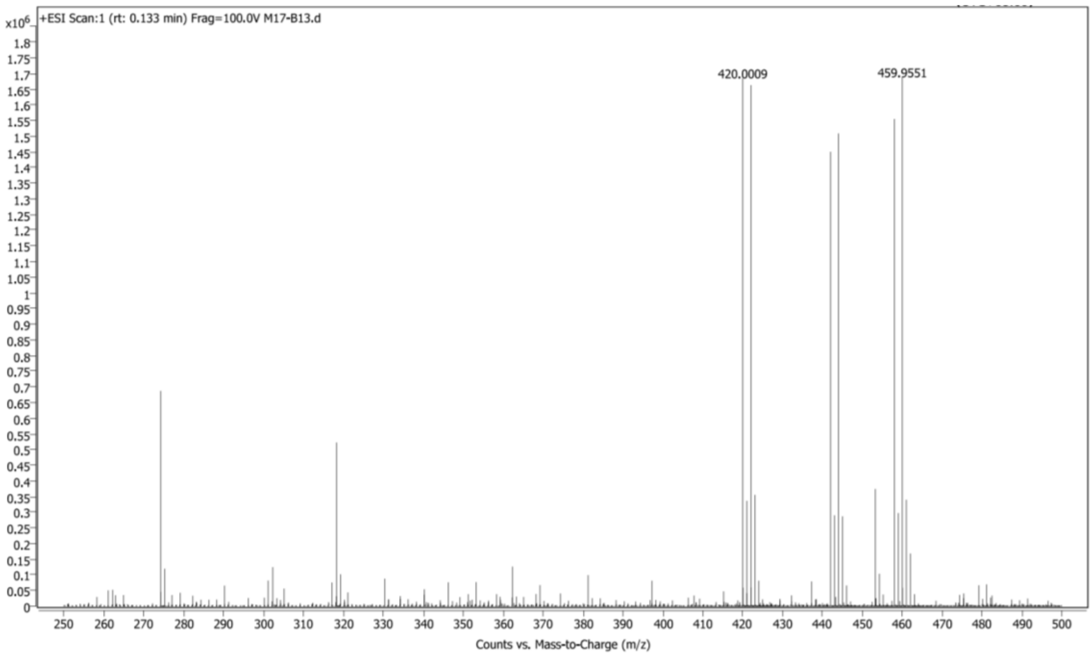

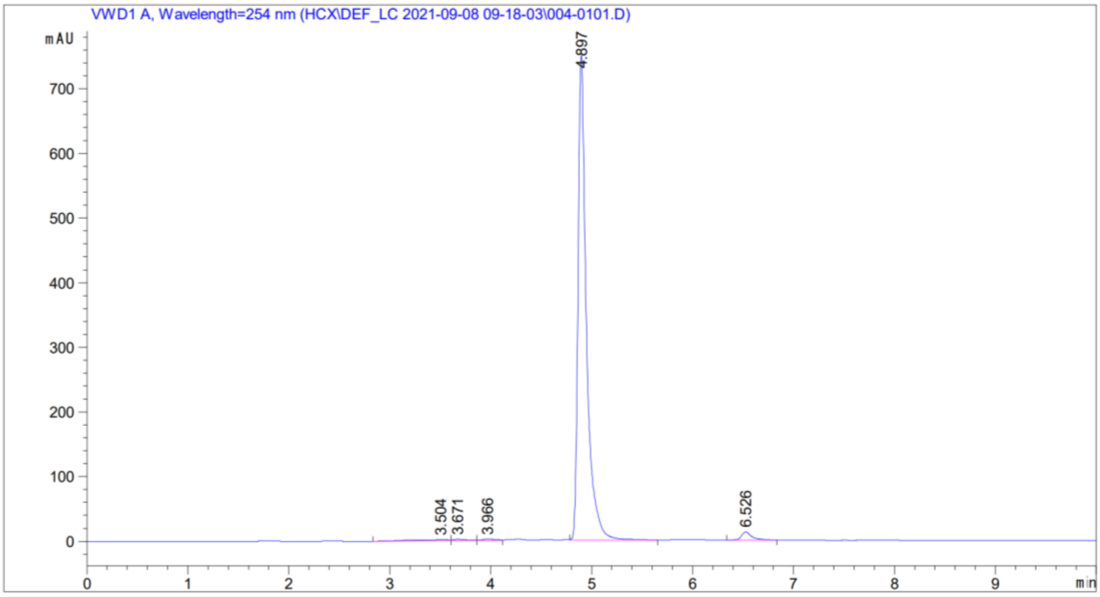

Peak Table

Peak	Ret. Time	Height	Area	Area%
1	3.504	1.9832	50.9201	1.1296
2	3.671	2.1316	21.0961	0.4680
3	3.966	2.3210	20.7303	0.4599
4	4.897	749.9524	4329.1636	96.0332
5	6.526	12.3716	86.0754	1.9094
Total		768.7598	4507.9855	100.0000

Compound M17-B14

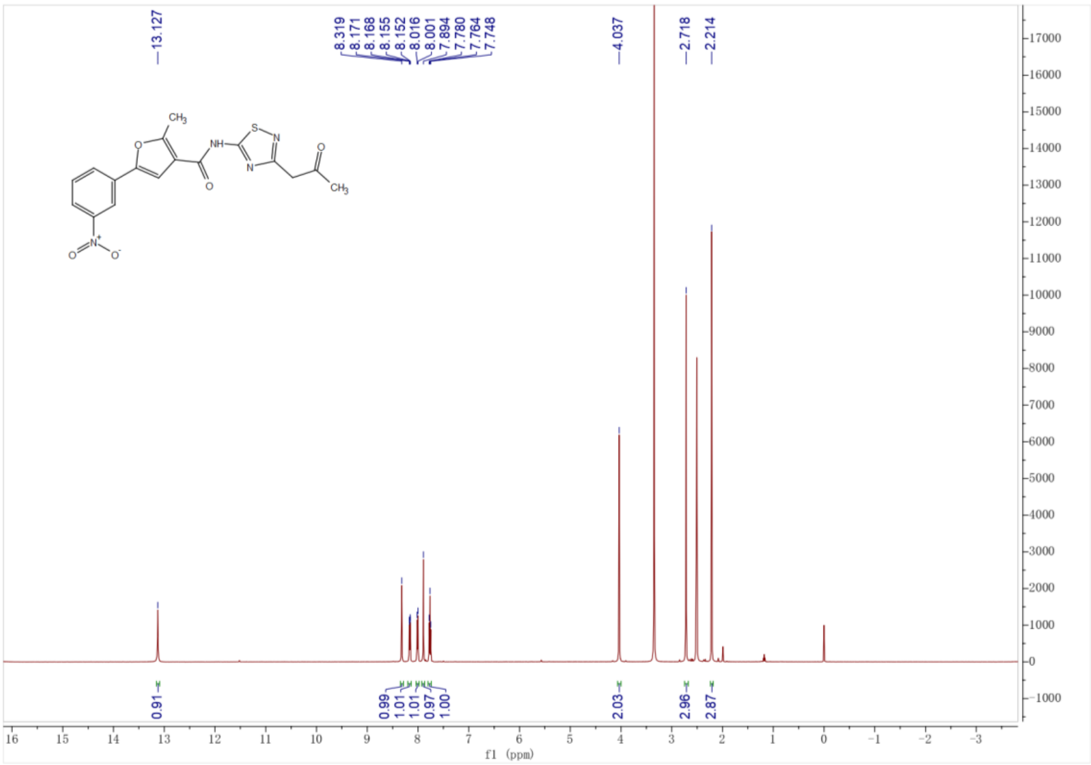

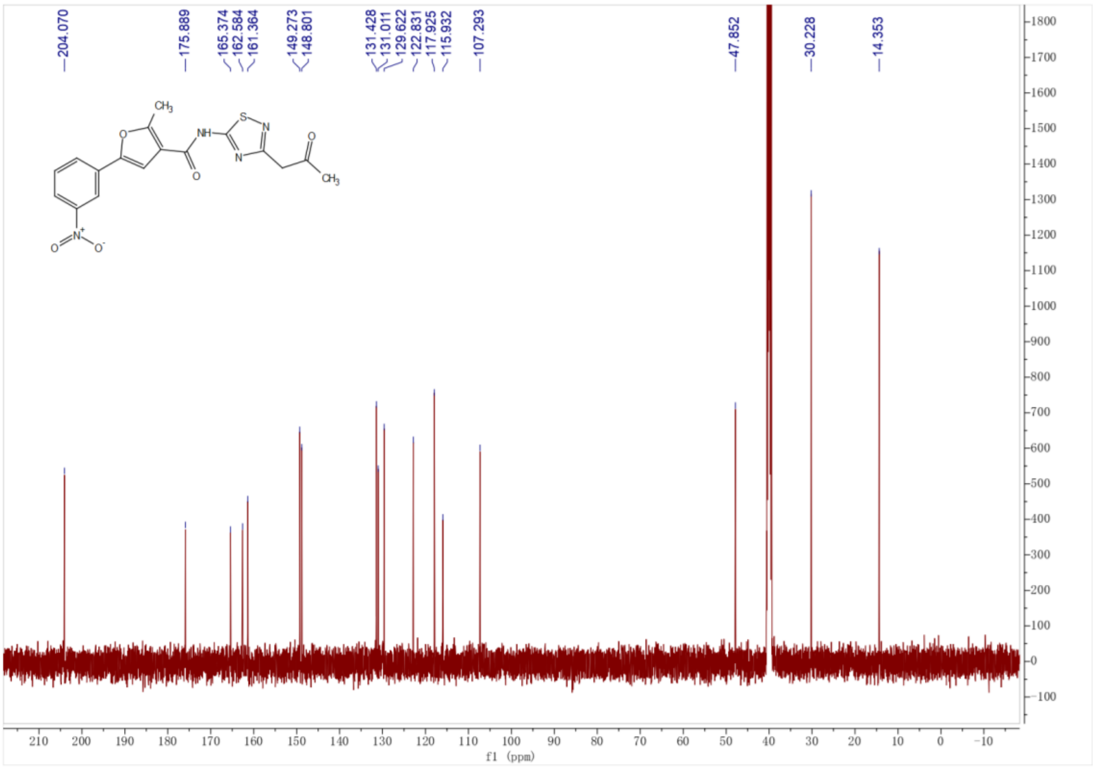

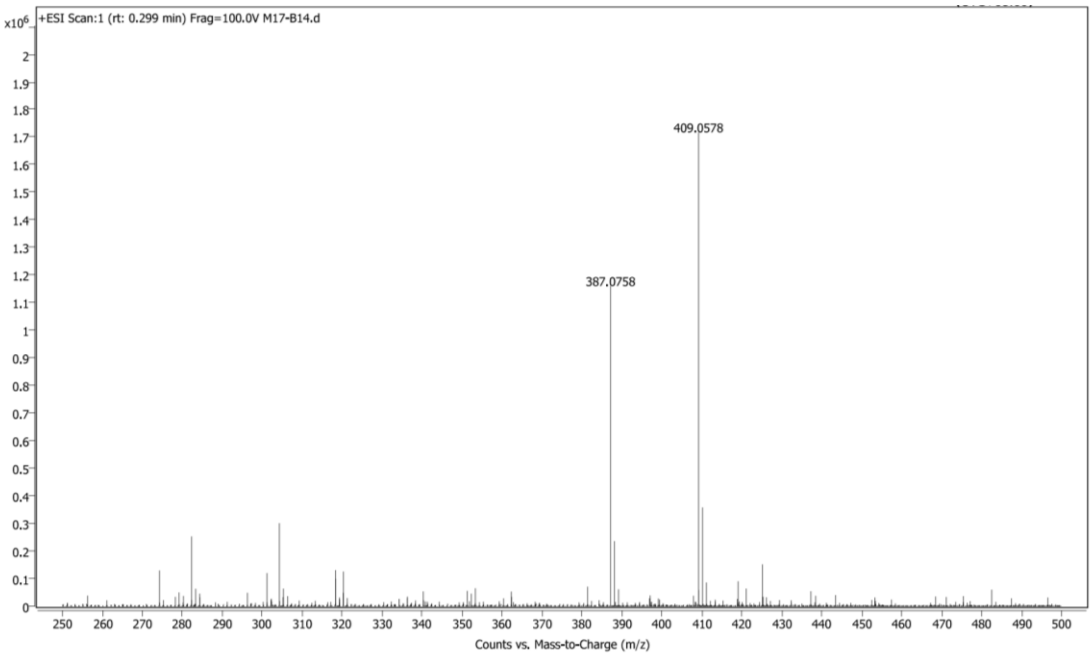

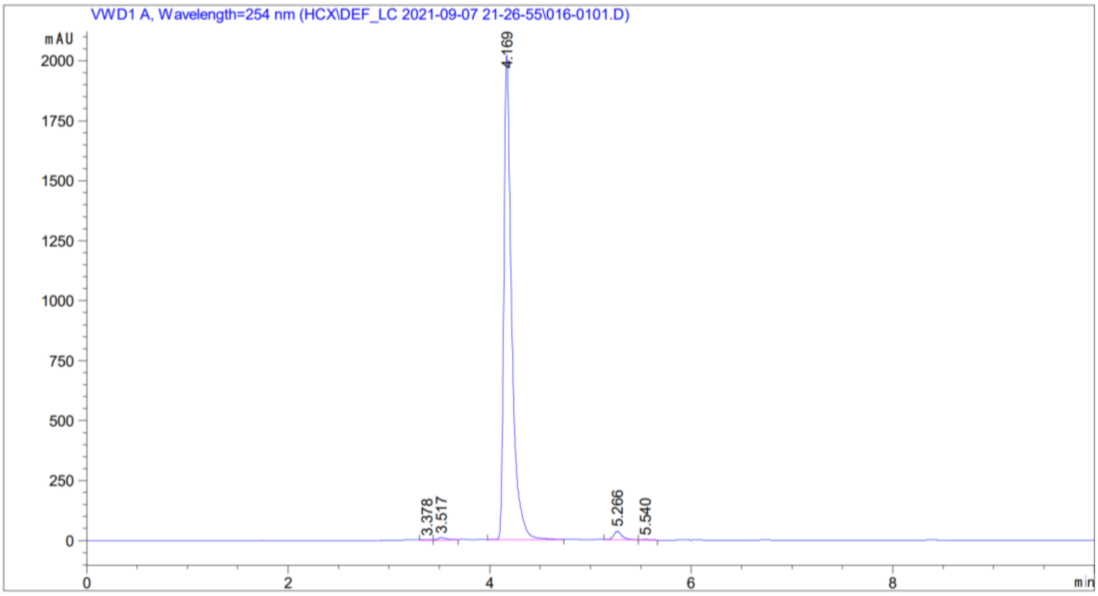

Peak Table

Peak	Ret. Time	Height	Area	Area%
1	3.378	2.1477	13.4904	0.1201
2	3.517	9.6381	64.6875	0.5760
3	4.169	2019.9238	10936.2	97.3839
4	5.266	35.4299	204.5405	1.8214
5	5.540	2.4296	11.07174	0.0986
Total		2069.5692	11230.0	100.0000

Compound M17-B15

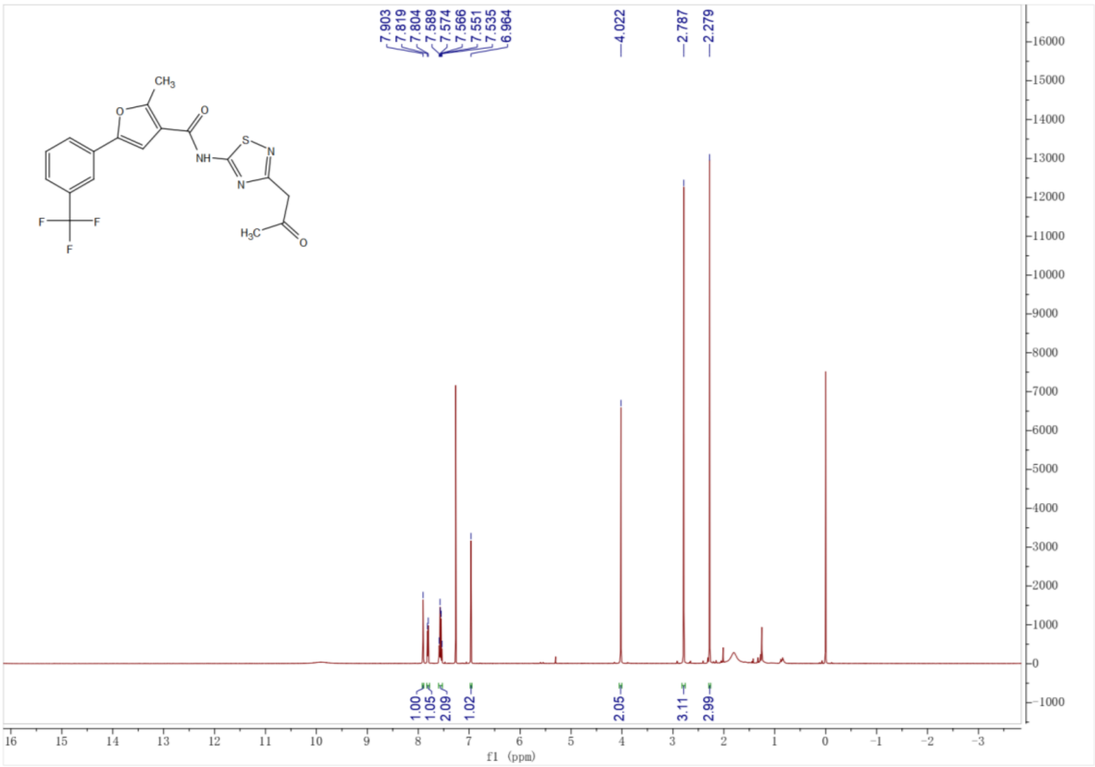

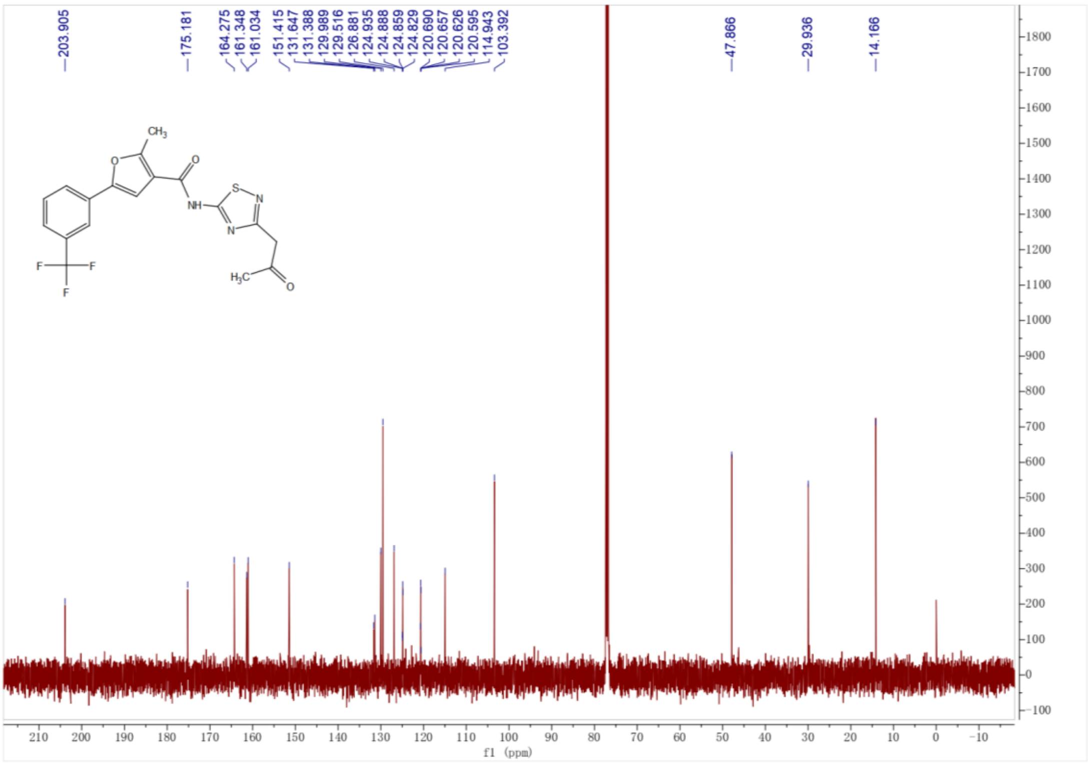

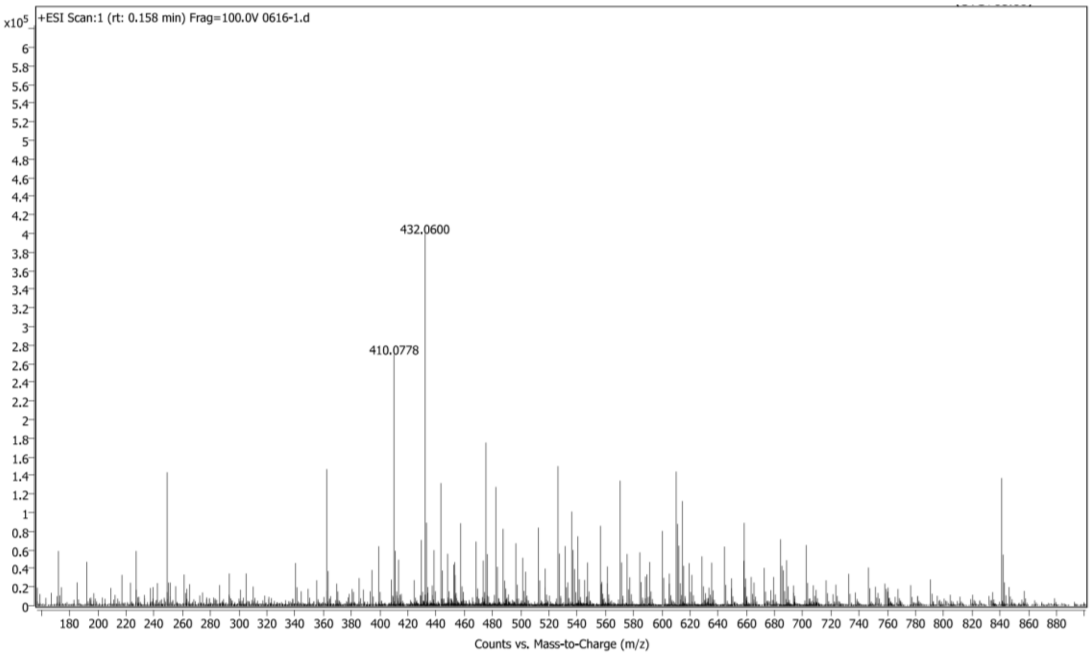

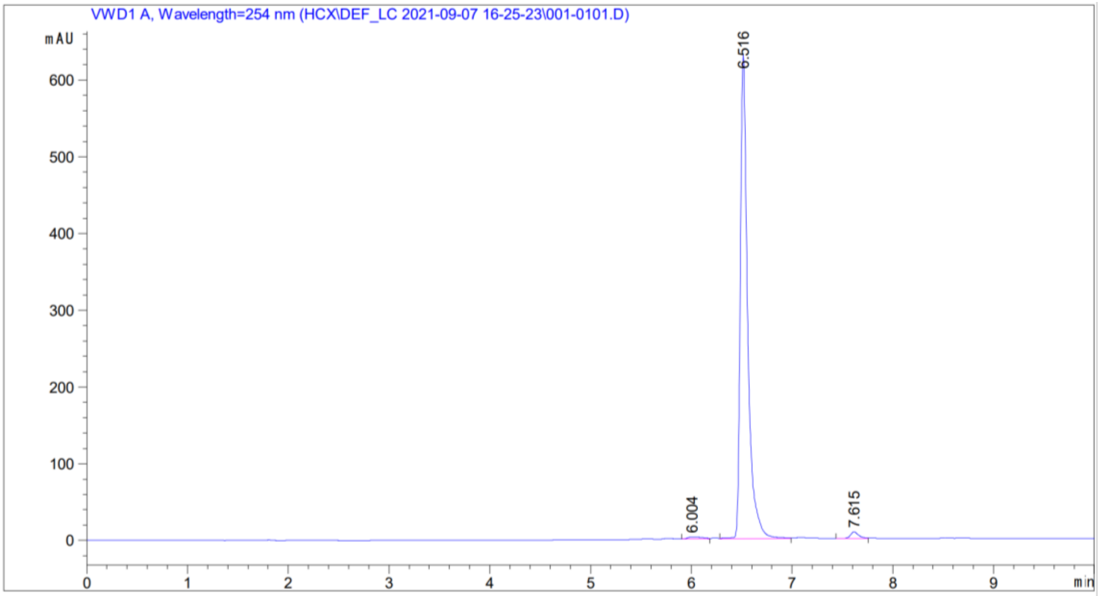

Peak Table

Peak	Ret. Time	Height	Area	Area%
1	6.004	2.4180	24.4083	0.7427
2	6.516	630.4296	3213.7109	97.7898
3	7.615	8.4458	48.2268	1.4675
Total		641.2934	3286.3460	100.0000

Compound M17-B16

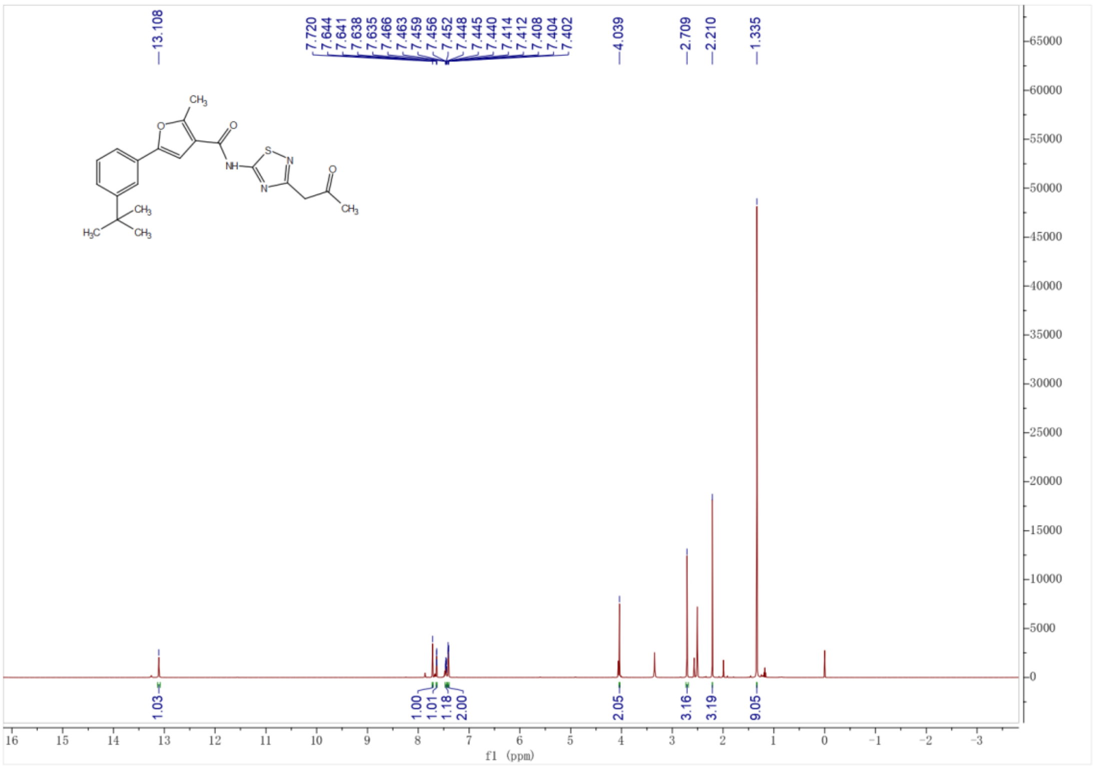

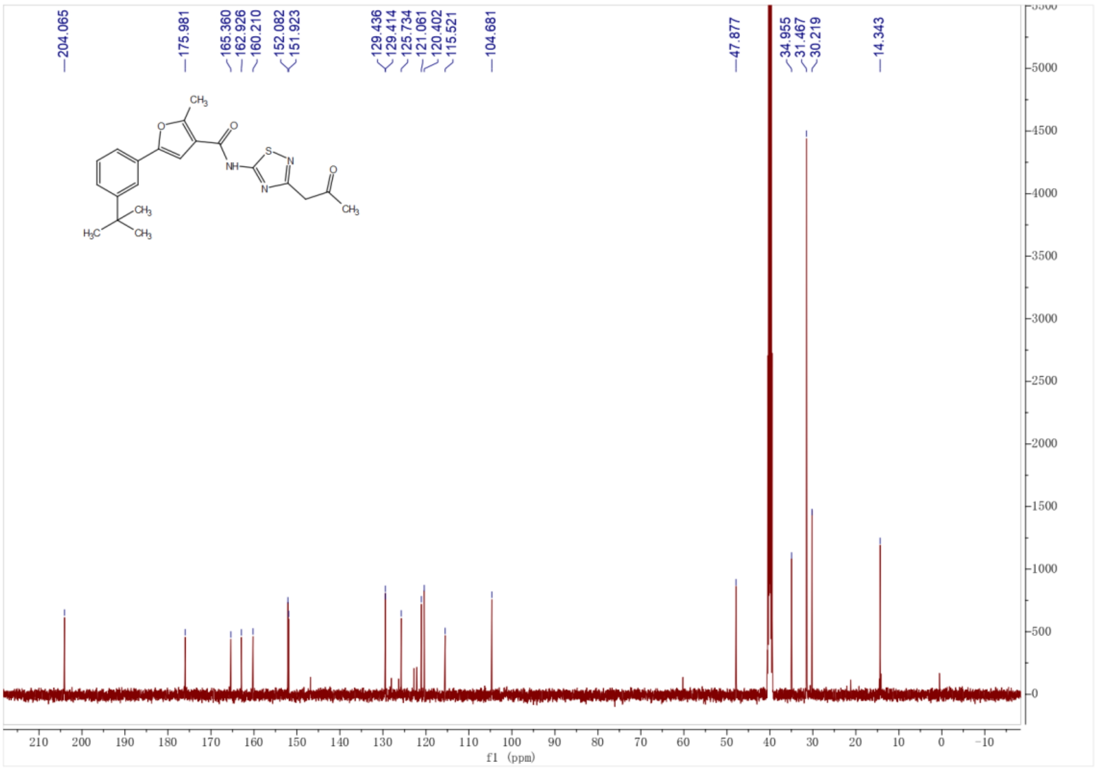

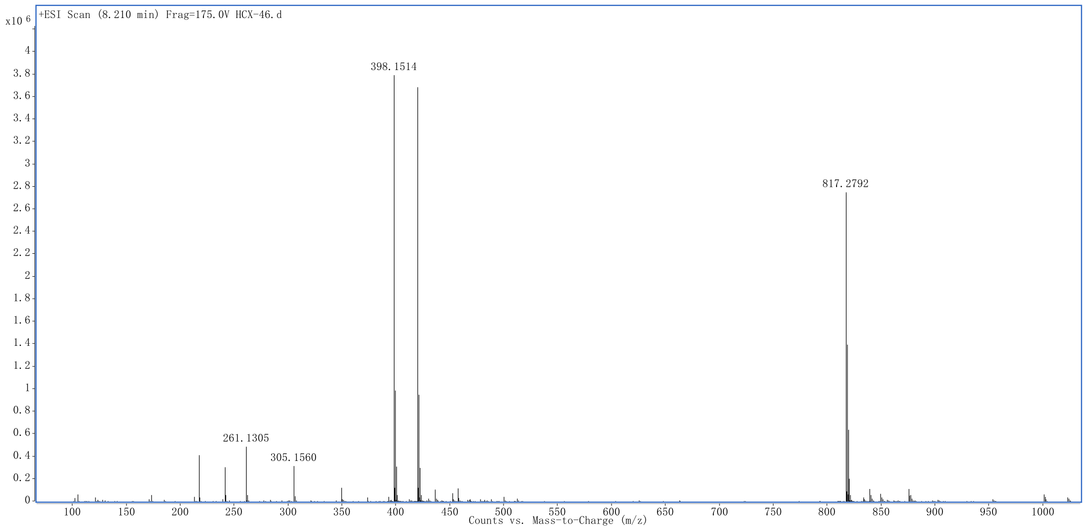

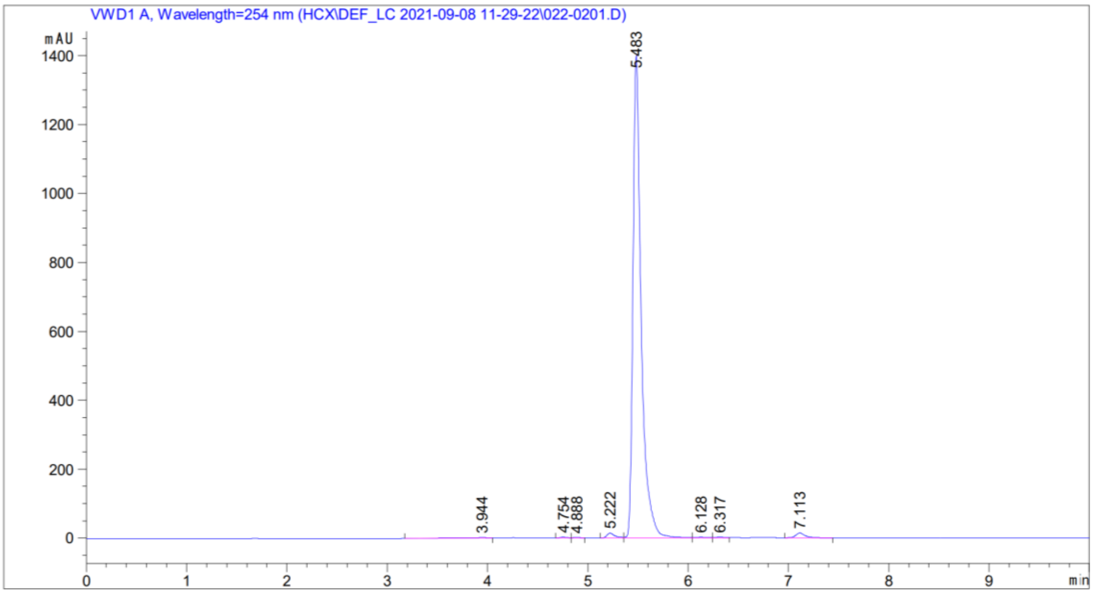

Peak Table

Peak	Ret. Time	Height	Area	Area%
1	3.944	1.7597	36.8942	0.4483
2	4.754	2.5802	11.8539	0.1440
3	4.888	1.8215	7.9216	0.0963
4	5.222	14.1903	77.9012	0.9466
5	5.483	1400.6470	7967.6201	96.8139
6	6.128	1.8838	16.2938	0.1980
7	6.317	2.1450	13.9449	0.1694
8	7.113	14.1409	97.40048	1.1836
Total		1439.1684	8229.8346	100.0000

Compound M17-B17

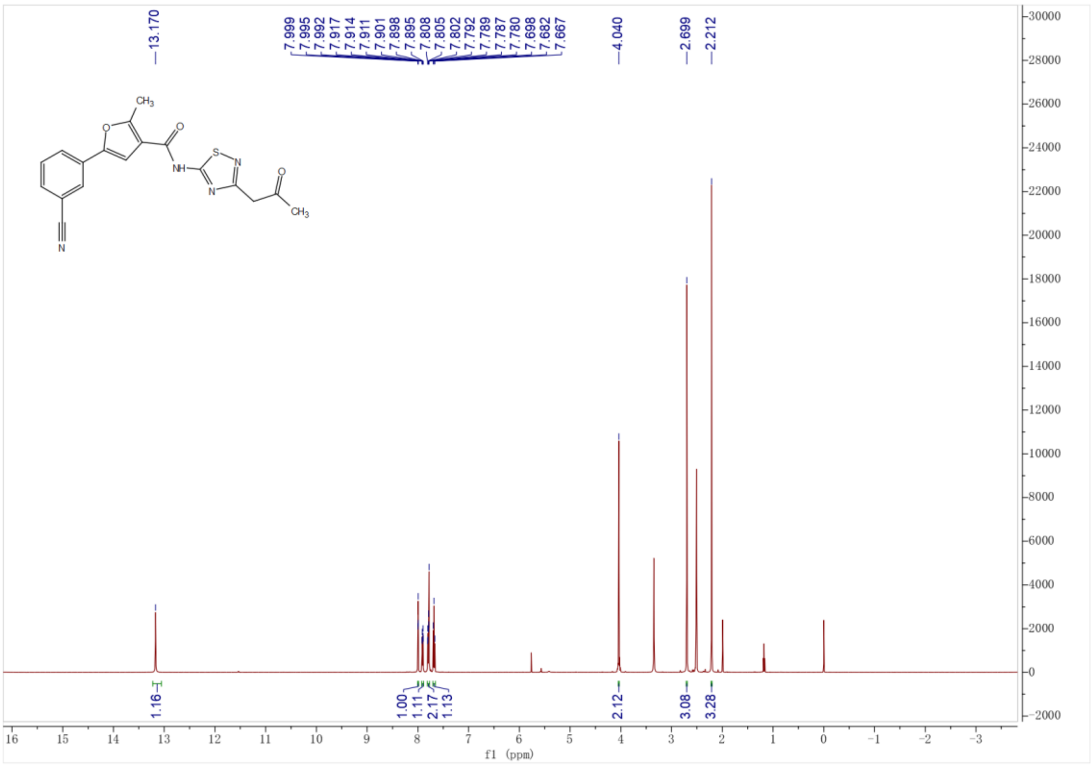

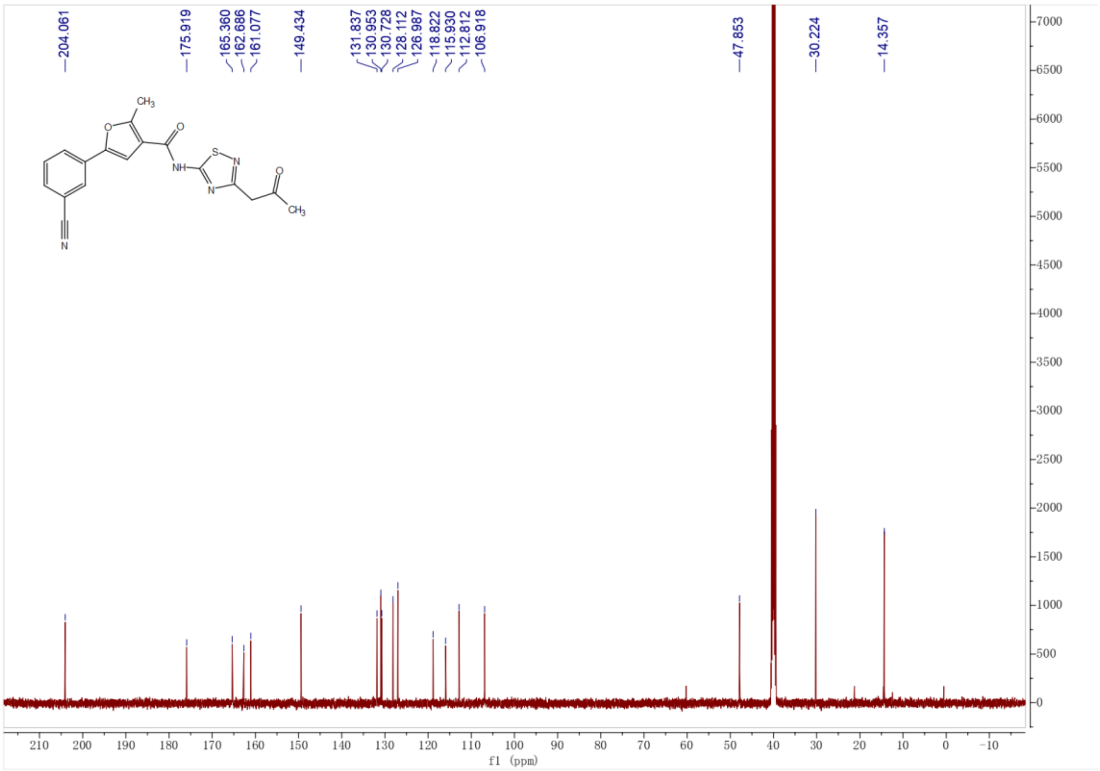

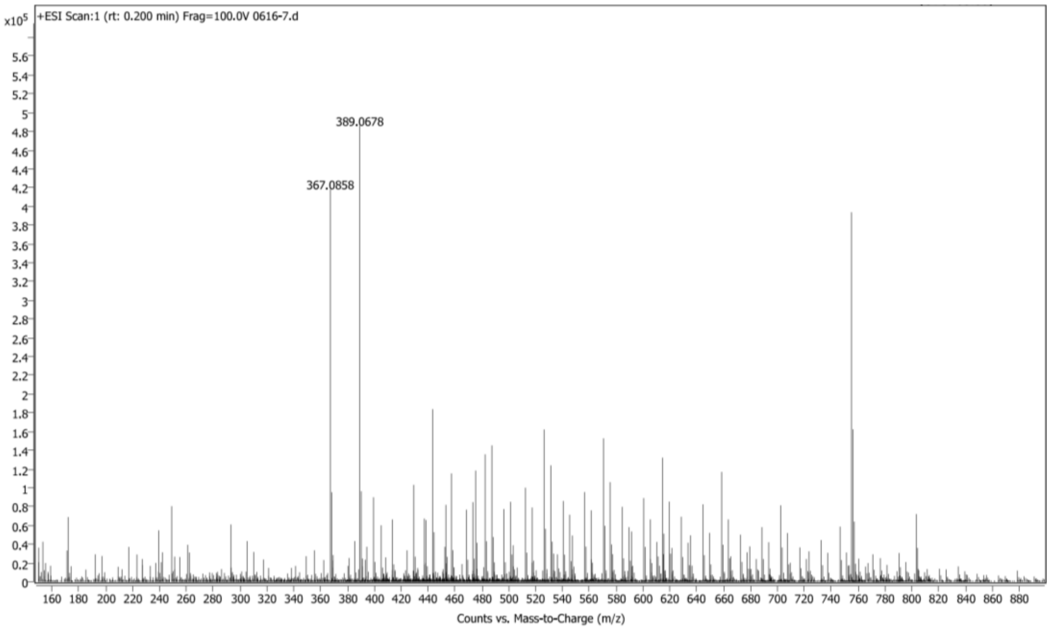

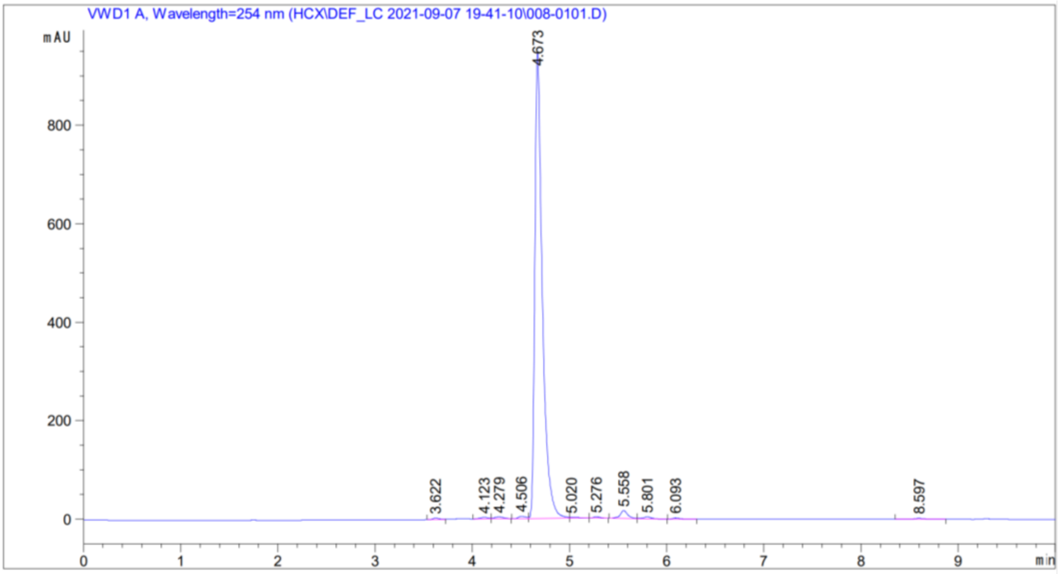

Peak Table

Peak	Ret. Time	Height	Area	Area%
1	3.622	2.8365	14.1750	0.2659
2	4.123	3.1515	18.3325	0.3439
3	4.279	3.6364	24.8210	0.4656
4	4.506	4.8044	24.4295	0.4582
5	4.673	944.9098	5083.0522	95.3439
6	5.020	1.9409	11.5127	0.2159
7	5.276	2.6369	12.6474	0.2372
8	5.558	16.1999	90.1462	1.6909
9	5.801	3.8602	24.1985	0.4539
10	6.093	2.3543	13.2656	0.2488
11	8.597	1.8310	14.7004	0.2757
Total		988.1617	5331.2810	100.0000

Compound M17-B18

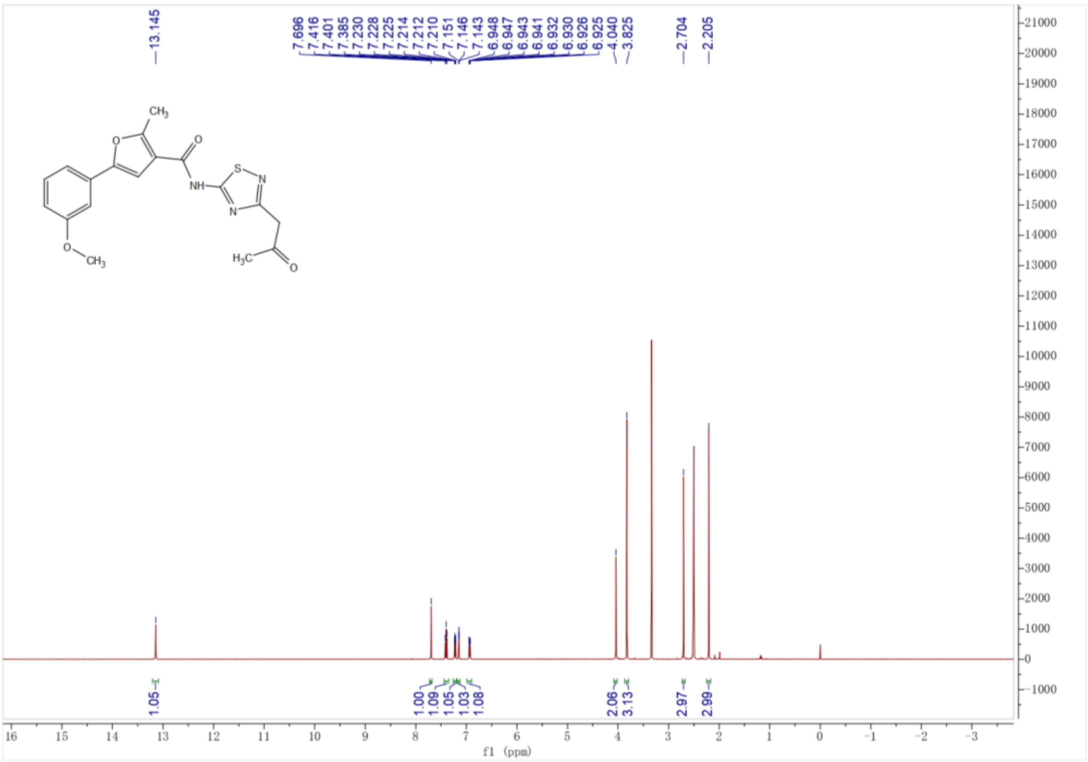

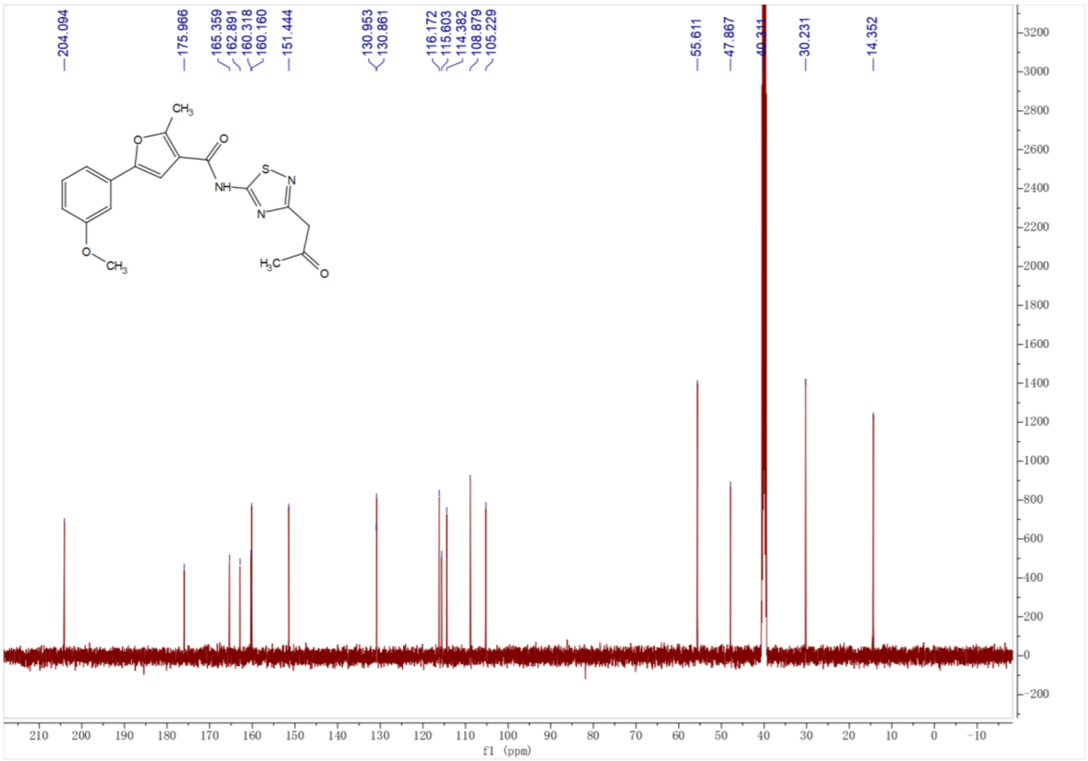

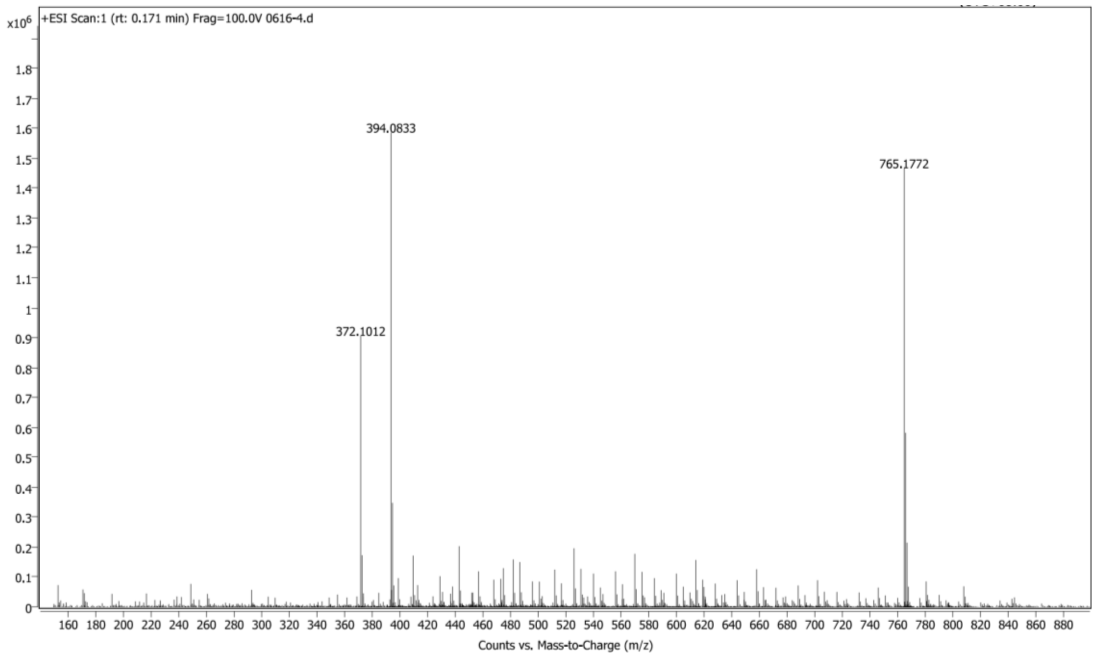

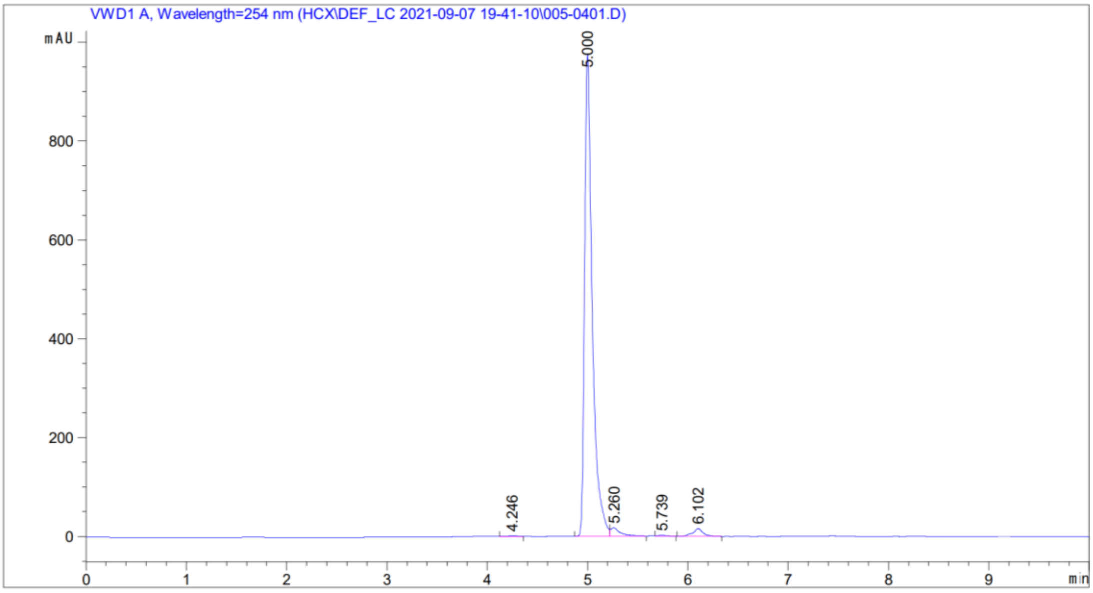

Peak Table

Peak	Ret. Time	Height	Area	Area%
1	4.246	2.3797	19.7762	0.3628
2	5.000	974.3719	5199.1948	95.3802
3	5.260	17.0892	111.8475	2.0519
4	5.739	1.7852	9.4421	0.1732
5	6.102	15.5872	110.7629	2.0320
Total		1011.2121	5451.0235	100.0000

Compound M17-B19

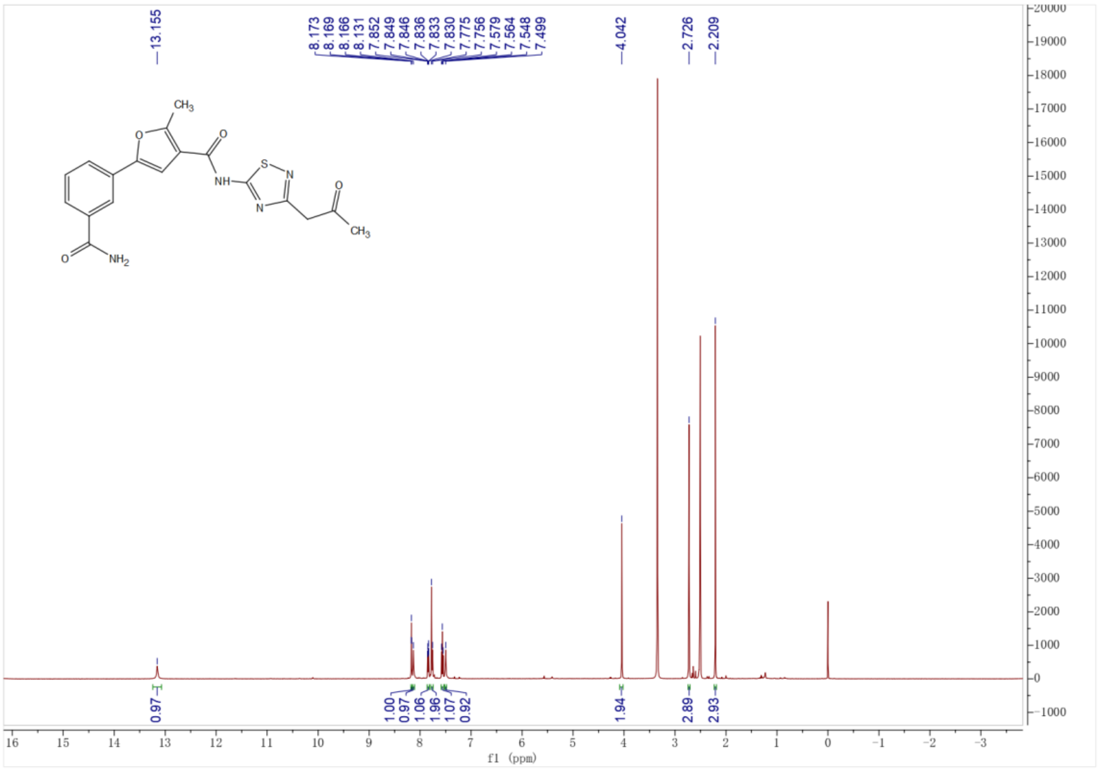

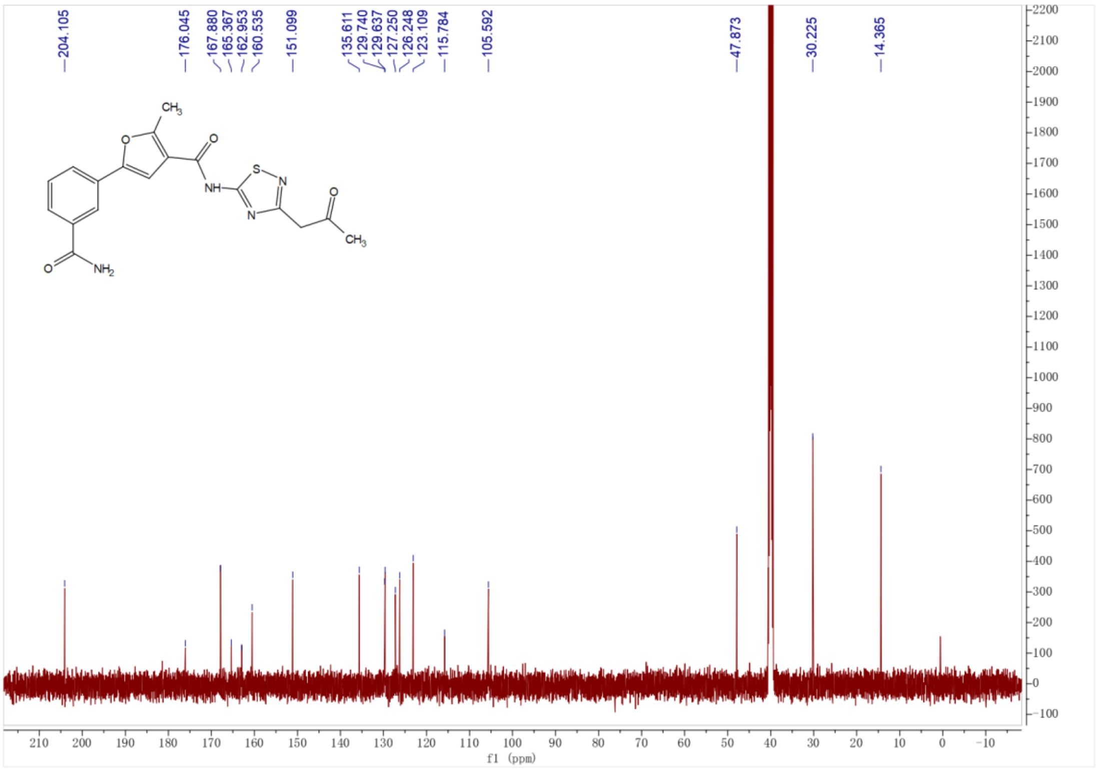

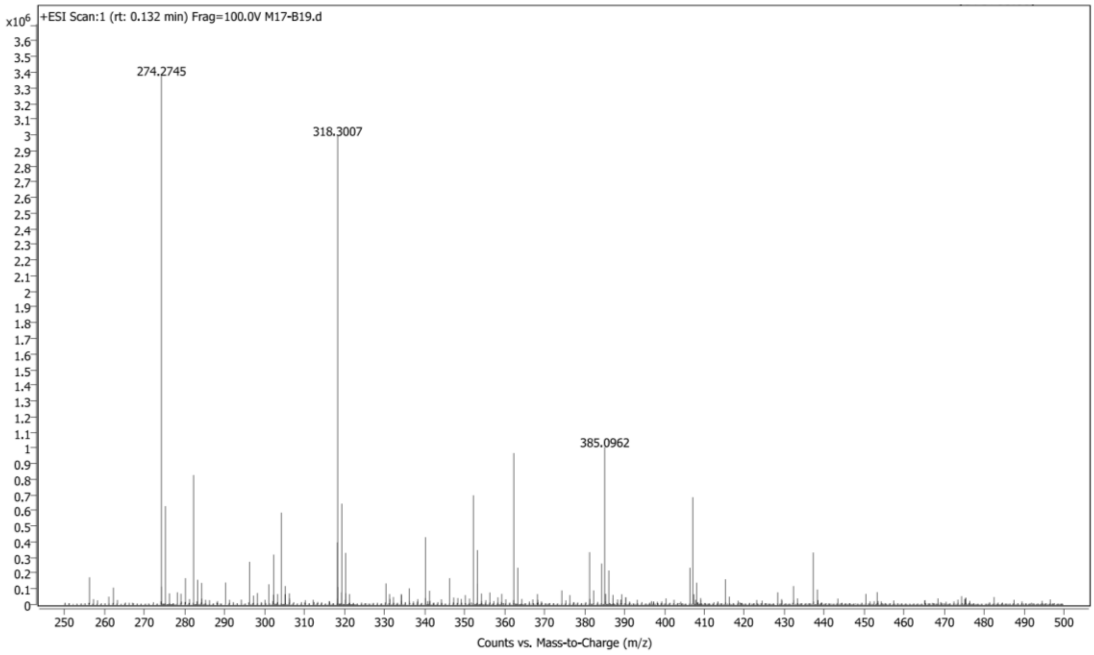

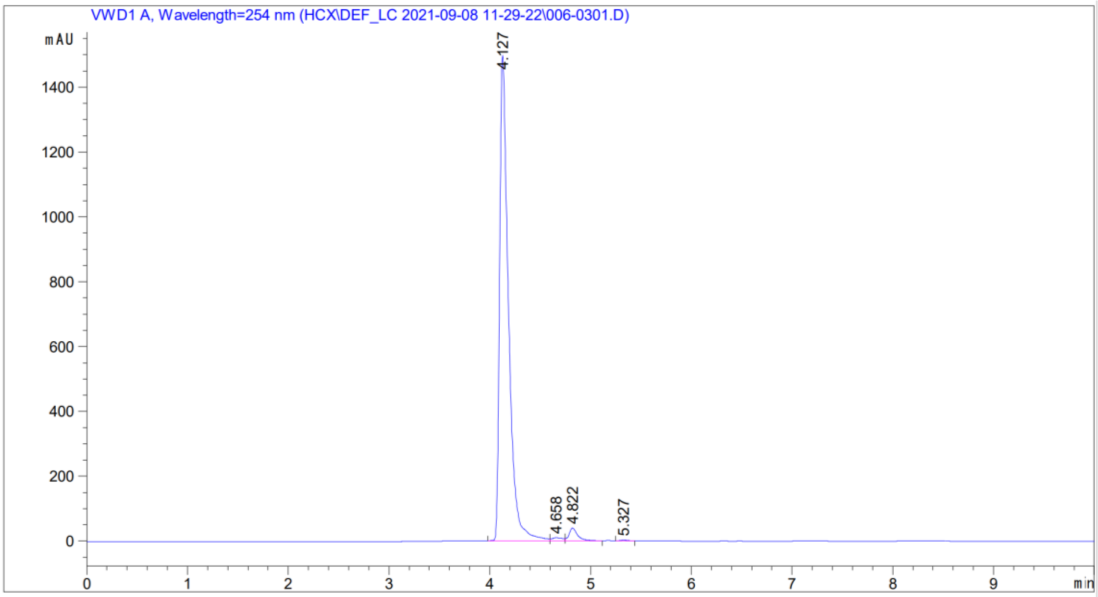

Peak Table

Peak	Ret. Time	Height	Area	Area%
1	4.127	1495.1531	9146.0137	96.6627
2	4.658	9.4623	70.1409	0.7413
3	4.822	39.7155	231.8168	2.4500
4	5.327	2.7297	13.8130	0.1460
Total		1547.0606	9461.7844	100.0000

Compound M17-B20

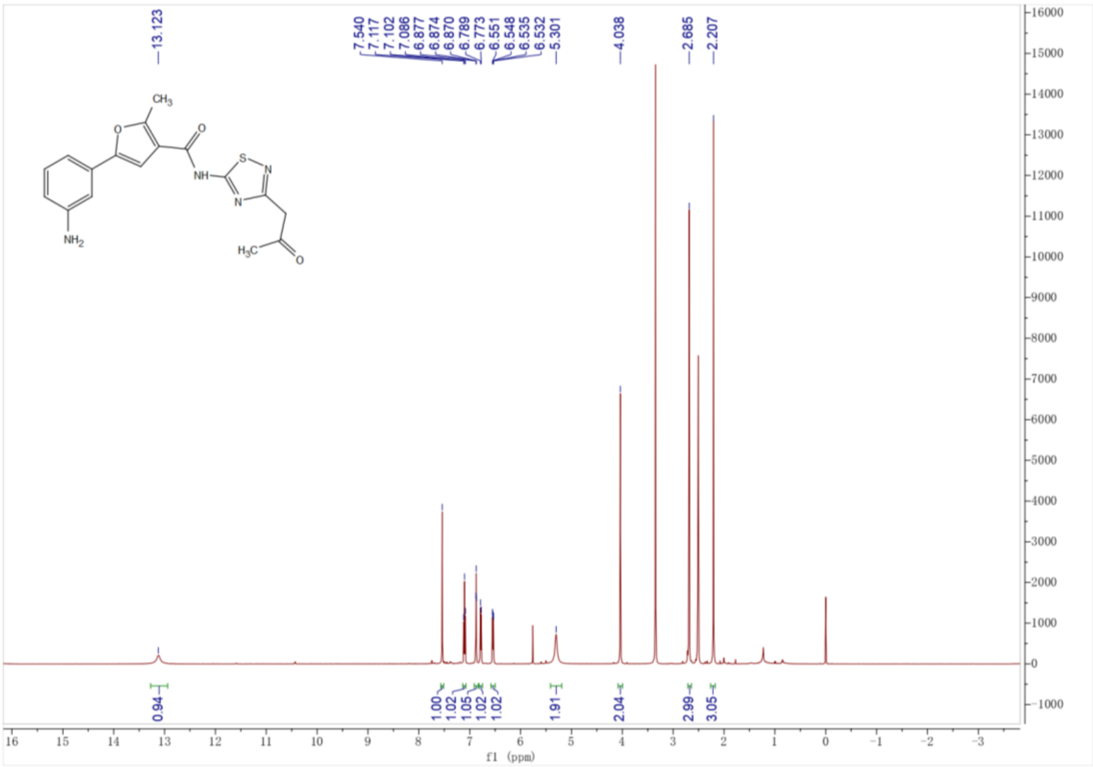

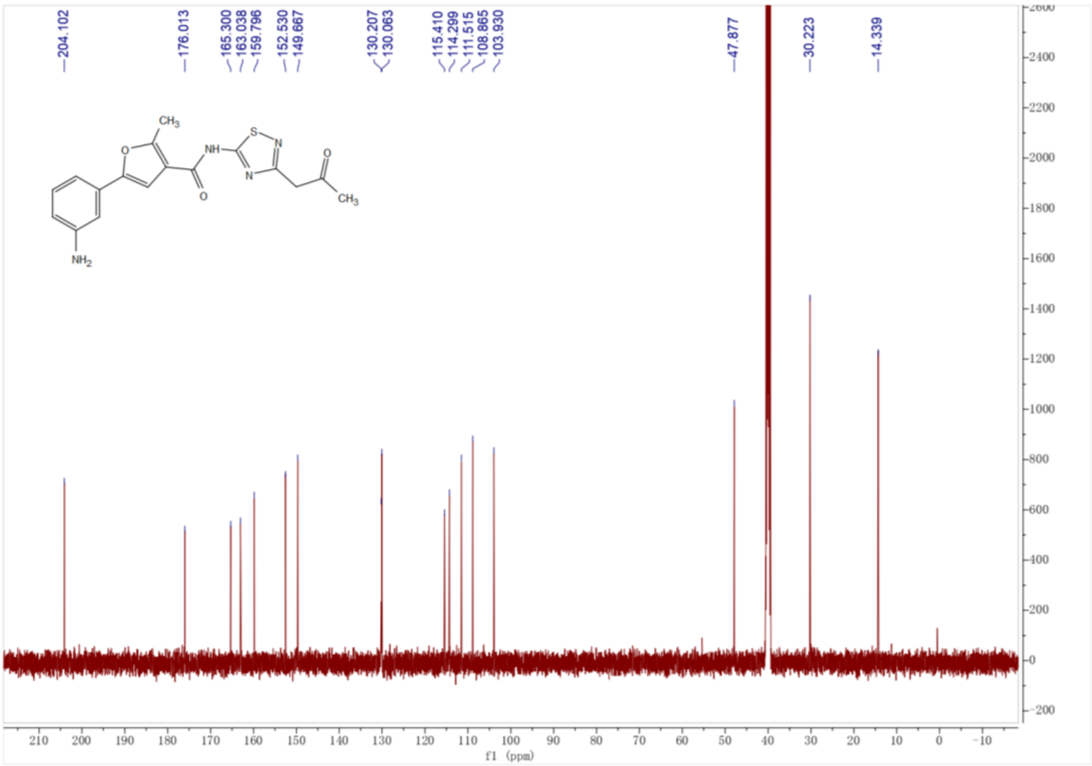

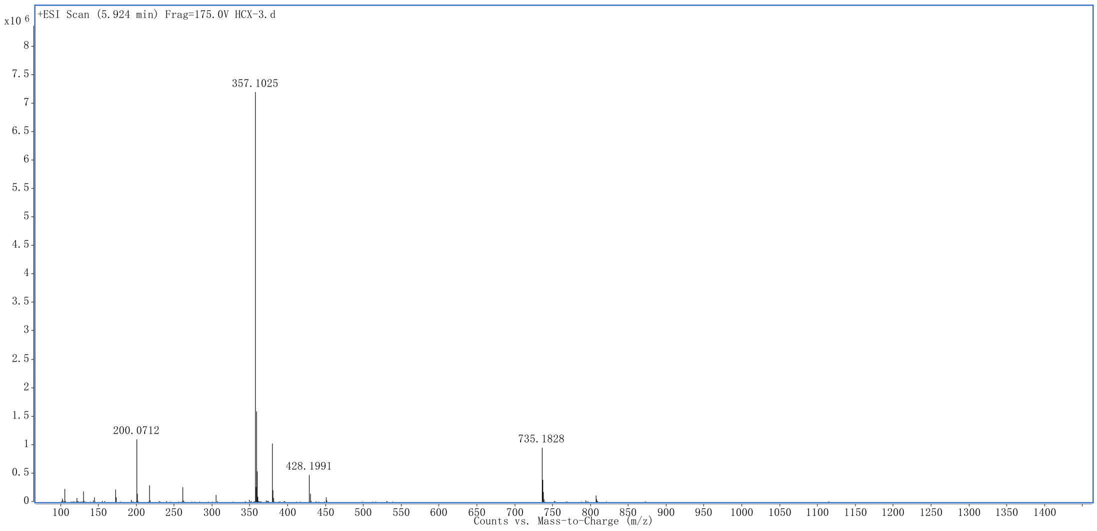

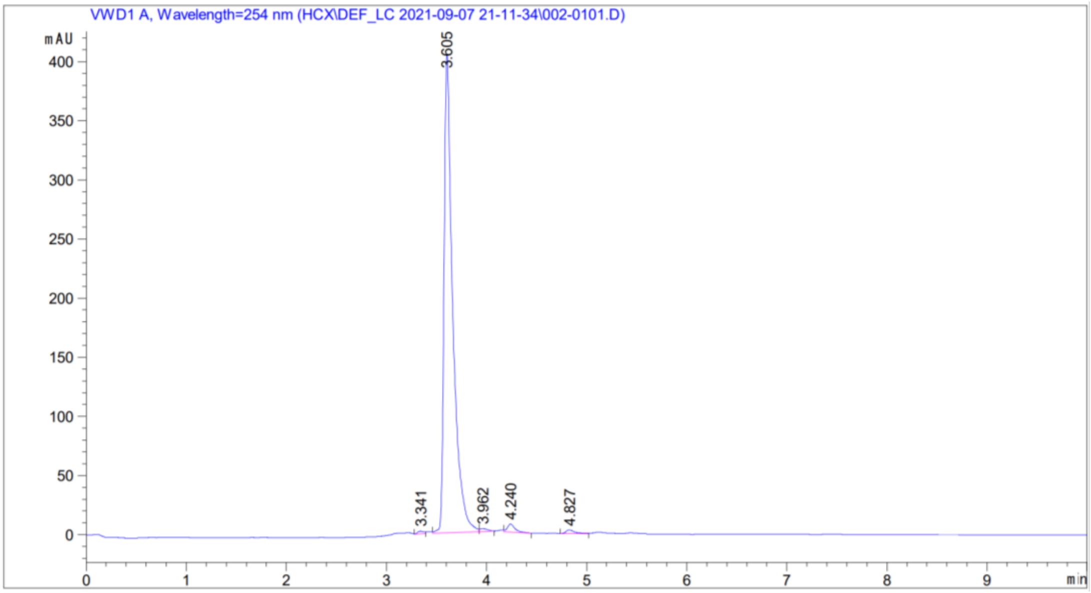

Peak Table

Peak	Ret. Time	Height	Area	Area%
1	3.341	2.2148	9.4466	0.3741
2	3.605	404.1265	2447.0376	96.9156
3	3.962	2.4888	13.6161	0.5393
4	4.240	6.8270	36.0158	1.4264
5	4.827	3.0900	18.9010	0.7446
Total		418.7471	2524.9171	100.0000

Compound M17-B21

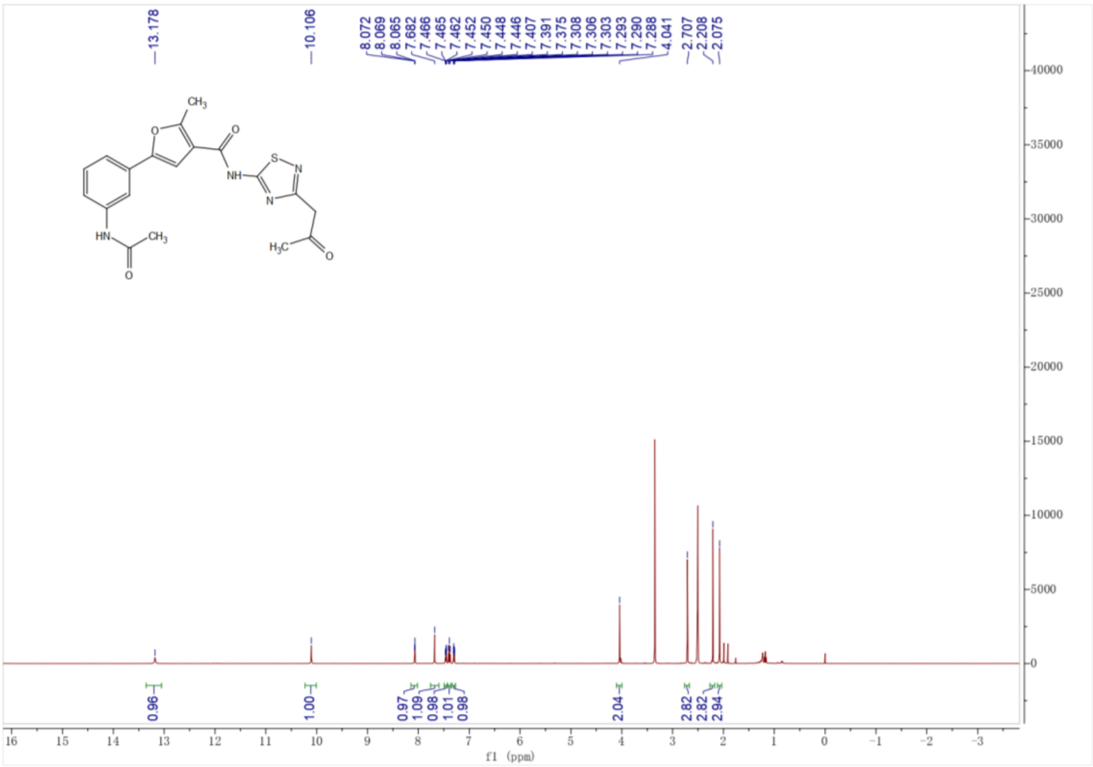

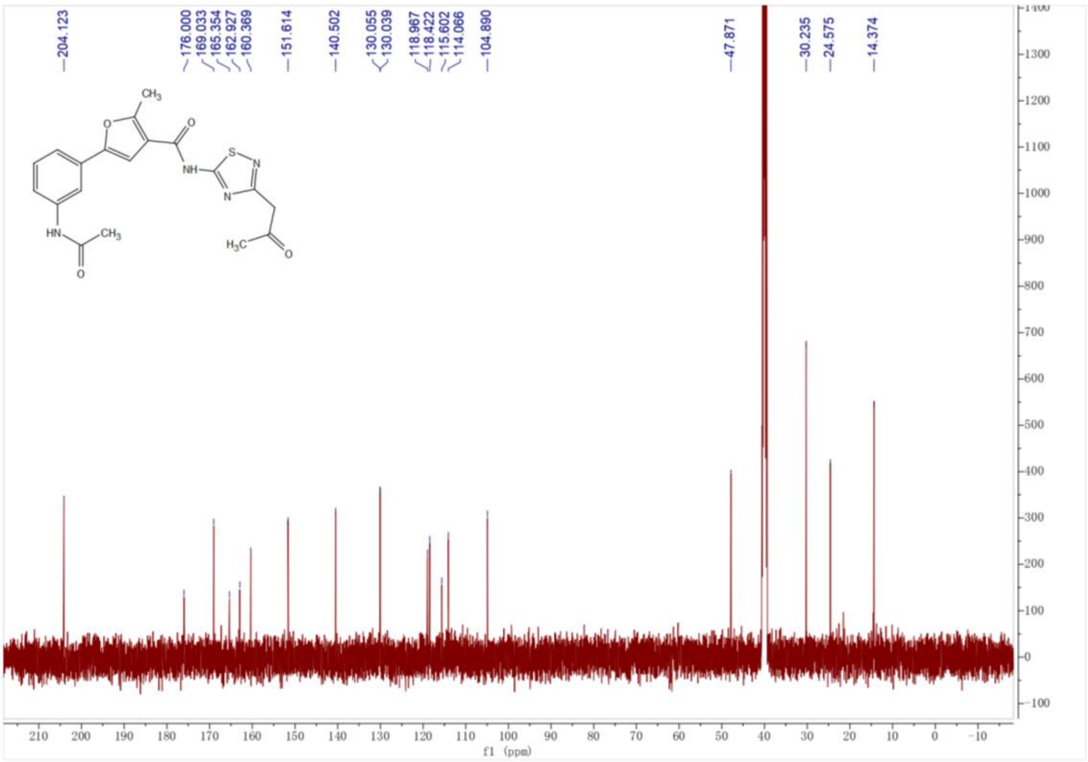

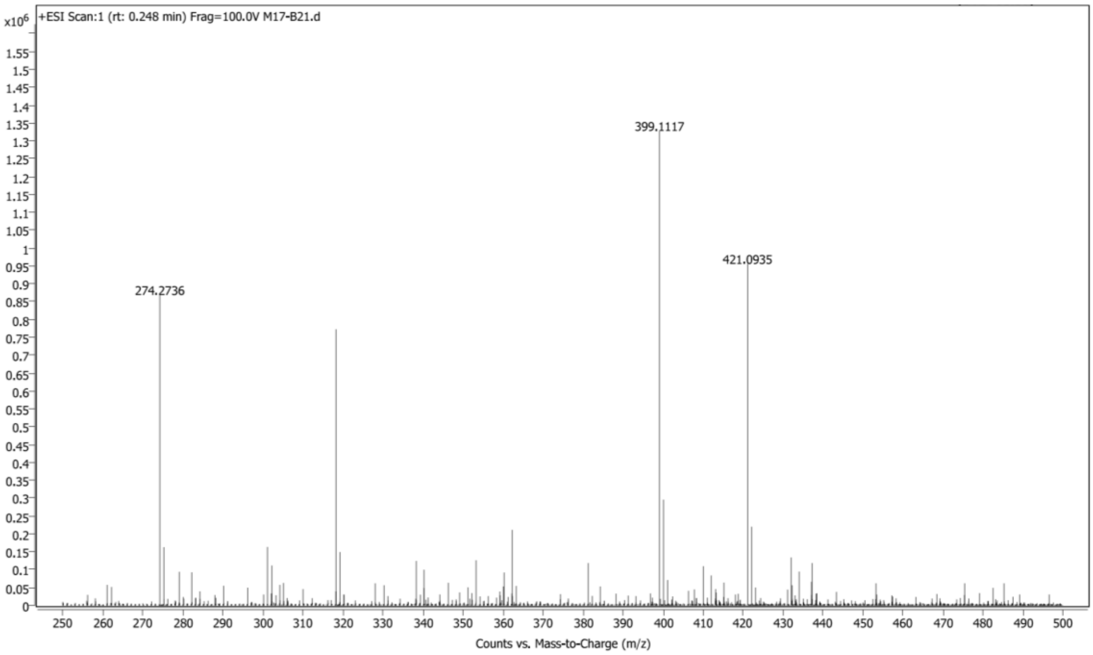

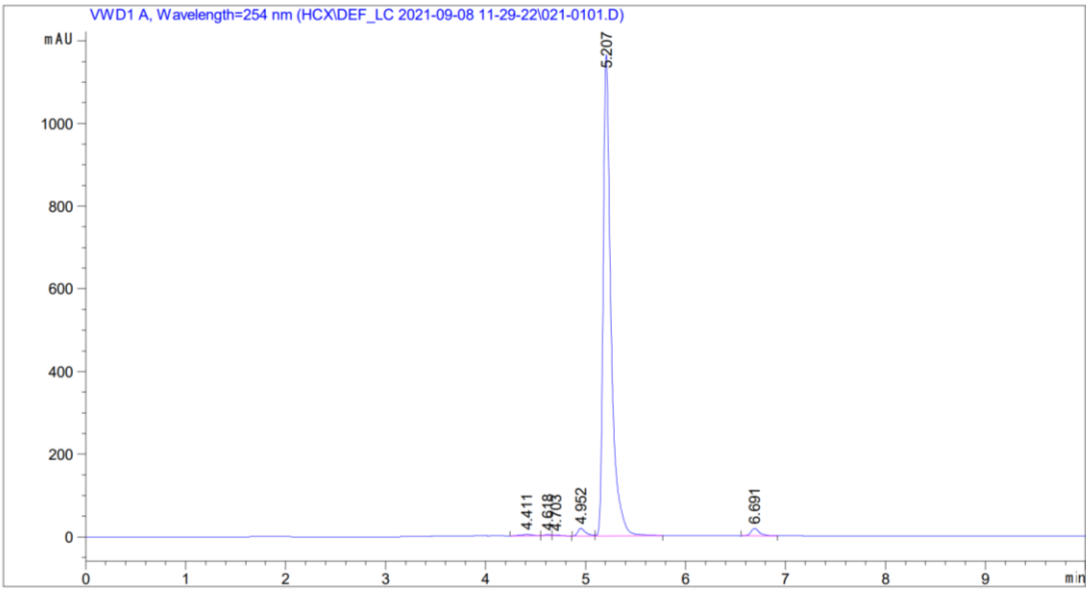

Peak Table

Peak	Ret. Time	Height	Area	Area%
1	4.411	3.3690	24.7860	0.3766
2	4.618	2.7854	11.0666	0.1681
3	4.703	2.0154	10.1204	0.1538
4	4.952	18.5294	97.5546	1.4821
5	5.207	1162.3748	6330.9058	96.1845
6	6.691	17.9834	107.6095	1.6349
Total		1207.0573	6582.0428	100.0000

Compound M17-B22

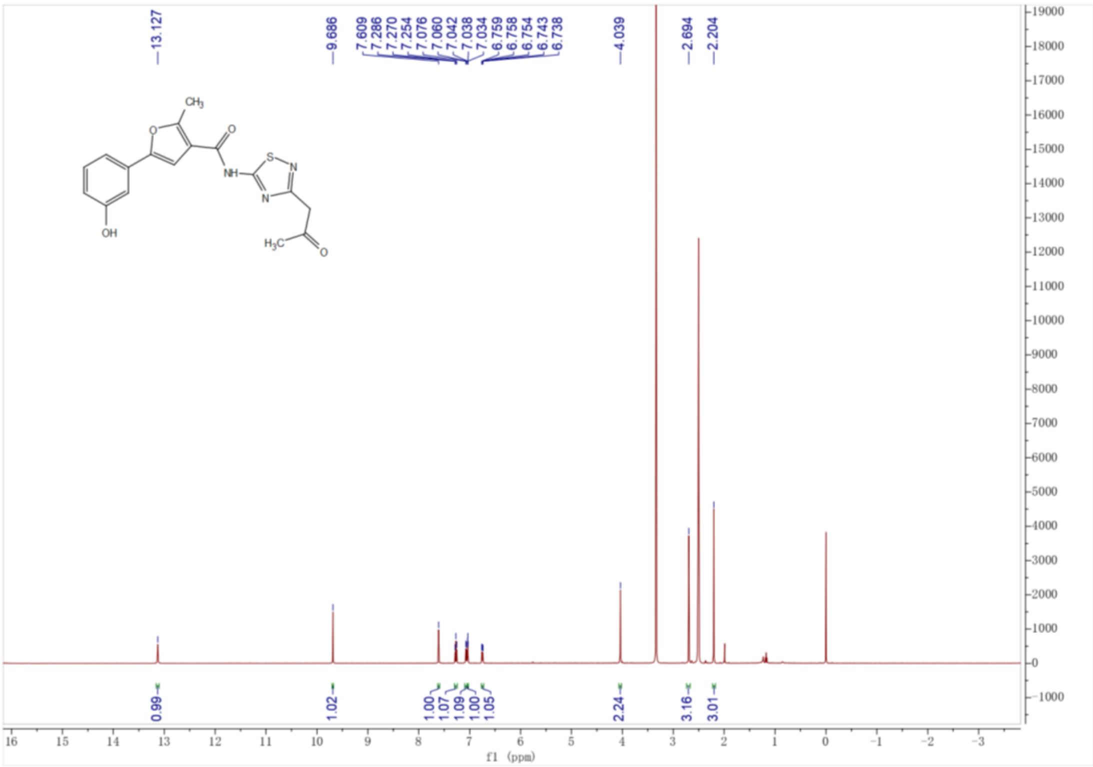

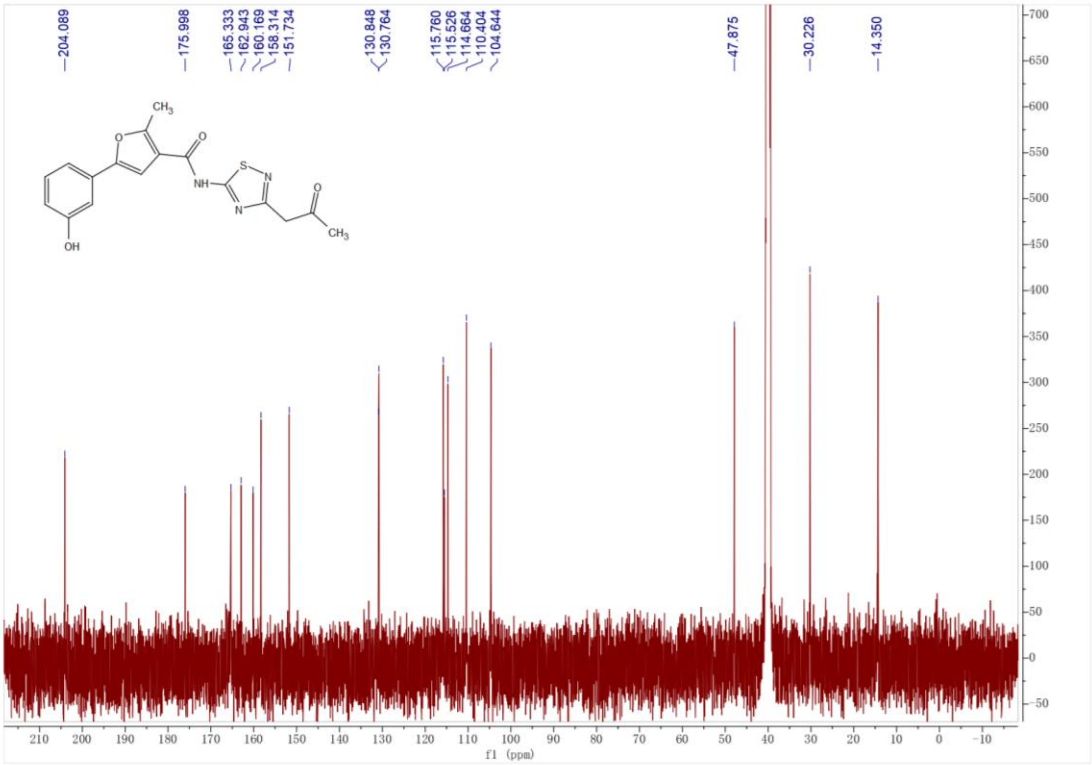

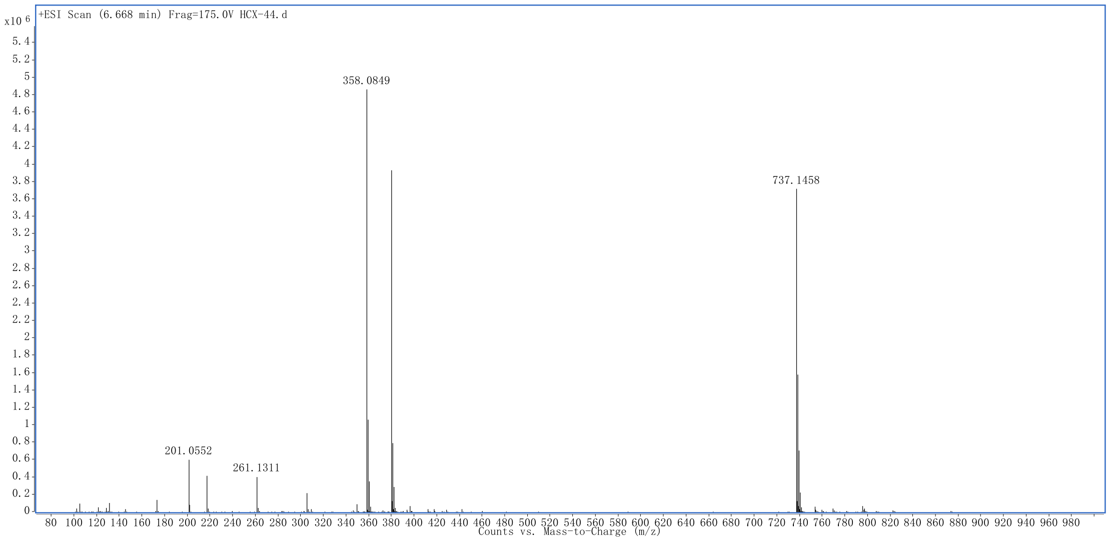

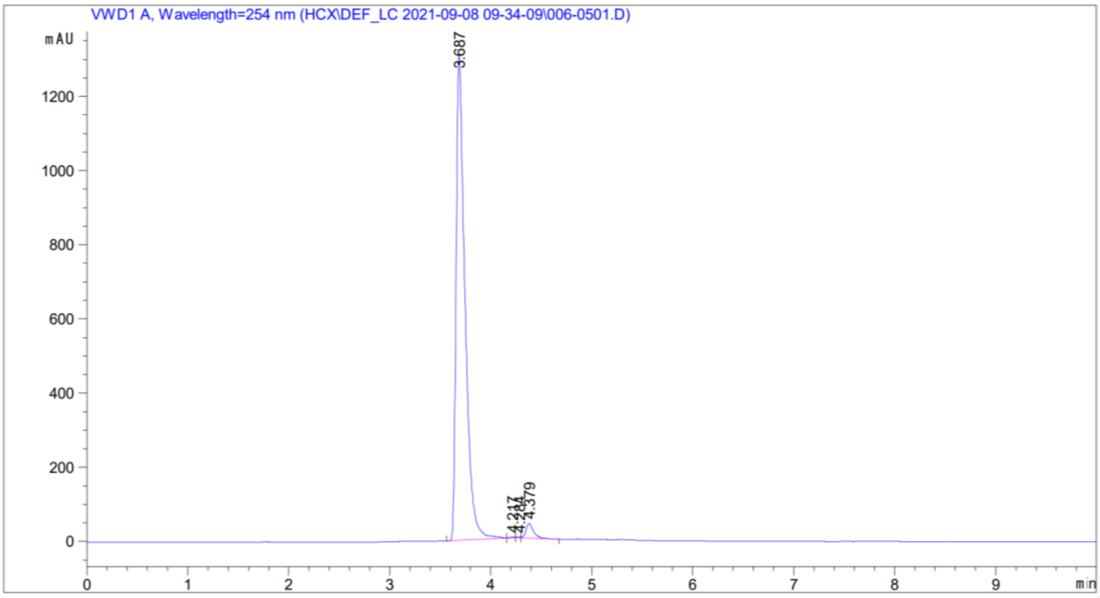

Peak Table

Peak	Ret. Time	Height	Area	Area%
1	3.687	1307.2364	8009.9146	97.0814
2	4.217	2.8467	9.4928	0.1151
3	4.284	3.5966	11.0749	0.1342
4	4.379	40.3084	220.2353	2.6693
Total		1353.9882	8250.7175	100.0000

Compound M17-B23

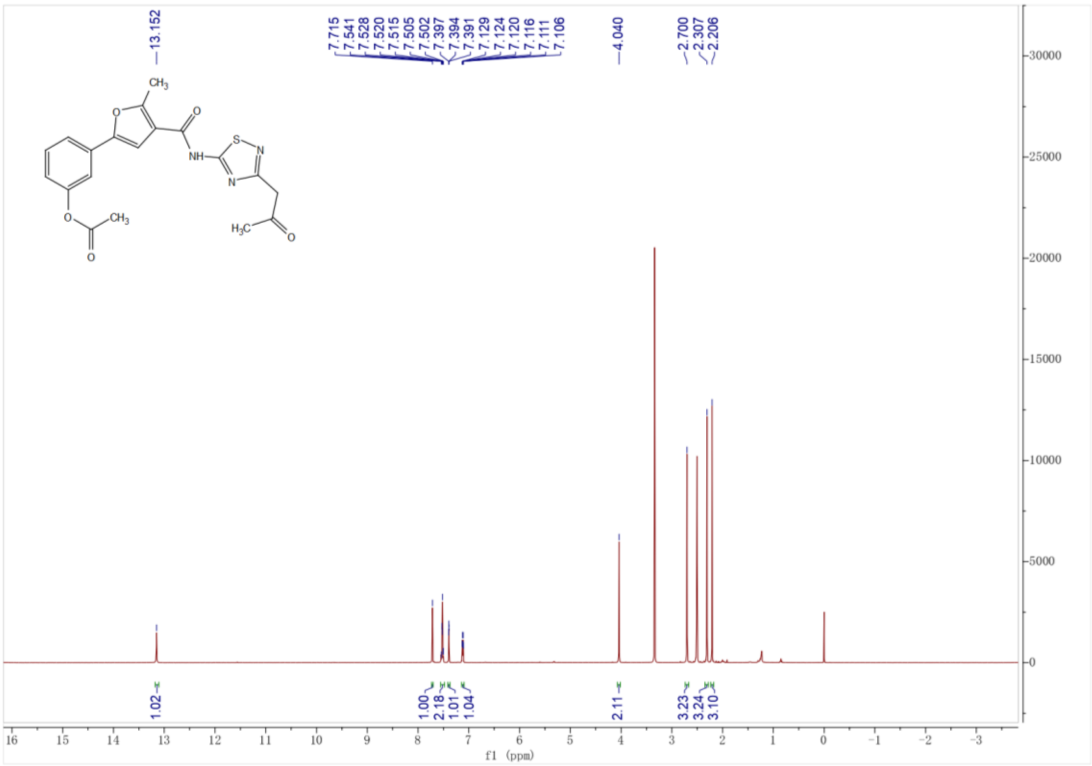

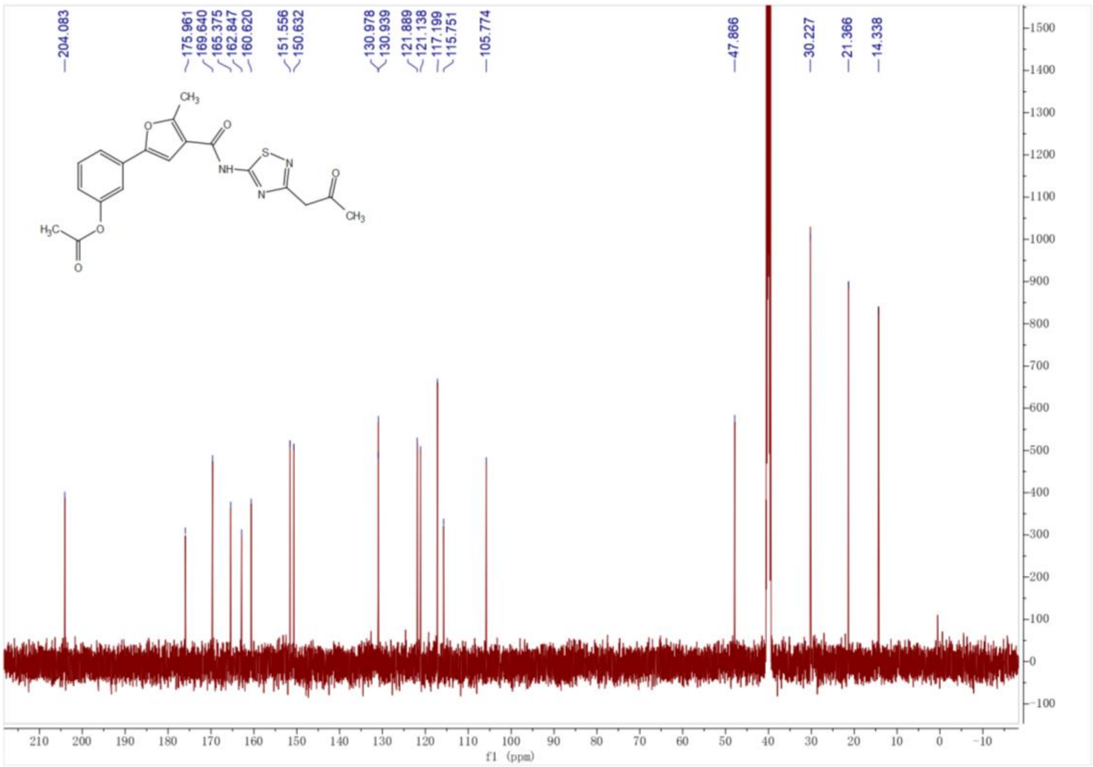

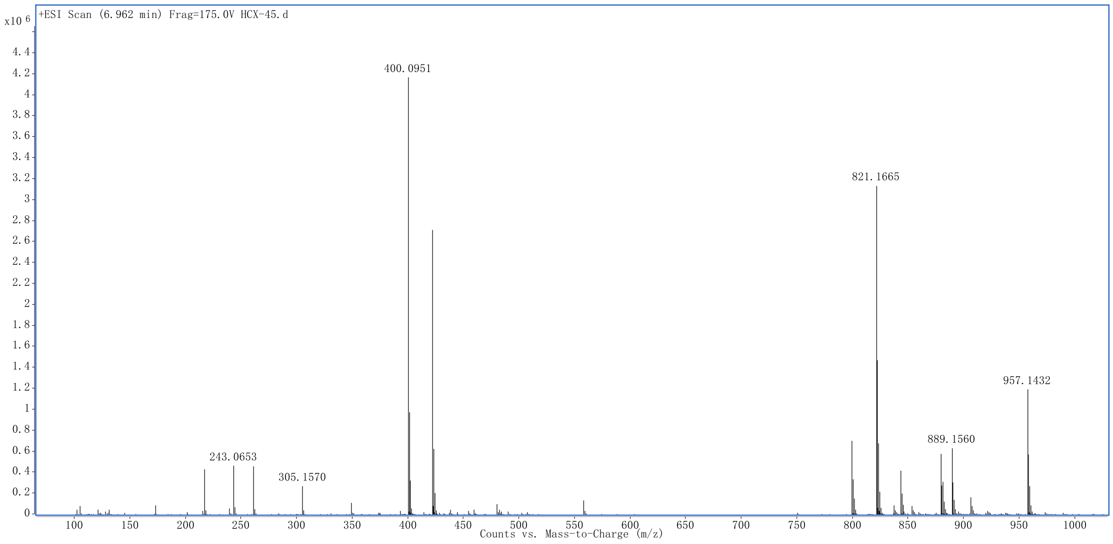

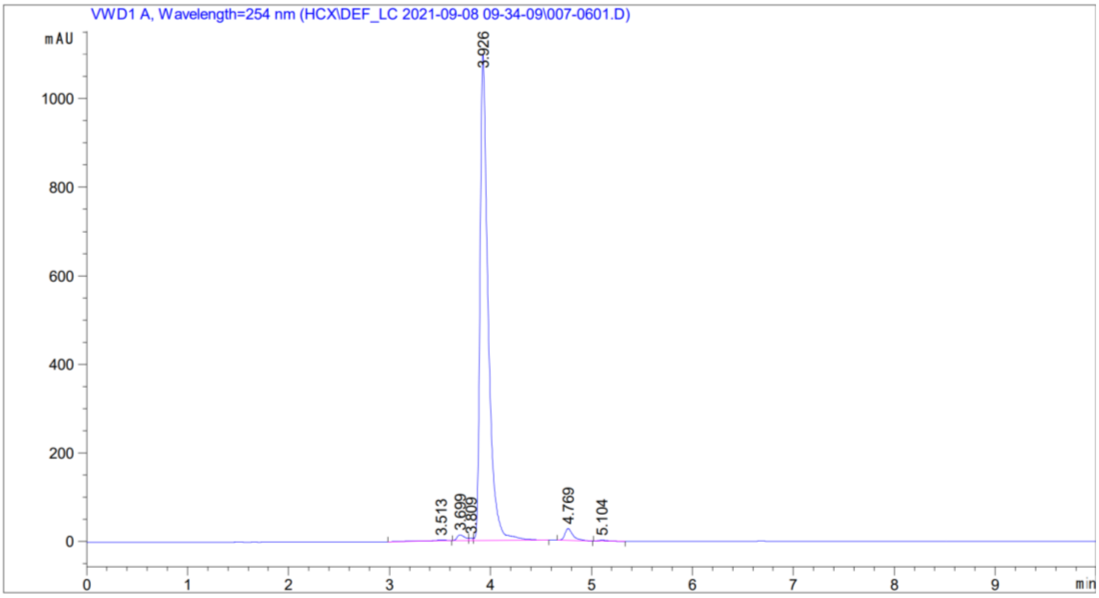

Peak Table

Peak	Ret. Time	Height	Area	Area%
1	3.513	1.7389	21.6274	0.3385
2	3.699	12.8405	67.8867	1.0627
3	3.809	4.6761	11.8918	0.1861
4	3.926	1095.3127	6124.3144	95.8667
5	4.769	26.9448	150.8300	2.3610
6	5.104	2.1615	11.8115	0.1849
Total		1143.6746	6388.3618	100.0000

Supplementary References

(1) Nadal, M.; Prekovic, S.; Gallastegui, N.; Helsen, C.; Abella, M.; Zielinska, K.; Gay, M.; Vilaseca, M.; Taules, M.; Houtsmuller, A. B.; et al. Structure of the homodimeric androgen receptor ligand-binding domain. *Nat. Commun.* **2017**, *8*, 14388. DOI: 10.1038/ncomms14388.

(2) Lee, T. S.; Cerutti, D. S.; Mermelstein, D.; Lin, C.; LeGrand, S.; Giese, T. J.; Roitberg, A.; Case, D. A.; Walker, R. C.; York, D. M. GPU-Accelerated Molecular Dynamics and Free Energy Methods in Amber18: Performance Enhancements and New Features. *J. Chem. Inf. Model.* **2018**, *58* (10), 2043-2050. DOI: 10.1021/acs.jcim.8b00462.

(3) Frisch, M.; Trucks, G.; Schlegel, H.; Scuseria, G.; Robb, M.; Cheeseman, J.; Scalmani, G.; Barone, V.; Petersson, G.; Nakatsuji, H. Gaussian 16. Gaussian, Inc. Wallingford, CT: 2016.

(4) Wang, J.; Wang, W.; Kollman, P. A.; Case, D. A. Antechamber: an accessory software package for molecular mechanical calculations. *J. Am. Chem. Soc.* **2001**, *222*, U403.

(5) Wang, J.; Wolf, R. M.; Caldwell, J. W.; Kollman, P. A.; Case, D. A. Development and testing of a general amber force field. *J. comput. chem.* **2004**, *25* (9), 1157-1174. DOI: 10.1002/jcc.20035.

(6) Maier, J. A.; Martinez, C.; Kasavajhala, K.; Wickstrom, L.; Hauser, K. E.; Simmerling, C. ff14SB: improving the accuracy of protein side chain and backbone parameters from ff99SB. *J. Chem. Theory Comput.* **2015**, *11* (8), 3696-3713. DOI: 10.1021/acs.jctc.5b00255.

(7) Loncharich, R. J.; Brooks, B. R.; Pastor, R. W. Langevin dynamics of peptides: the frictional dependence of isomerization rates of N-acetylalanyl-N'-methylamide. *Biopolymers* **1992**, *32* (5), 523-535. DOI: 10.1002/bip.360320508.

(8) Berendsen, H. J. C.; Postma, J. P. M.; van Gunsteren, W. F.; DiNola, A.; Haak, J. R. Molecular dynamics with coupling to an external bath. *J. Chem. Phys.* **1984**, *81* (8), 3684-3690. DOI: 10.1063/1.448118.

(9) Sagui, C.; Darden, T. A. Molecular dynamics simulations of biomolecules: long-range electrostatic effects. *Annual review of biophysics and biomolecular structure* **1999**, *28*, 155-179.

DOI: 10.1146/annurev.biophys.28.1.155.
(10) Kräutler, V.; Van Gunsteren, W. F.; Hünenberger, P. H. A fast SHAKE algorithm to solve distance constraint equations for small molecules in molecular dynamics simulations. *J. Comput. Chem.* **2001**, *22* (5), 501-508. DOI: 10.1002/1096-987X(20010415)22:5<501::AID-JCC1021>3.0.CO;2-V.
(11) Fu, W.; Zhang, M.; Liao, J.; Tang, Q.; Lei, Y.; Gong, Z.; Shan, L.; Duan, M.; Chai, X.; Pang, J.; et al. Discovery of a Novel Androgen Receptor Antagonist Manifesting Evidence to Disrupt the Dimerization of the Ligand-Binding Domain via Attenuating the Hydrogen-Bonding Network Between the Two Monomers. *J. Med. Chem.* **2021**, *64* (23), 17221-17238. DOI: 10.1021/acs.jmedchem.1c01287.
(12) Zhou, W.; Duan, M.; Fu, W.; Pang, J.; Tang, Q.; Sun, H.; Xu, L.; Chang, S.; Li, D.; Hou, T. Discovery of novel androgen receptor ligands by structure-based virtual screening and bioassays. *Genom. Proteom. Bioinf.* **2018**, *16* (6), 416-427. DOI: 10.1016/j.gpb.2018.03.007.
(13) Chen, Z. L.; Meng, J. M.; Cao, Y.; Yin, J. L.; Fang, R. Q.; Fan, S. B.; Liu, C.; Zeng, W. F.; Ding, Y. H.; Tan, D.; et al. A high-speed search engine pLink 2 with systematic evaluation for proteome-scale identification of cross-linked peptides. *Nat. Commun.* **2019**, *10* (1), 3404. DOI: 10.1038/s41467-019-11337-z.
(14) Schwieters, C. D.; Bermejo, G. A.; Clore, G. M. Xplor-NIH for molecular structure determination from NMR and other data sources. *Protein Sci* **2018**, *27* (1), 26-40. DOI: 10.1002/pro.3248.
(15) Aikawa, K.; Asano, M.; Ono, K.; Habuka, N.; Yano, J.; Wilson, K.; Fujita, H.; Kandori, H.; Hara, T.; Morimoto, M.; et al. Synthesis and biological evaluation of novel selective androgen receptor modulators (SARMs) Part III: Discovery of 4-(5-oxopyrrolidine-1-yl)benzonitrile derivative 2f as a clinical candidate. *Bioorg. Med. Chem.* **2017**, *25* (13), 3330-3349. DOI: 10.1016/j.bmc.2017.04.018.
(16) Gong, Z.; Ding, Y.-H.; Dong, X.; Liu, N.; Zhang, E. E.; Dong, M.-Q.; Tang, C. Visualizing the ensemble structures of protein complexes using chemical cross-linking coupled with mass spectrometry. *Biophys. Rep.* **2016**, *1* (3), 127-138. DOI: 10.1007/s41048-

015-0015-y.

(17) Gong, Z.; Ye, S. X.; Tang, C. Tightening the crosslinking distance restraints for better resolution of protein structure and dynamics. *Structure* **2020**, *28* (10), 1160-1167 e1163. DOI: 10.1016/j.str.2020.07.010.

(18) Franke, D.; Petoukhov, M. V.; Konarev, P. V.; Panjkovich, A.; Tuukkanen, A.; Mertens, H. D. T.; Kikhney, A. G.; Hajizadeh, N. R.; Franklin, J. M.; Jeffries, C. M.; et al. ATSAS 2.8: a comprehensive data analysis suite for small-angle scattering from macromolecular solutions. *J Appl Crystallogr* **2017**, *50*, 1212-1225. DOI: 10.1107/S1600576717007786.

(19) Tria, G.; Mertens, H. D. T.; Kachala, M.; Svergun, D. I. Advanced ensemble modelling of flexible macromolecules using X-ray solution scattering. *Iucrj* **2015**, *2*, 207-217. DOI: 10.1107/S205225251500202x.

(20) Bolger, A. M.; Lohse, M.; Usadel, B. Trimmomatic: a flexible trimmer for Illumina sequence data. *Bioinformatics* **2014**, *30* (15), 2114-2120. DOI: 10.1093/bioinformatics/btu170.

(21) Grabherr, M. G.; Haas, B. J.; Yassour, M.; Levin, J. Z.; Thompson, D. A.; Amit, I.; Adiconis, X.; Fan, L.; Raychowdhury, R.; Zeng, Q.; et al. Full-length transcriptome assembly from RNA-Seq data without a reference genome. *Nat. Biotechnol.* **2011**, *29* (7), 644-652. DOI: 10.1038/nbt.1883.

(22) Kim, D.; Langmead, B.; Salzberg, S. L. HISAT: a fast spliced aligner with low memory requirements. *Nat. Methods* **2015**, *12* (4), 357-360. DOI: 10.1038/nmeth.3317.

(23) Liao, Y.; Smyth, G. K.; Shi, W. The Subread aligner: fast, accurate and scalable read mapping by seed-and-vote. *Nucleic Acids Res.* **2013**, *41* (10), e108. DOI: 10.1093/nar/gkt214.

(24) Love, M. I.; Huber, W.; Anders, S. Moderated estimation of fold change and dispersion for RNA-seq data with DESeq2. *Genome Biol.* **2014**, *15* (12), 550. DOI: 10.1186/s13059-014-0550-8.

(25) Kumar, S.; Stecher, G.; Li, M.; Knyaz, C.; Tamura, K. MEGA X: molecular evolutionary genetics analysis across computing platforms. *Mol. Biol. Evol.* **2018**, *35* (6),

1547-1549. DOI: 10.1093/molbev/msy096.

(26) Letunic, I.; Bork, P. Interactive Tree Of Life (iTOL) v5: an online tool for phylogenetic tree display and annotation. *Nucleic Acids Res.* **2021**, *49* (W1), W293-W296. DOI: 10.1093/nar/gkab301.

(27) Waterhouse, A.; Bertoni, M.; Bienert, S.; Studer, G.; Tauriello, G.; Gumienny, R.; Heer, F. T.; de Beer, T. A. P.; Rempfer, C.; Bordoli, L.; et al. SWISS-MODEL: homology modelling of protein structures and complexes. *Nucleic Acids Res.* **2018**, *46* (W1), W296-W303. DOI: 10.1093/nar/gky427.

(28) Zhang, J.; Huang, X.; Liu, H.; Liu, W.; Liu, J. Novel pathways of endocrine disruption through pesticides interference with human mineralocorticoid receptors. *Toxicol. Sci.* **2018**, *162* (1), 53-63. DOI: 10.1093/toxsci/kfx244.
